# Challenging the Limits of Binarization: A New Scheme Selection Policy Using Reinforcement Learning Techniques for Binary Combinatorial Problem Solving

**DOI:** 10.3390/biomimetics9020089

**Published:** 2024-02-01

**Authors:** Marcelo Becerra-Rozas, Broderick Crawford, Ricardo Soto, El-Ghazali Talbi, Jose M. Gómez-Pulido

**Affiliations:** 1 Escuela de Ingeniería Informática, Pontificia Universidad Católica de Valparaíso, Avenida Brasil 2241, Valparaíso 2362807, Chile; ricardo.soto@pucv.cl; 2 CNRS UMR 9189, Centre de Recherche en Informatique Signal et Automatique de Lille (CRIStAL), University of Lille, F-59000 Lille, France; el-ghazali.talbi@univ-lille.fr; 3 Health Computing and Intelligent Systems Research Group (HCIS), Department of Computer Science, University of Alcalá, 28805 Alcalá de Henares, Spain; jose.gomez@uah.es

**Keywords:** binarization, schemes selection, binary optimization, metaheuristics, reinforcement learning, policy

## Abstract

In this study, we introduce an innovative policy in the field of reinforcement learning, specifically designed as an action selection mechanism, and applied herein as a selector for binarization schemes. These schemes enable continuous metaheuristics to be applied to binary problems, thereby paving new paths in combinatorial optimization. To evaluate its efficacy, we implemented this policy within our BSS framework, which integrates a variety of reinforcement learning and metaheuristic techniques. Upon resolving 45 instances of the Set Covering Problem, our results demonstrate that reinforcement learning can play a crucial role in enhancing the binarization techniques employed. This policy not only significantly outperformed traditional methods in terms of precision and efficiency, but also proved to be extensible and adaptable to other techniques and similar problems. The approach proposed in this article is capable of significantly surpassing traditional methods in precision and efficiency, which could have important implications for a wide range of real-world applications. This study underscores the philosophy behind our approach: utilizing reinforcement learning not as an end in itself, but as a powerful tool for solving binary combinatorial problems, emphasizing its practical applicability and potential to transform the way we address complex challenges across various fields.

## 1. Introduction

Over the past years, optimization has witnessed significant growth, becoming a field of research and application that is increasingly relevant in various sectors. The need to find efficient and optimal solutions has driven researchers and professionals to tackle optimization challenges in diverse fields such as logistics [1], finance [2], engineering [3], healthcare [4], and telecommunications [5], among many others [6,7]. These fields present specific challenges that require customized approaches, leading to the development of new methodologies and optimization algorithms to address complex problems and improve decision-making performance.

To solve these complex optimization problems, various approaches have been employed. Traditional mathematical programming techniques, such as linear programming, integer programming, and dynamic programming, have been widely used to solve well-structured problems with clear mathematical formulations [8]. However, these techniques may face limitations when dealing with large-scale, nonlinear, or combinatorial optimization problems.

In response to the challenges posed by complex optimization problems, metaheuristics have emerged as powerful problem-solving paradigms [9,10,11]. Metaheuristic algorithms are general-purpose optimization algorithms that guide the search for high-quality solutions through an iterative process. They are often inspired by natural phenomena, social behaviors, or biological systems, providing flexible and robust optimization approaches. Moreover, Metaheuristics, such as genetic algorithms, particle swarm optimization, ant colony optimization, and simulated annealing, have proven to be effective in solving a wide range of optimization problems [12].

Metaheuristics excel in handling complex problems with high-dimensional search spaces, nonlinear relationships, and conflicting multiple objectives. They offer advantages such as adaptability, scalability, and the ability to escape local optima [11]. By combining exploration and exploitation strategies, metaheuristics can efficiently navigate the solution space, providing near-optimal or satisfactory solutions within reasonable time frames.

However, it is important to consider the No-Free-Lunch Theorem (NFLT) [13] in optimization. According to the NFLT, there is no universal optimization algorithm that is the best for all problems. This implies that while metaheuristics can be effective in many cases, they do not guarantee the best solution for all optimization problems. The choice of the appropriate metaheuristic depends on the problem characteristics and expert knowledge. In this context, there has been a growing interest in the hybridization of machine learning techniques with metaheuristics in recent years. This combination aims to leverage the learning and adaptation capabilities of machine learning algorithms to enhance the exploration and exploitation of metaheuristics in the search for optimal solutions. These hybridizations may involve the use of machine learning models to guide the search, adapt parameters, or even generate new solutions, opening new possibilities in the field of optimization.

The quest for efficient and effective solutions in reinforcement learning is pivotal in the rapidly advancing field of artificial intelligence. Particularly, the transformation of continuous metaheuristic algorithms into binary formats poses significant challenges, yet offers immense potential in solving complex binary problems. This research is driven by the need to bridge this gap by introducing innovative binarization schemes that enhance the applicability and performance of continuous metaheuristics in binary problem-solving contexts. Our work is motivated by the pressing demand for more adaptable, efficient, and robust methods in the realm of binary combinatorial optimization, a field that is crucial for various real-world applications ranging from logistics to network design.

In the realm of optimization, significant challenges have been encountered, particularly in the adaptation of continuous metaheuristic algorithms to binary representations. This adaptation is crucial for tackling combinatorial optimization problems, yet it presents substantial difficulties. The integration of machine learning with metaheuristics, while offering promising directions, still grapples with the complexities of balancing exploration and exploitation strategies effectively. The existing methodologies often struggle to achieve this balance, leading to either premature convergence or inefficiencies in the optimization process. These challenges underscore the need for innovative approaches in the field.

This work has been inspired by two distinct approaches. The first one involves the adaptation and transformation of continuous metaheuristic algorithms to binary representations, while the second focuses on hybridizations of machine learning with metaheuristics. Our work aligns with the central theme of this Special Issue, focusing on solving difficult optimization problems using nature-inspired computing algorithms. These algorithms, often inspired by real-world phenomena, address optimization problems by simulating physical rules or biological phenomena. In this context, our approach to solving Set Covering Problems using binarization techniques and metaheuristics reflects this spirit. We emulate strategies found in biological systems, like natural and adaptive selection, to develop solutions for complex problems in fields like engineering, optimal control, and deep learning, thus tackling optimization challenges with incomplete information or limited computational power.

The main contributions of this article are summarized as follows:

- Adaptation and transformation of continuous metaheuristic algorithms into binary representations: we have addressed the challenge of converting continuous metaheuristic algorithms into binary formats, which is crucial for solving combinatorial optimization problems.
- Integration of machine learning with metaheuristics to enhance problem solving: we have explored the fusion of machine learning techniques with metaheuristics, holding the promise of significantly improving the capability to solve optimization problems.
- Development of a novel reinforcement learning-based policy for optimization: We have successfully developed an innovative policy based on reinforcement learning that integrates seamlessly into our flexible and generic algorithmic framework. This policy has been successfully applied to solve 45 instances of the Binary Set Covering Problem, demonstrating its competitiveness in terms of solution quality compared to traditional approaches and enabling the automatic adaptation of selected binarization schemes as more instances are solved.

The rest of the paper is structured as follows: Section 2 explains the problem that has been solved and presents a background to optimization problems, metaheuristic techniques, their hybridization, and concepts of machine learning or reinforcement learning. Section 3 introduces the new policy proposed. Section 4 validates its implementation with the obtained results, respective statistical tests and charts. Finally, the paper’s conclusions are presented in Section 5.

## 2. Background and Basic Concepts

### 2.1. Set Covering Problem

The Set Covering Problem (SCP) is a well-known combinatorial optimization problem that arises in various practical applications. It is known that the SCP is NP-hard, which means that finding an optimal solution for large problem instances is computationally challenging.

If we consider a zero-one matrix $A=(a_{i,j})$ of size n×m, a column *j* is said to cover a row *i* if $a_{i,j}=1$. Each column *j* is associated with a non-negative real cost $c_{j}$. Let I=1,…,n and J=1,…,m represent the sets of rows and columns, respectively. The objective of the SCP is to find a subset S⊂J with the minimum total cost, such that each row i∈I is covered by at least one column j∈J.

(1) $$\begin{array}{cccc} \; & \mathrm{Minimize} & \; & \sum_{j\in J}c_{j}x_{j}\\ \; & \mathrm{subject}\;\mathrm{to}\\ \; & \; & & \sum_{j\in J}a_{ij}x_{j}\geq 1,\;\forall i\in I\\ \; & \; & & x_{j}\in \left\{0,1\right\},\;\forall j\in J \end{array}$$

SCP is a problem that can be represented by binary values. Let $x_{j}\in 0,1$, ∀j∈1,…,m. In this binary representation, $x_{j}=1$ if column *j* belongs to the feasible solution. In the case of our algorithm, each particle represents a potential binary solution.

### 2.2. Optimization Problems Helped by Metaheuristics

In general terms, difficult optimization problems are characterized as being intractable for exact deterministic methods within a reasonable time frame, without achieving an optimal solution or a guaranteed bound. These problems can be classified into different categories, based on their continuous or discrete nature, whether they have constraints or not, whether they are single-objective or multi-objective, and whether they are static or dynamic. This is where other specialized approaches come into play, such as metaheuristics, as they offer efficient alternatives to explore the search space and find high-quality solutions. These techniques rely on the exploration and exploitation of the search space and have proven to be valuable in overcoming the limitations of exact methods in solving challenging optimization problems.

Metaheuristics are based on the observation of natural phenomena and the iterative search for optimal or high-quality solutions. Unlike classical optimization algorithms, metaheuristics do not guarantee finding the global optimal solution, but they provide an efficient approximation in situations where conventional techniques are ineffective due to their computational complexity. There are multiple classification criteria for metaheuristics, which can be illustrated by considering aspects such as the search path followed, the use of memory, the type of neighborhood exploration, or the handling of current solutions in each iteration. A detailed classification of metaheuristics can be found in the works of Birattari et al. [14] and Talbi [15]. Another relevant taxonomy presented in [16] delves into these key components.

The fundamental principles of metaheuristics lie in the exploration and exploitation of the search space of a problem. To ensure the success of a metaheuristic in an optimization task, it is essential to find an appropriate balance between these two concepts: exploration and exploitation. Exploration refers to the metaheuristic’s ability to search for solutions in various regions of the search space, aiming to discover new solutions that may be potentially superior. On the other hand, exploitation focuses on gradually improving solutions through refinement. These two aspects are complementary and combine in the iterative process of the metaheuristic, allowing the algorithm to approach optimal or high-quality solutions. The key differences between different metaheuristics lie in the specific approaches they use to achieve this balance [14].

There are several widely used metaheuristics in the scientific literature, such as simulated annealing [17], tabu search [18], genetic algorithms (GA) [19], ant colonies, and particle swarms, to name a few of the most classical ones. Each of these metaheuristics is based on a particular concept or principle, but they share the characteristic of being stochastic and non-deterministic techniques. This means that, although the same problem is solved in different algorithm runs, different solutions can be obtained due to the inherent randomness of metaheuristics.

### 2.3. Discrete and Binary Metaheuristics

As mentioned earlier, while many metaheuristic algorithms excel in continuous search spaces, it is necessary to address discrete and binary optimization problems [9]. To tackle these challenges, several metaheuristic algorithms, such as GA and ACO, have been proposed to solve problems in discrete spaces. The objective of binary optimization is to solve problems where variables are restricted to only take values of “0” and “1”.

Similarly, to solve discrete problems, various binary metaheuristic algorithms have been proposed, such as the Particle Swarm Optimization [20], Grey Wolf Optimization [21], Salp Swarm Optimization [22], and Whale Optimization Algorithm [23], among many others [24].

These techniques, instead of working with continuous values, are particularly suitable for utilizing binary representations and specific search strategies to find optimal solutions in the binary search space. There are various methods to develop the binary version of a continuous metaheuristic. For example, in [25,26], different approaches are proposed where different continuous metaheuristics can be used to solve binary problems. These approaches include Angle Modulation, normalization, the transfer function, the quantum-inspired method, the algebraic or logic operations (XOR) method, the percentile concept, and some hybridizations with genetic algorithms called crossover or with machine learning structures.

### 2.4. Hybrid Metaheuristics

In the scientific literature, there has been discussion about the combination of metaheuristic algorithms (MH) with different learning approaches, such as machine learning and reinforcement learning techniques. This combination is known as hybrid MH [27,28,29]. Blum et al. [30] also present other interesting examples of hybrids with metaheuristics.

When using reinforcement learning (RL) in the context of MHs, we encounter two groups of approaches. In the first group, RL is used to enhance MH, meaning RL is employed to replace specific operators of MH, adjust parameters, perform local searches, or manage the population [31]. These approaches aim to enhance the capabilities of MH by incorporating RL techniques. In the second group, RL is used as a selector of different MHs. In this case, RL is used to choose the most suitable MH for addressing a specific problem. This implies that RL acts as an intelligent selection criterion to determine which MH to use based on the characteristics of the problem at hand [31].

These hybrid approaches between MH and RL offer new perspectives and possibilities to enhance the performance and effectiveness of MH in solving complex problems. The integration of RL into MH can enable adaptability, learning capabilities, and more efficient exploration of the search space, resulting in higher-quality solutions.

### 2.5. Machine Learning: Reinforcement Learning Techniques

Based on Reinforcement Learning (RL), an agent consists of four sub-elements: a policy, a value function, a reward function and, optionally, an environment model [32]. This fundamental framework of RL has been the cornerstone of numerous studies, illustrating its versatility and applicability in various fields. Key examples of such research can be found in the works of researchers who have explored and expanded upon these concepts [33,34]. Building upon this established foundation, we can then offer a definition that encapsulates the essence of Reinforcement Learning in the context of our study:

- The policy of an agent is the way it makes decisions at each moment, establishing a relationship between the perceived situations and the actions it must take. In other words, the policy determines how the agent behaves and which actions it takes based on its environment and the situations it experiences.
- The value function in reinforcement learning enables the agent to have a goal of maximizing the accumulated long-term rewards. This function calculates the value of a state–action pair by estimating the total amount of rewards that the agent can expect to receive in the future, starting from the current state. In other words, the value function assists the agent in making decisions based on an evaluation of its value. While the immediate and direct desirability of a state–action pair is provided by the reward, the value function also takes into account the long-term desirability by considering possible future state–action pairs and their associated rewards. Consequently, the agent utilizes the value as a guide for selecting the action it deems most promising in terms of obtaining higher accumulated rewards in the future.
- The reward function in a reinforcement learning environment establishes the agent’s objective by assigning a numerical value to each observed state–action pair. This reward represents the intrinsic desirability of taking a particular action in a given state. Essentially, the reward function serves as a means of communicating to the agent the desired outcomes without explicitly indicating how to achieve them.
- The environment model is a representation that aims to mimic the behavior of the environment in which the agent operates. This model functions to guide the agent towards the next state and the subsequent reward, based on the current state–action pair. However, it is important to note that the availability of the environment model is not guaranteed, as its usage is optional.

In each discrete time step (t=0,1,2,…,N), the agent and the environment interact. The environment provides the agent with a representation of the current state ($s_{t}$) of the environment, which contains all the relevant information. Based on this representation, the agent selects an action ($a_{t}$) from the available actions in that state ($A(s_{t})$). In the next step (t+1), the agent transitions to a new state ($s_{t+1}$) and receives a reward ($r_{t+1}$) as a result of the action taken ($a_{t}$).

In RL, the agent aims to maximize a value function that represents expected rewards. At each time step, the agent selects the action that is expected to generate the highest sum of future rewards, taking into account the current state. This sum of future rewards, referred to as the expected reward $R_{t}$, is typically defined as a combination of the current reward and discounted future rewards. In summary, the agent makes decisions based on maximizing the expectation of accumulated rewards over time. The equation that represents this is as follows:

(2) $$R_{t}=\sum_{j=0}^{n}\gamma^{j}\cdot r_{t+j+1}$$

The discount factor γ, which ranges from 0 to 1, determines the relative importance of short-term and long-term rewards. The objective of the agent in reinforcement learning is to maximize the long-term rewards while interacting with the environment. To achieve this, the agent needs to learn a policy that determines which actions to take in each state. At each time step, the agent calculates the value of the action-value function $Q^{\pi }\left(s,a\right)$, which represents the expected reward when taking action *a* in state *s* under policy π. This calculation is based on the agent’s accumulated experience, and is used to guide future decision-making. The policy, π, can be defined as:

(3) $$Q^{\pi }\left(s,a\right)=P_{\pi }\left\{r_{t+1}+\gamma \cdot Q^{\pi }\left(s_{t+1},a_{t+1}\right)|s_{t}=s,a_{t}=a\right\}$$

To learn a policy capable of maximizing long-run rewards, the agent has to select the action that fulfills a relation, called a greedy policy.

(4) $$Q^{*}\left(s,a\right)=maxQ^{\pi }\left(s,a\right)$$

This equation states that the value of a state, when following an optimal policy, is equal to the expected return from selecting the best action in that state. Mathematically, it is expressed as $Q^{*}\left(s,a\right)=max_{a\in A(S)}Q^{\pi }\left(s,a\right)$. In other words, the maximum achievable value in a state under an optimal policy is equal to the expected value obtained by selecting the best possible action in that state.

Due to the iterative method, it is possible to compute the action-value function $Q^{\pi }\left(s,a\right)$. It is called policy evaluation, and starts with an arbitrary initialization of $Q_{0}^{\pi }\left(s,a\right)$. At each computational iteration, the action-value function is approximated using Equation (3) as an update rule:

(5) $$Q_{k+1}^{\pi }\left(s,a\right)=P_{\pi }\left\{r_{t+1}+\gamma \cdot Q_{k}^{\pi }\left(s_{t+1},a_{t+1}\right)|s_{t}=s,a_{t}=a\right\}$$

In addition, in each state, it is useful to check whether there is another policy that, if followed, would be able to perform better than the one currently being followed. This process is known as policy improvement. The action that seems the best according to the largest action-value function ($Q^{\pi }\left(s,a\right)$) is selected.

## 3. The New Scheme Selection Policy

Reinforcement learning is a powerful approach in artificial intelligence that enables agents to learn through interaction with a dynamic environment. At the core of reinforcement learning lies the optimal decision-making based on the maximization of cumulative rewards over time.

Despite advances in the field of reinforcement learning, existing policies still have limitations in terms of exploration and exploitation. Excessive exploitation can lead to premature convergence to local optima, while excessive exploration can slow down learning and generate inefficiencies. To overcome these limitations, it is crucial to design more sophisticated policies that strike an optimal balance between exploration and exploitation of available actions in an environment.

In this article, we present a novel reinforcement learning policy that combines intelligent exploration and efficient exploitation to maximize cumulative rewards, thereby addressing current challenges. Our proposal is based on a combination approach of exploration and exploitation strategies, allowing the agent to learn and leverage the most promising actions in a decision-making environment with multiple options.

### 3.1. Selection Policy ‘Roulette–Elitist’

In Section 2.5, action selection represents a pivotal approach. We introduce a mathematical model for such selection, where a subset of the top actions is chosen based on their Q-values, with these values being updated each iteration. The “Roulette–Elitist” policy is inspired by one of the binarization techniques presented in [35]. In this approach, we consider a vector of Q-values for the available actions in a given state. Let there be a set of actions $A=\{a_{1},a_{2},\ldots ,a_{n}\}$ and a corresponding vector of Q-values $Q=[q_{1},q_{2},\ldots ,q_{n}]$, where $q_{i}$ represents the Q-value of the action $a_{i}$.

The process unfolds in each iteration as follows:

1. **Ordering of Q-values:** The Q-values are sorted in descending order. We define a sorting function O(Q) that returns a sequence of indices $I=[i_{1},i_{2},\ldots ,i_{n}]$, ensuring that $q_{i_{1}}\geq q_{i_{2}}\geq \ldots \geq q_{i_{n}}$.
2. **Action selection:** The top 25% of actions are selected based on the ordered vector. The set of selected actions is *S*, where $S=\{a_{i_{j}}|j=1,2,\ldots ,\left\lceil 0.25\times n\right\rceil \}$.
3. **Updating Q-values:** After the iteration, Q-values are updated using the function $U(Q,A^{\prime },R)$, where $A^{\prime }$ represents the set of executed actions and *R* the reward received. The function returns a new vector of Q-values $Q^{\prime }$. See Equation (6).
  (6) $$Q\left(s,a\right)\leftarrow Q\left(s,a\right)+\alpha \left(r+\gamma \max_{a^{\prime }}Q\left(s^{\prime },a^{\prime }\right)-Q\left(s,a\right)\right)$$

This model assumes that both the update function *U* and the mechanism for obtaining rewards *R* are defined in accordance with the reinforcement learning algorithm used. In summary, what we do is order our vector of Q-values from highest to lowest, taking into account the top 25%, i.e., the most promising actions. From this segment, we randomly select an action and proceed with the iterative process of the algorithm, thereafter updating the Q-values vector before repeating this process. To better illustrate this methodology, we have developed Algorithm 1.
**Algorithm 1** Roulette–Elitist Policy Pseudo-Code**Require:**  1: Initialize our Q-Values Vector  2: Initialize the state of our agent**Ensure:** Select an action from the top 25% of actions  3: **for**
*iteration* (*t*) **do**  4:  Sort Q-values (Step 1)  5:  Select action from the top 25% (Step 2)  6:  **for**
*solution* (*i*) **do**  7:   **for**
*dimension* (*d*) **do**  8:    $X_{i,d}^{t+1}\;⟵\;X_{i,d}^{t}\;+\Delta \;\left(a_{t}\right)$  9:   **end for**10:  **end for**11:  Get immediate reward *r_t_*12:  Get the maximum Q-value for the next state *s_t_*_+1_13:  Update Q-values using Equation (6)14:  Update the current state $s_{t}\;⟵\;s_{t+1}$15: **end for**

### 3.2. Agent and Policy: How They Are Used in BSS

Previously, we discussed the application and evaluation of the new policy using the RL and MH techniques employed in our innovative framework named BSS, an acronym for Binarization Schemes Selector. This framework enables the online binarization of a continuous metaheuristic in a fully autonomous manner, following the scheme that the intelligent selector, based on reinforcement learning techniques, deems most appropriate.

In the current section, we will delve into a detailed explanation of why and how the new policy is employed in our system.

#### 3.2.1. Why and How Are We Binarizing?

In Section 2, we discussed that many metaheuristics have been developed for continuous spaces due to their excellent performance and results. We also mentioned that to binarize these continuous metaheuristics, certain approaches are necessary to adapt the metaheuristic to operate in a discrete or binary search space. In this work, and within the BSS framework, we employ the two-step technique, as it provides a tiered approach for the binarization of continuous metaheuristics, allowing for a gradual and controlled transformation of continuous values into binary ones. This is advantageous when one wishes to preserve certain information from the original continuous solutions while operating in a binary space.

The two-step technique for binarizing a continuous metaheuristic into a binary one consists of two main sequential stages: the transfer function and the binarization rule. In the first step, a transfer function is applied to transform continuous values into a discrete interval. This function can be a sigmoidal, logarithmic, or other suitable function that provides a smooth and controlled transformation. The purpose of this stage is to assign values within the discrete interval representing the relative relationship between the original continuous solutions. Table 1 lists all the transfer functions implemented in the BSS.

**Figure 1:**
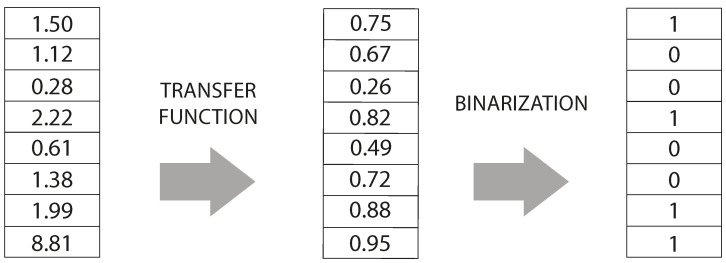
Two-step binarization scheme.

Once the transformation to a discrete interval between 0 and 1 is completed, we proceed to the second step, which is the binarization rule. This rule assigns binary values to the discrete values obtained in the previous step (see Figure 1). A common form of the binarization rule is to use a fixed threshold. For example, if the threshold is 0.5, all values greater than or equal to 0.5 would be assigned as 1 (true), and all values less than 0.5 would be assigned as 0 (false). This binarization rule allows for a binary representation of the solutions from the discrete values. Similar to the transfer functions, our binarization rules can be seen in detail in Table 2.

#### 3.2.2. What Are the Actions Chosen by the Agent?

**Figure 2:**
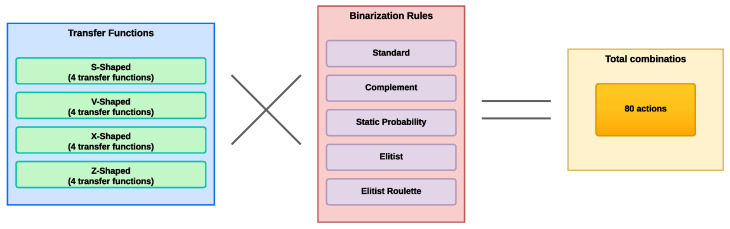
Building actions on the basis of binarization schemes.

In the framework, crucial decisions are made that determine the actions to be undertaken, as previously mentioned. These decisions encompass the selection of the type of transfer function and the binarization rule to be applied, from a wide range of available options. With a total of 80 actions at its disposal, the agent autonomously and online chooses those it deems most appropriate throughout the iterations of the optimization process while solving the given problem. Figure 2 shows how the total number of actions are formed, along with the total number of transfer function families and the total number of binarization rules. It should be noted that each family of transfer function consists of four equations or functions.

### 3.3. Rewards

Rewards in reinforcement learning (RL) algorithms are a critical component in determining performance. This issue is so significant that several methods have been proposed in the literature [42,43,44,45].

Table 3 provides detailed information about the rewards used in various techniques from the literature. The method of evaluating actions, employed by Xu Yue and Song Shin Siang in [42,43], respectively, involves applying a fixed increment or reduction in value for actions that lead to an improvement or lack thereof in overall fitness.

#### 3.3.1. Metrics Acquisition and State Analysis

We previously emphasized the importance of rewards and a proper balance between exploration and exploitation in metaheuristics. In our proposal, these two phases (%XPL and %XPLT) are key states. To operationalize these phases, it is essential to measure their impact, which we achieve through diversity [46]. We have chosen the method of Hussain Kashif et al. [47] to calculate diversity, allowing for an accurate evaluation of the state of diversity in each dimension. This can be mathematically expressed as follows:

(7) $$Div=\frac{1}{l\cdot n}\sum_{d=1}^{l}\sum_{i=1}^{n}\left|{\bar{x}}^{d}-x_{i}^{d}\right|,$$

where Div represents the diversity status determination, ${\bar{x}}^{d}$ denotes the mean values of the individuals in dimension *d*, $x_{i}^{d}$ denotes the value of the *i*-th individual in dimension *d*, *n* denotes the population size, and *l* denotes the size of the individuals dimension.

If we consider the exploration and exploitation percentages to be XPL% and XPLT%, respectively. The percentages XPL% and XPLT% are computed from the study by Morales-Castañeda et al. [48] as follows:

(8) $$XPL\%=\frac{Div}{Div_{max}}\cdot 100,$$

(9) $$XPLT\%=\frac{|Div-Div_{max}|}{Div_{max}}\cdot 100.$$

where Div represents the diversity state determined by Equation (7) and $Div_{max}$ denotes the maximum value of the diversity state discovered throughout the optimization process.

## 4. Experimental Results and Analysis

To analyze the effectiveness of our proposed methodology, we conducted a comparative analysis focusing on various implementations of the CS, PSO, GWO, WOA, and SCA metaheuristics. The results are presented in the Result Table A1, Table A2, Table A3, Table A4 and Table A5 in Appendix A. This study examined various variants of the aforementioned metaheuristics, each featuring a distinctive range of binarization schemes. These encompassed scenarios with 40 and 80 actions, along with a direct comparative analysis between two strategies: the conventional E-greedy policy and our novel proposal, totaling 90 distinct versions.

Among these variants, for example, in the CS metaheuristic, versions such as BCL and MIR were incorporated. These acronyms denote specific combinations of binarization rules and transfer functions (please refer to Table 4). This provided a comprehensive quantitative assessment of their performance. In general, the versions with 80 actions, like BQSA_80a, QL_80a, SA_80a, and MAB_80a, exhibited competitive performance, with Relative Percentage Deviations (RPDs) similar to or, in some cases, better than the 40-action versions, except for CS, where the opposite was observed. However, these differences were not statistically significant, indicating that an increase in the number of actions does not guarantee better performance. Notably, WOA and SCA showed significant disparities in performance compared to the static MIR version, which proved to be the least efficient among the analyzed versions.

The variants were evaluated in terms of performance on 45 specific instances of the Set Covering Problem, instances obtained from Beasley’s OR-Library [50]. In Table 5, we provide detailed information about the configuration of the SCP instances used in our analysis. For each variant, three main parameters were evaluated: optimal performance (Best), average performance (Average), and the Relative Percentage Deviation (RPD) in relation to a known optimal solution (Opt.). The calculation of RPD is explained in the following equation:

(10) $$\mathrm{RPD}=\frac{100\times (Best-Opt)}{Opt}.$$

Regarding the algorithmic development, Python 3.8 was chosen as the programming language, utilizing the complimentary services of Google Colaboratory, as documented by Bisong [51]. The data accrued were archived and processed utilizing the databases of the Google Cloud platform. Adhering to the guidelines proposed by Lanza [49], which recommend 40,000 calls to the objective function, the procedure was configured with a population of 40 individuals and 1000 iterations for each execution of the CS, PSO, GWO, SCA, and WOA algorithms. Each instance underwent 31 independent executions to ensure the reliability of the results. The specific parameters employed for each of the aforementioned metaheuristics, including CS, PSO, GWO, SCA, WOA, BQSA, QL, SA, MAB, are comprehensively delineated in Table 6. This comparative analysis is crucial for understanding which configurations are most beneficial in various situations and how metaheuristics can be optimized to address specific optimization problems. Furthermore, the results obtained not only provide a comprehensive view of the efficacy of different configurations, but also serve as a guide for future research and practical applications of these advanced optimization techniques.

### 4.1. Statistical Tests

Table A6, Table A7, Table A8, Table A9 and Table A10 in Appendix B collectively present an exhaustive statistical analysis, comparing the performance of a broad spectrum of algorithms, including MAB, BQSA, SA, QL, BCL, and MIR, across the implementations of CS, PSO, GWO, SCA, and WOA. These analyses are conducted in various configurations, covering scenarios with 40 and 80 actions, and contrasting two distinct policy approaches: our innovative proposal and the traditional E-greedy strategy. The tables exhibit the average *p*-value results from pairwise comparisons, utilizing the Wilcoxon–Mann–Whitney test [52] to assess the statistical significance of performance differences among the algorithmic variants.

In these analyses, a *p*-value threshold of 0.05 demarcates the boundary for statistical significance. Values equal to or greater than 0.05, as presented in the tables, imply that there is no significant performance disparity among the algorithms. In contrast, *p*-values less than 0.05, highlighted in bold, signify statistically significant differences in performance. This comprehensive evaluation not only underscores the relative strengths and weaknesses of these algorithms, but also provides valuable insights into their suitability for different configurations and contexts. For example, it can be noted that the majority of the metaheuristics exhibit statistically distinct performance compared to algorithms like BCL or MIR across all configurations, as indicated by *p*-values lower than 0.05. In contrast, when compared with other variants such as MAB, QL, BQSA, or SA in their 80-action versions, different behaviors are observed. Another notable observation from the results is that for the 40-action variants of MAB and SA using the E-greedy policy (pl4), there is a statistically significant difference between the 40-action and 80-action versions, as well as with our proposed policy. This behavior is observable in PSO, GWO, SCA, and WOA.

### 4.2. Exploration–Exploitation and Convergence Charts

**Figure A1:**
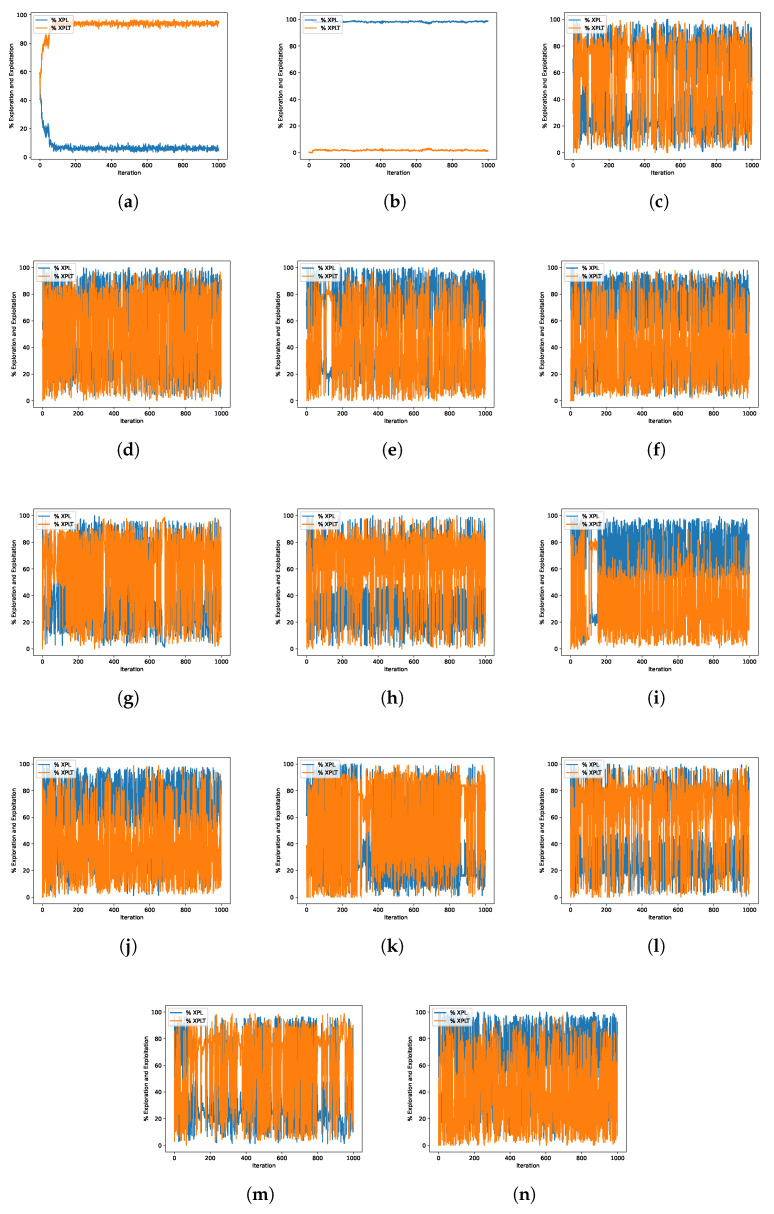

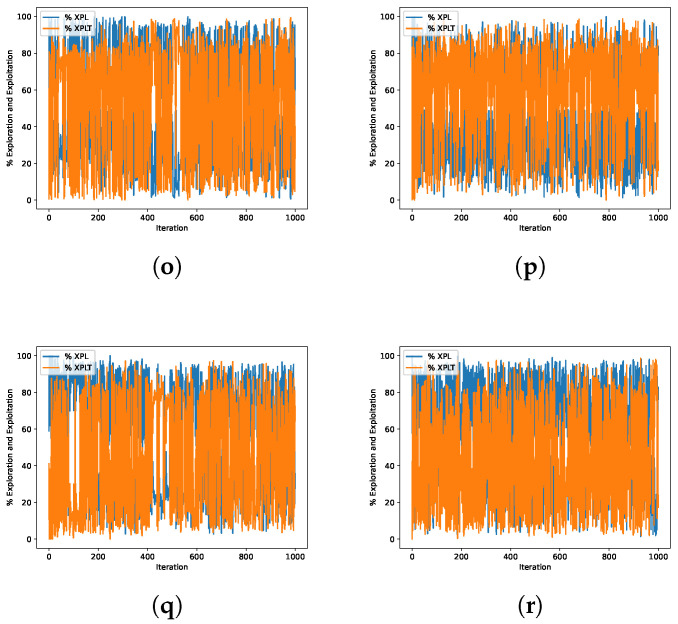
Exploration–Exploitation charts for the instance scp55 using CS. Instance Optimum: 211. (**a**) BCL-Best: 212, (**b**) MIR-Best: 331, (**c**) BQSA_40a_pl0-Best: 211, (**d**) BQSA_40a_pl4-Best: 211, (**e**) BQSA_80a_pl0-Best: 211, (**f**) BQSA_80a_pl4-Best: 211, (**g**) SA_40a_pl0-Best: 211, (**h**) SA_40a_pl4-Best: 211, (**i**) SA_80a_pl0-Best: 211, (**j**) SA_80a_pl4-Best: 212, (**k**) MAB_40a_pl0-Best: 211, (**l**) MAB_40a_pl4-Best: 211, (**m**) MAB_80a_pl0-Best: 211, (**n**) MAB_80a_pl4-Best: 211. (**o**) QL_40a_pl0-Best: 211, (**p**) QL_40a_pl4-Best: 211, (**q**) QL_80a_pl0-Best: 211, (**r**) QL_80a_pl4-Best: 211.

**Figure A2:**
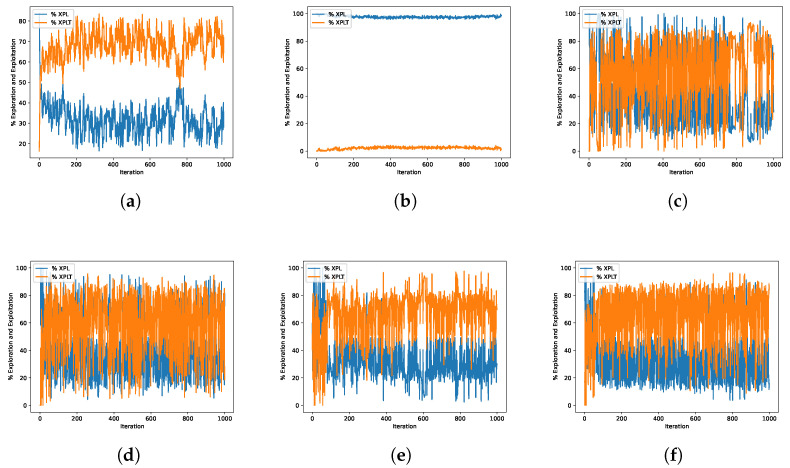

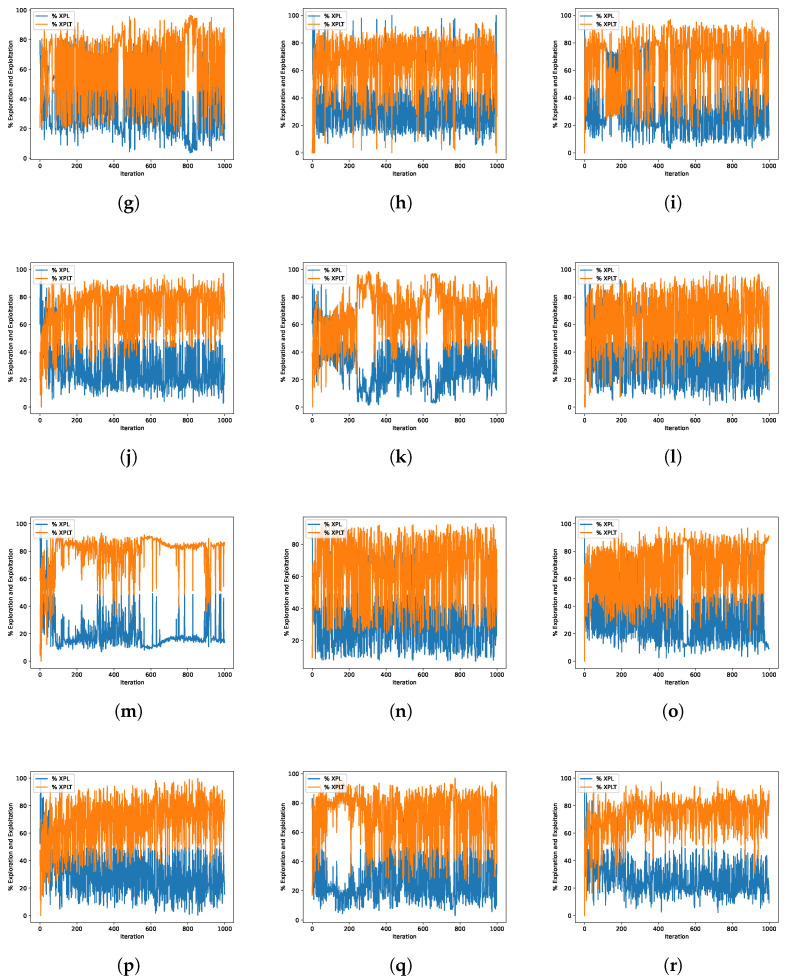
Exploration–Exploitation charts for the instance scp55 using PSO. Instance Optimum: 211.(**a**) BCL-Best: 212, (**b**) MIR-Best: 284, (**c**) BQSA_40a_pl0-Best: 212, (**d**) BQSA_40a_pl4-Best: 213, (**e**) BQSA_80a_pl0-Best: 212, (**f**) BQSA_80a_pl4-Best: 211, (**g**) SA_40a_pl0-Best: 212, (**h**) SA_40a_pl4-Best: 212, (**i**) SA_80a_pl0-Best: 212, (**j**) SA_80a_pl4-Best: 212, (**k**) MAB_40a_pl0-Best: 211, (**l**) MAB_40a_pl4-Best: 212, (**m**) MAB_80a_pl0-Best: 212, (**n**) MAB_80a_pl4-Best: 211, (**o**) QL_40a_pl0-Best: 212, (**p**) QL_40a_pl4-Best: 212, (**q**) QL_80a_pl0-Best: 211, (**r**) QL_80a_pl4-Best: 212.

**Figure A3:**
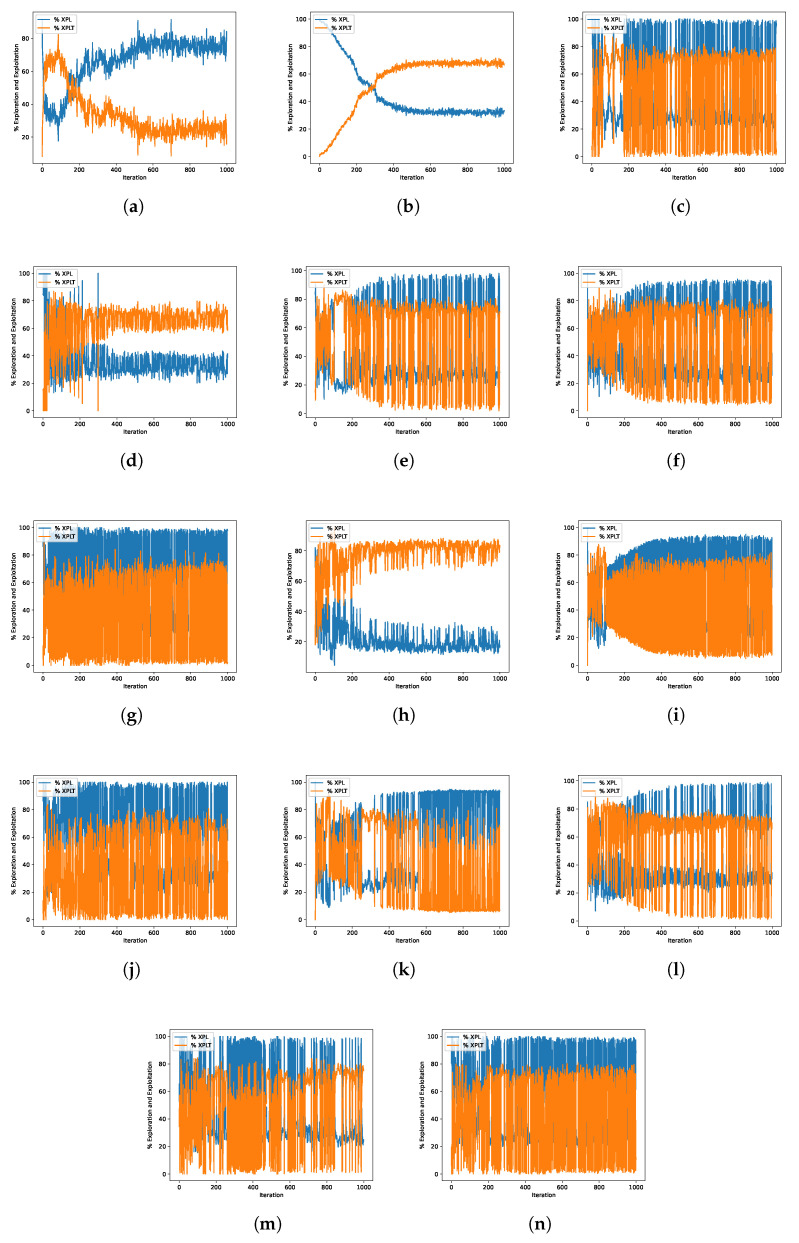

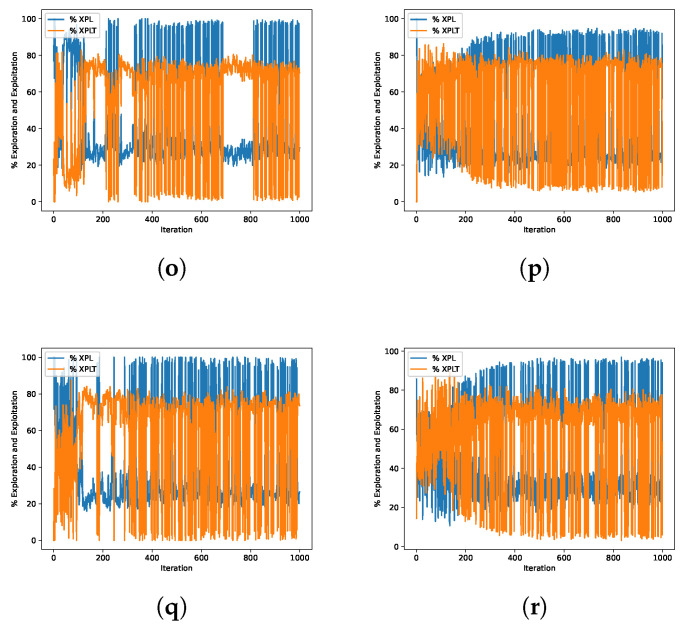
Exploration–Exploitation charts for the instance scp55 using GWO. Instance Optimum: 211. (**a**) BCL-Best: 212, (**b**) MIR-Best: 216, (**c**) BQSA_40a_pl0-Best: 212, (**d**) BQSA_40a_pl4-Best: 212, (**e**) BQSA_80a_pl0-Best: 211, (**f**) BQSA_80a_pl4-Best: 212, (**g**) SA_40a_pl0-Best: 212, (**h**) SA_40a_pl4-Best: 212, (**i**) SA_80a_pl0-Best: 212, (**j**) SA_80a_pl4-Best: 211, (**k**) MAB_40a_pl0-Best: 212, (**l**) MAB_40a_pl4-Best: 213, (**m**) MAB_80a_pl0-Best: 212, (**n**) MAB_80a_pl4-Best: 212. (**o**) QL_40a_pl0-Best: 212, (**p**) QL_40a_pl4-Best: 212, (**q**) QL_80a_pl0-Best: 213, (**r**) QL_80a_pl4-Best: 212.

**Figure A4:**
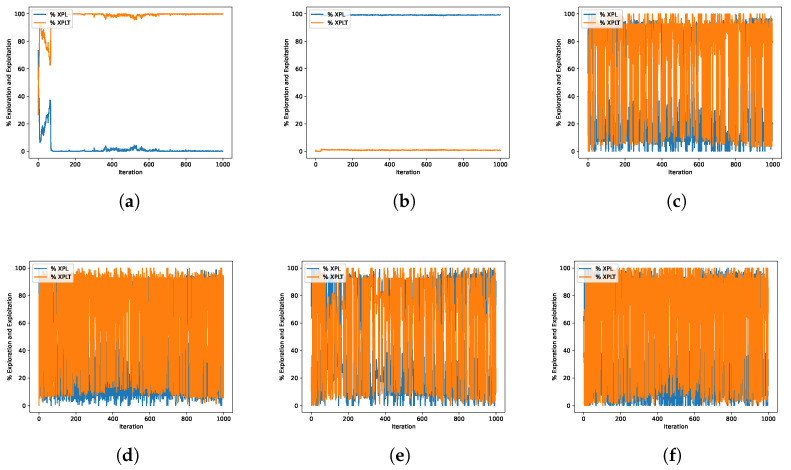

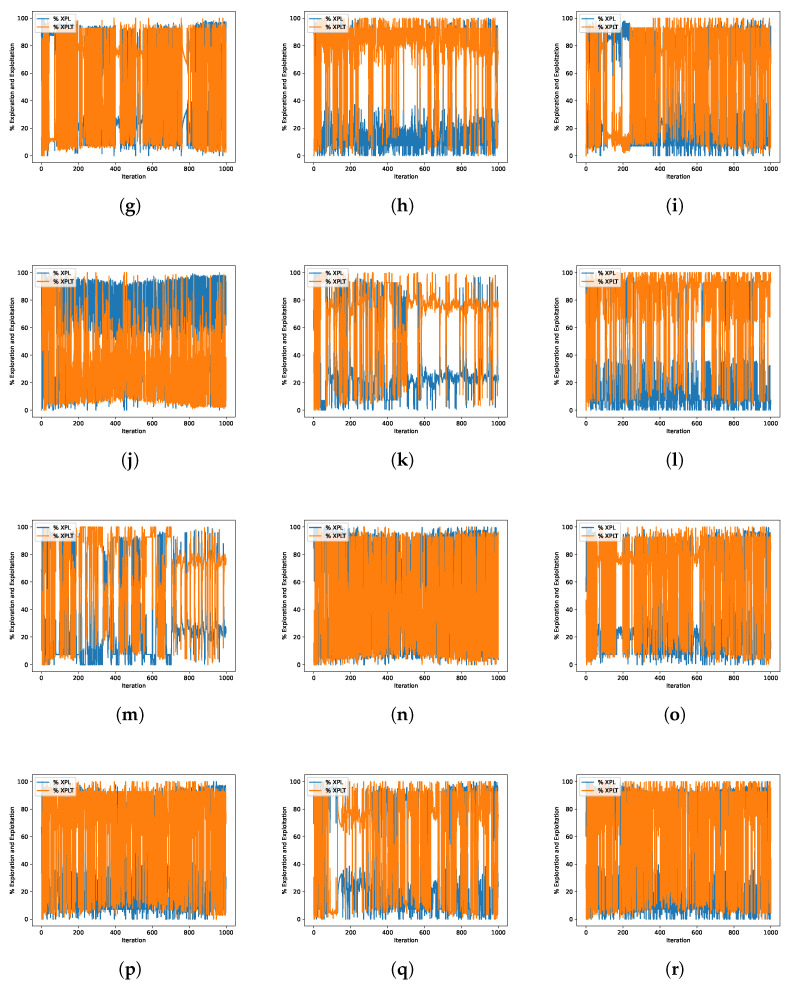
Exploration–Exploitation charts for the instance scp55 using SCA. Instance Optimum: 211. (**a**) BCL-Best: 224, (**b**) MIR-Best: 411, (**c**) BQSA_40a_pl0-Best: 211, (**d**) BQSA_40a_pl4-Best: 211, (**e**) BQSA_80a_pl0-Best: 211, (**f**) BQSA_80a_pl4-Best: 211, (**g**) SA_40a_pl0-Best: 211, (**h**) SA_40a_pl4-Best: 211, (**i**) SA_80a_pl0-Best: 211, (**j**) SA_80a_pl4-Best: 211, (**k**) MAB_40a_pl0-Best: 211, (**l**) MAB_40a_pl4-Best: 211, (**m**) MAB_80a_pl0-Best: 211, (**n**) MAB_80a_pl4-Best: 211, (**o**) QL_40a_pl0-Best: 211, (**p**) QL_40a_pl4-Best: 211, (**q**) QL_80a_pl0-Best: 211, (**r**) QL_80a_pl4-Best: 211.

**Figure A5:**
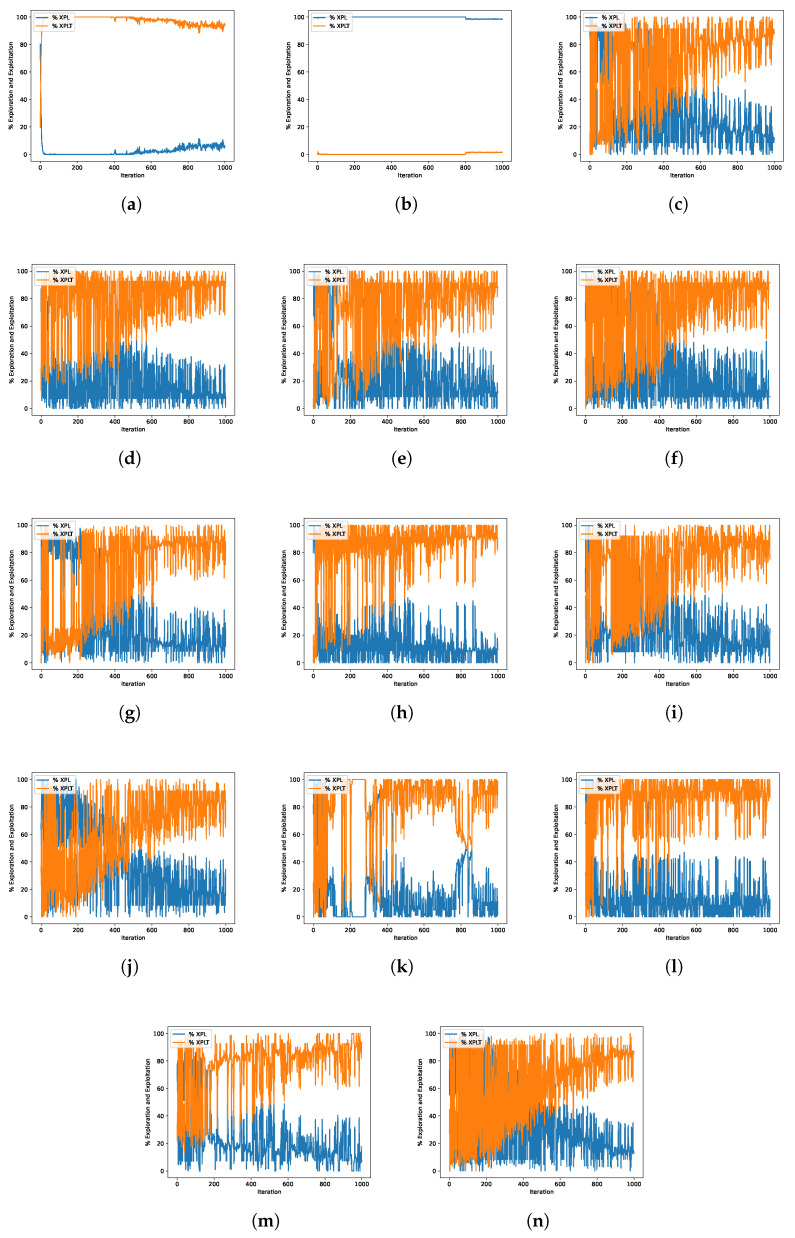

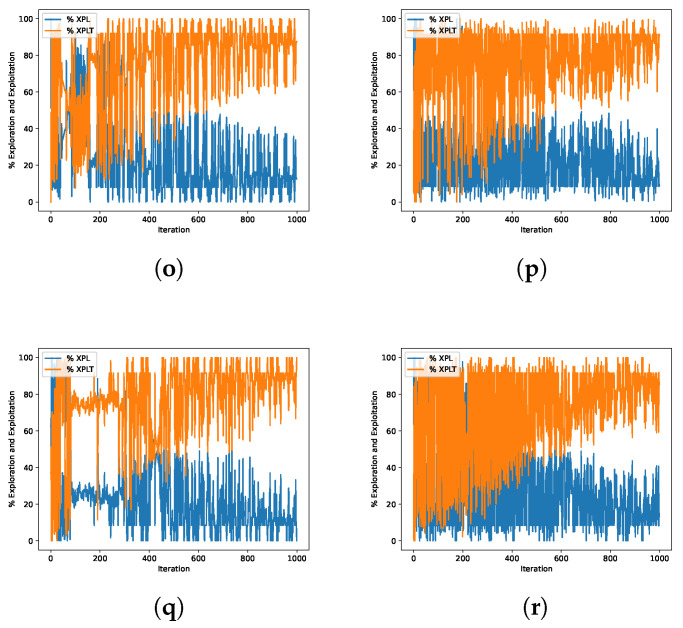
Exploration–Exploitation charts for the instance scp55 using WOA. Instance Optimum: 211. (**a**) BCL-Best: 294, (**b**) MIR-Best: 397, (**c**) BQSA_40a_pl0-Best: 211, (**d**) BQSA_40a_pl4-Best: 211, (**e**) BQSA_80a_pl0-Best: 212, (**f**) BQSA_80a_pl4-Best: 211, (**g**) SA_40a_pl0-Best: 211, (**h**) SA_40a_pl4-Best: 211, (**i**) SA_80a_pl0-Best: 211, (**j**) SA_80a_pl4-Best: 211, (**k**) MAB_40a_pl0-Best: 211, (**l**) MAB_40a_pl4-Best: 212, (**m**) MAB_80a_pl0-Best: 211, (**n**) MAB_80a_pl4-Best: 211. (**o**) QL_40a_pl0-Best: 211, (**p**) QL_40a_pl4-Best: 211, (**q**) QL_80a_pl0-Best: 211, (**r**) QL_80a_pl4-Best: 211.

Figure A1, Figure A2, Figure A3, Figure A4 and Figure A5 in Appendix C present graphs depicting Exploration and Exploitation, where the X-axis represents the number of iterations, and the Y-axis displays the percentages of exploration (XPL) and exploitation (XPLT), percentages obtained as indicated in Section 3.3.1. These graphs offer a unique perspective on the behavior during the search process. They highlight the trends toward exploitation in the variants with 80 actions and the nuances in the shifts between exploration and exploitation in the search processes based on BSS. This is crucial for understanding the diversity among individuals during optimization, as described in the cited literature.

**Figure A6:**
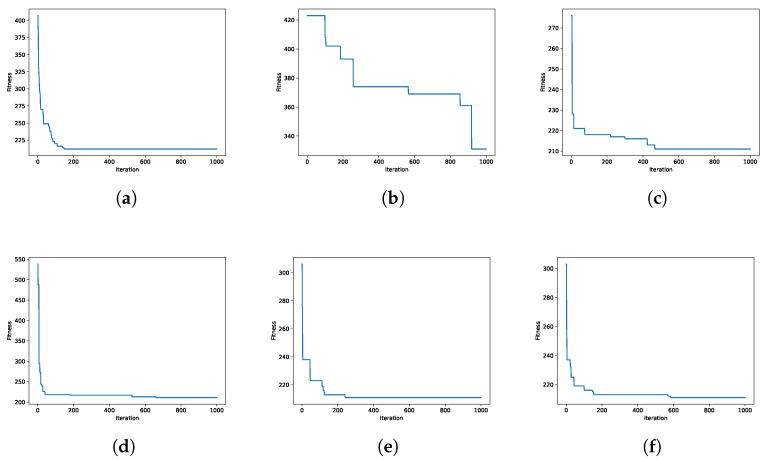

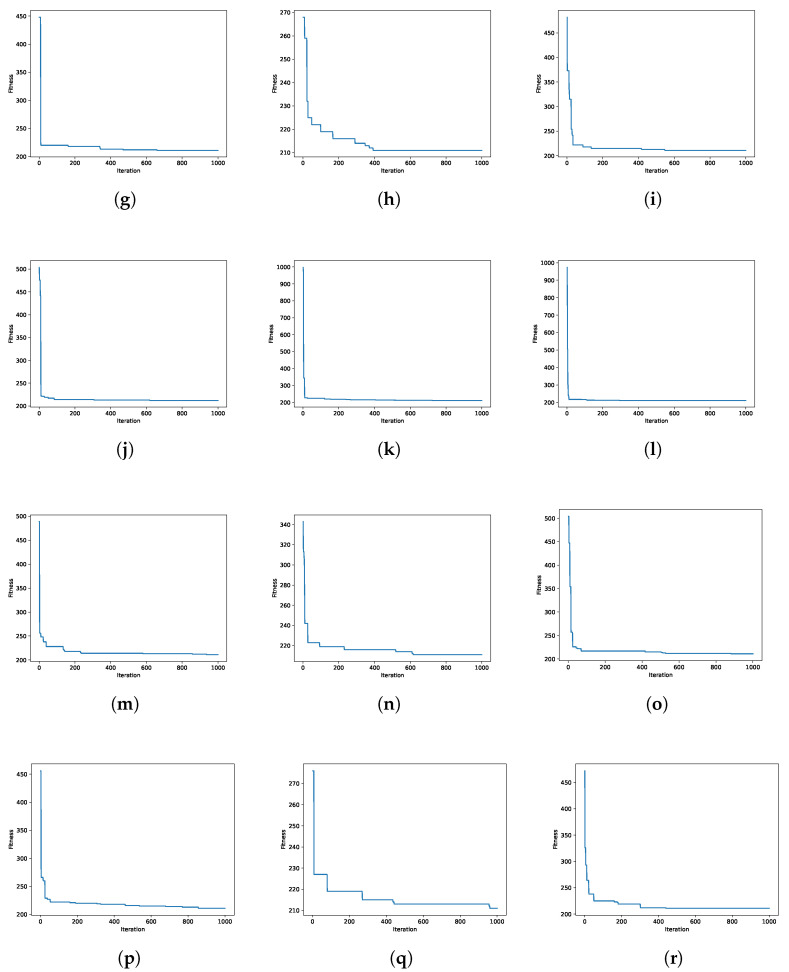
Convergence graphs for the instance scp55 using CS. Instance Optimum: 211. (**a**) BCL-Best: 212, (**b**) MIR-Best: 331, (**c**) BQSA_40a_pl0-Best: 211, (**d**) BQSA_40a_pl4-Best: 211, (**e**) BQSA_80a_pl0-Best: 211, (**f**) BQSA_80a_pl4-Best: 211, (**g**) SA_40a_pl0-Best: 211, (**h**) SA_40a_pl4-Best: 211, (**i**) SA_80a_pl0-Best: 211, (**j**) SA_80a_pl4-Best: 212, (**k**) MAB_40a_pl0-Best: 211, (**l**) MAB_40a_pl4-Best: 211, (**m**) MAB_80a_pl0-Best: 211, (**n**) MAB_80a_pl4-Best: 211, (**o**) QL_40a_pl0-Best: 211, (**p**) QL_40a_pl4-Best: 211, (**q**) QL_80a_pl0-Best: 211, (**r**) QL_80a_pl4-Best: 211.

**Figure A7:**
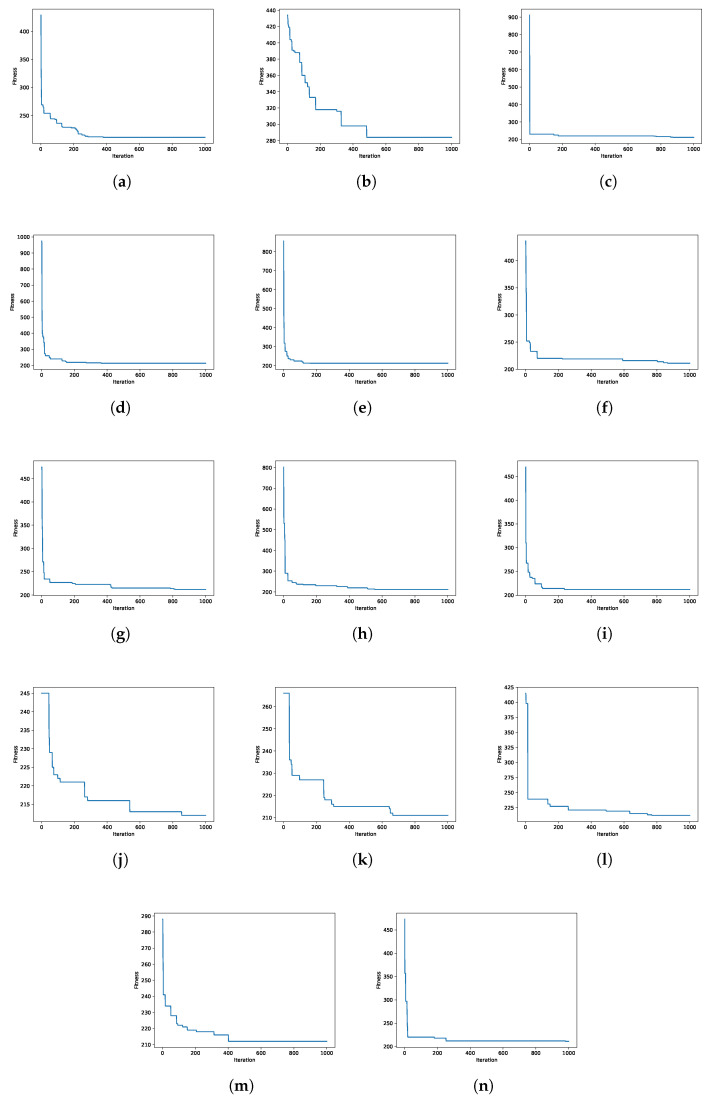

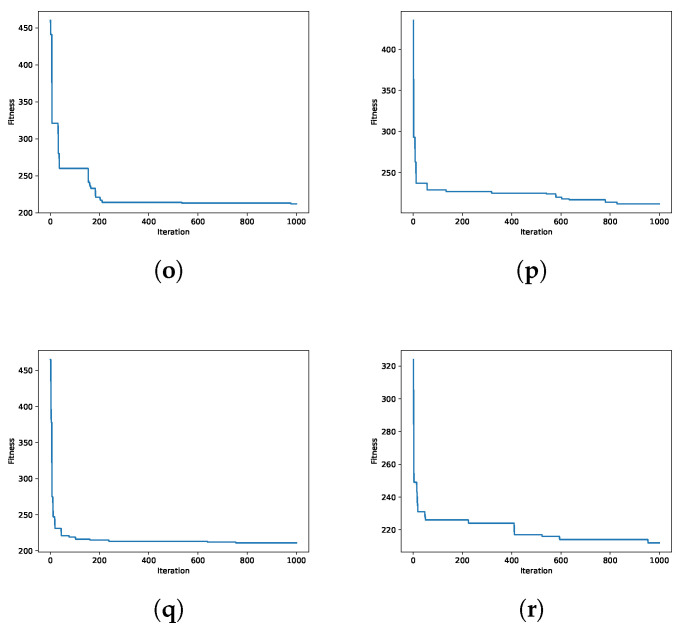
Convergence graphs for the instance scp55 using PSO. Instance Optimum: 211. (**a**) BCL-Best: 212, (**b**) MIR-Best: 284, (**c**) BQSA_40a_pl0-Best: 212, (**d**) BQSA_40a_pl4-Best: 213, (**e**) BQSA_80a_pl0-Best: 212, (**f**) BQSA_80a_pl4-Best: 211, (**g**) SA_40a_pl0-Best: 212, (**h**) SA_40a_pl4-Best: 212, (**i**) SA_80a_pl0-Best: 212, (**j**) SA_80a_pl4-Best: 212, (**k**) MAB_40a_pl0-Best: 211, (**l**) MAB_40a_pl4-Best: 212, (**m**) MAB_80a_pl0-Best: 212, (**n**) MAB_80a_pl4-Best: 211. (**o**) QL_40a_pl0-Best: 212, (**p**) QL_40a_pl4-Best: 212, (**q**) QL_80a_pl0-Best: 211, (**r**) QL_80a_pl4-Best: 212.

**Figure A8:**
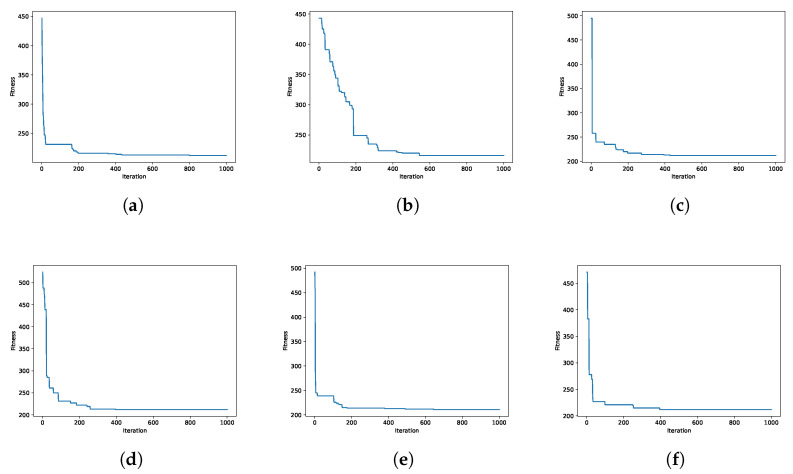

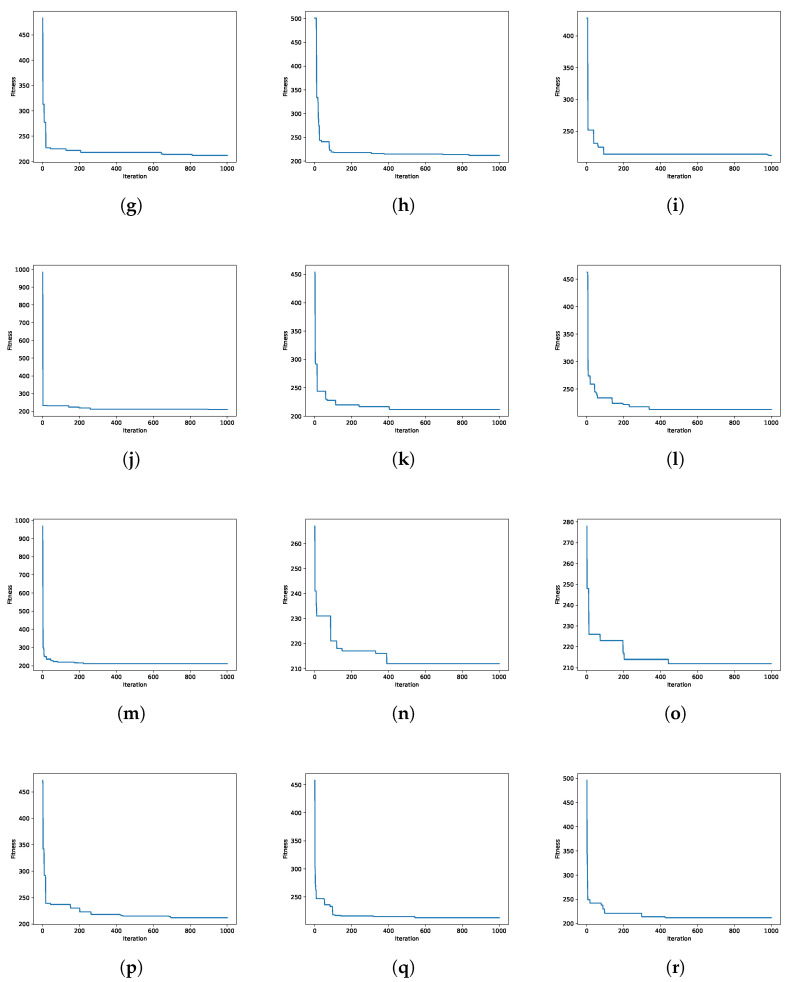
Convergence graphs for the instance scp55 using GWO. Instance Optimum: 211. (**a**) BCL-Best: 212, (**b**) MIR-Best: 216, (**c**) BQSA_40a_pl0-Best: 212, (**d**) BQSA_40a_pl4-Best: 212, (**e**) BQSA_80a_pl0-Best: 211, (**f**) BQSA_80a_pl4-Best: 212, (**g**) SA_40a_pl0-Best: 212, (**h**) SA_40a_pl4-Best: 212, (**i**) SA_80a_pl0-Best: 212, (**j**) SA_80a_pl4-Best: 211, (**k**) MAB_40a_pl0-Best: 212, (**l**) MAB_40a_pl4-Best: 213, (**m**) MAB_80a_pl0-Best: 212, (**n**) MAB_80a_pl4-Best: 212, (**o**) QL_40a_pl0-Best: 212, (**p**) QL_40a_pl4-Best: 212, (**q**) QL_80a_pl0-Best: 213, (**r**) QL_80a_pl4-Best: 212.

**Figure A9:**
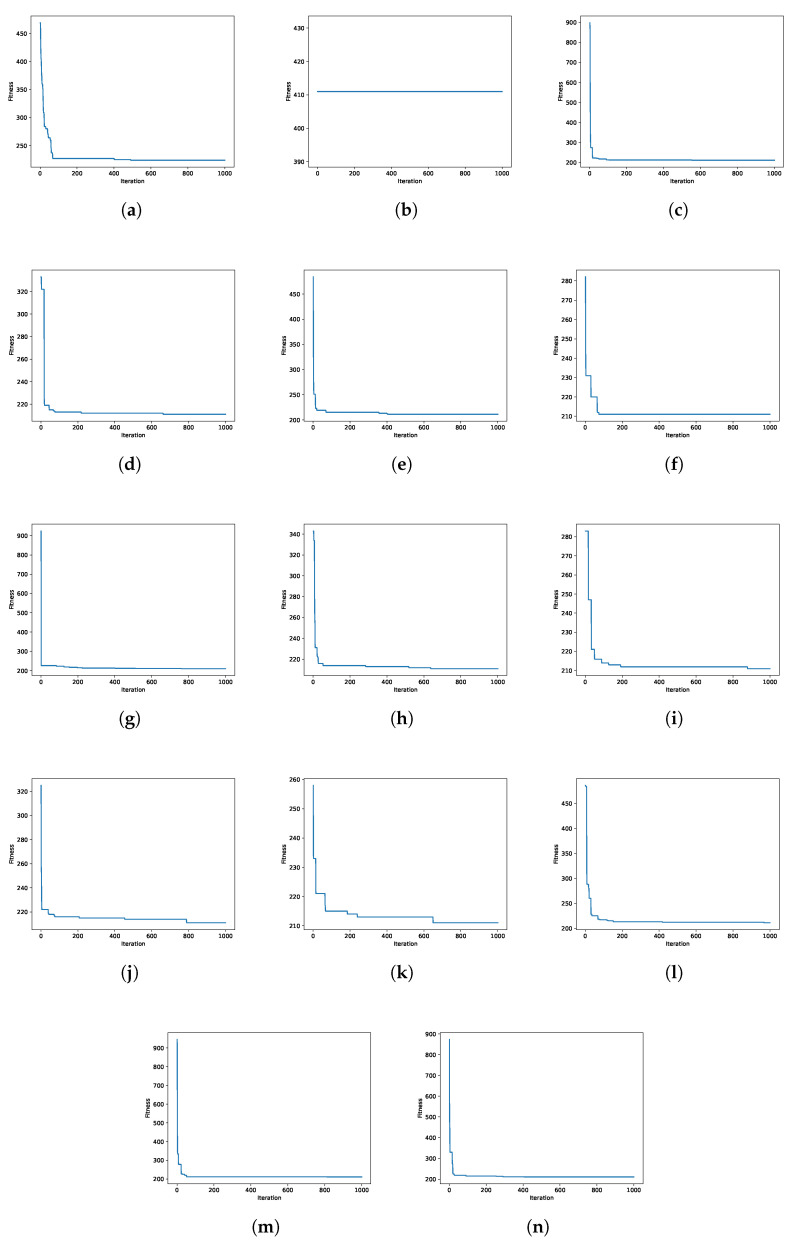

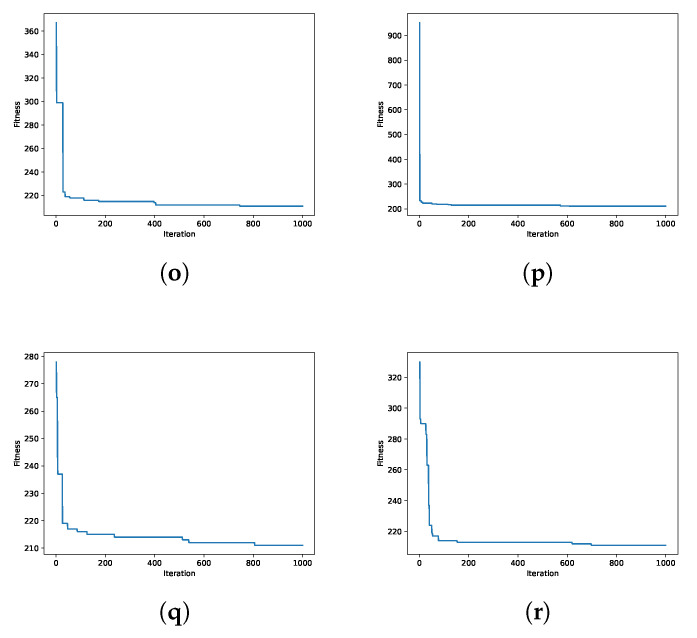
Convergence graphs for the instance scp55 using SCA. Instance Optimum: 211. (**a**) BCL-Best: 224, (**b**) MIR-Best: 411, (**c**) BQSA_40a_pl0-Best: 211, (**d**) BQSA_40a_pl4-Best: 211, (**e**) BQSA_80a_pl0-Best: 211, (**f**) BQSA_80a_pl4-Best: 211, (**g**) SA_40a_pl0-Best: 211, (**h**) SA_80a_pl4-Best: 211, (**i**) SA_80a_pl0-Best: 211, (**j**) SA_80a_pl4-Best: 211, (**k**) MAB_40a_pl0-Best: 211, (**l**) MAB_40a_pl4-Best: 211, (**m**) MAB_80a_pl0-Best: 211, (**n**) MAB_80a_pl4-Best: 211. (**o**) QL_40a_pl0-Best: 211, (**p**) QL_40a_pl4-Best: 211, (**q**) QL_80a_pl0-Best: 211, (**r**) QL_80a_pl4-Best: 211.

**Figure A10:**
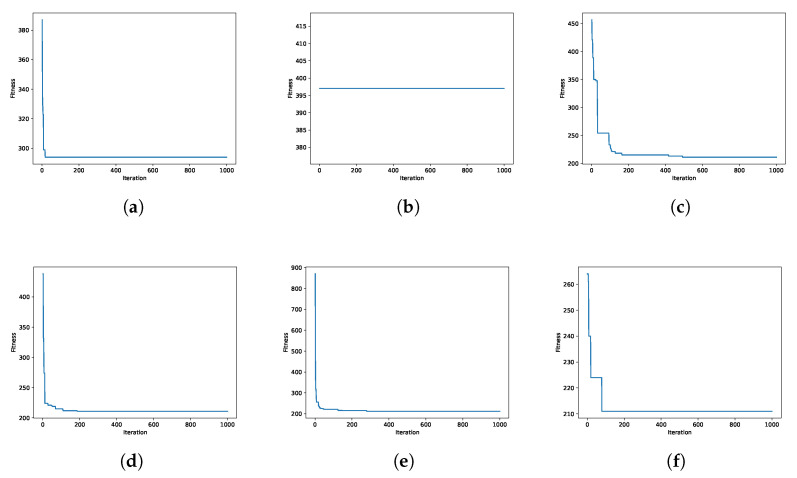

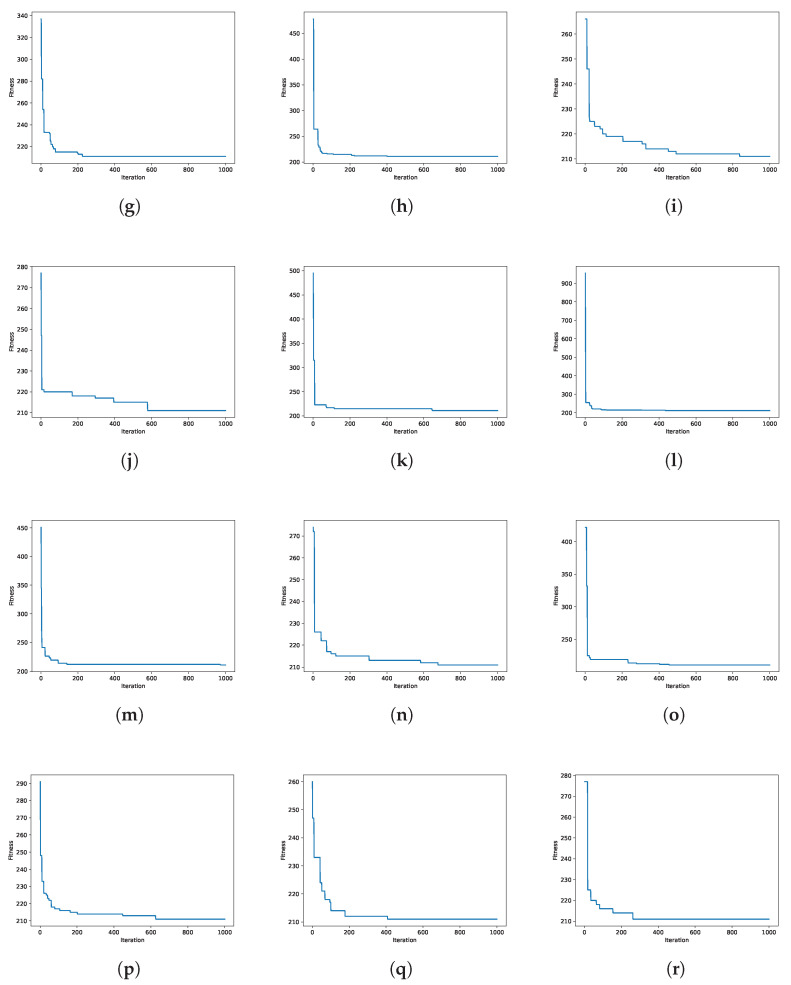
Convergence graphs for the instance scp55 using WOA. Instance Optimum: 211. (**a**) BCL-Best: 294, (**b**) MIR-Best: 397, (**c**) BQSA_40a_pl0-Best: 211, (**d**) BQSA_40a_pl4-Best: 211, (**e**) BQSA_80a_pl0-Best: 212, (**f**) BQSA_80a_pl4-Best: 211, (**g**) SA_40a_pl0-Best: 211, (**h**) SA_80a_pl4-Best: 211, (**i**) SA_80a_pl0-Best: 211, (**j**) SA_80a_pl4-Best: 211, (**k**) MAB_40a_pl0-Best: 211, (**l**) MAB_40a_pl4-Best: 212, (**m**) MAB_80a_pl0-Best: 211, (**n**) MAB_80a_pl4-Best: 211, (**o**) QL_40a_pl0-Best: 211, (**p**) QL_40a_pl4-Best: 211, (**q**) QL_80a_pl0-Best: 211, (**r**) QL_80a_pl4-Best: 211.

Based on this, data from the experimental phase, including fitness and diversity metrics, were meticulously recorded and analyzed. The convergence graphs (Figure A6, Figure A7, Figure A8, Figure A9 and Figure A10 in Appendix D) underscore these findings, showing the achieved fitness (Y-axis) over iterations (X-axis). These findings are particularly enlightening for understanding performance divergences in methods like MIR and BCL, revealing lower efficiency in convergence compared to other variants.

A key observation from this analysis is the pattern of convergence and the balance between exploration and exploitation processes during the search, which aligns with the findings of previous studies by Morales [48]. This aligns with the assertion that strategies effective in one context may not be universally applicable, consistent with the principles of the No-Free-Lunch Theorem. This study reinforces the idea that the selection of metaheuristics and their configurations must be tailored according to the unique characteristics of each problem to be solved.

## 5. Conclusions

This paper presented an innovative approach for solving binary combinatorial problems using reinforcement learning techniques. The primary objective of this work was to propose a novel action selection policy, which was employed and applied to enhance the efficiency and accuracy of pre-existing binarization schemes within our framework. This had significant implications for a broad spectrum of real-world applications.

The proposed approach was founded upon the selection of binarization schemes through the utilization of various reinforced learning techniques featuring distinct action selection policies. This enabled continuous metaheuristics to discover optimal solutions for intricate combinatorial problems. The results of the tests conducted in this study indicated that the proposed approach significantly outperformed conventional methods in terms of accuracy and efficiency. Furthermore, distinctive performance patterns were observed in the implemented algorithms, particularly when compared to static techniques.

We believe that this work could bear substantial implications for the field of reinforcement learning. Specifically, this study demonstrated that the utilization of reinforcement learning techniques could serve as a potent tool for solving binary combinatorial problems. The approach proffered in this paper demonstrated a remarkable capacity to outperform traditional methods significantly with regard to accuracy and efficiency, which could hold crucial implications for a wide array of real-world applications.

This final point also harmonized with our future endeavors, where the potential application of this approach to other categories of combinatorial problems, as well as more general optimization issues, could be explored. Additionally, the feasibility of amalgamating this approach with other machine learning techniques, such as deep learning, to further augment the efficiency and precision of models, could be subject to investigation. Furthermore, a detailed analysis of the time complexity for our proposed method is another avenue we aim to explore in our future research. This would not only enhance our understanding of the theoretical underpinnings of our approach, but also provide a comprehensive benchmark against which its performance can be evaluated, especially in comparison to existing methods. Such an investigation would significantly contribute to the robustness and reliability of our findings, offering a more rounded view of the method’s applicability and scalability in diverse computational scenarios.

## Figures and Tables

**Table 1:** Transfer functions.

Transfer Functions							
**S-Shaped [36]**		**V-Shaped [36,37]**		**X-Shaped [38,39]**		**Z-Shaped [40,41]**	
Name	Equation	Name	Equation	Name	Equation	Name	Equation
S1	$T\left(d_{w}^{j}\right)=\frac{1}{1+e^{-2d_{w}^{j}}}$	V1	$T\left(d_{w}^{j}\right)=\left|erf\left(\frac{\sqrt{\pi }}{2}d_{w}^{j}\right)\right|$	X1	$T\left(d_{w}^{j}\right)=\frac{1}{1+e^{2d_{w}^{j}}}$	Z1	$T\left(d_{w}^{j}\right)=\sqrt{1-2^{d_{w}^{j}}}$
S2	$T\left(d_{w}^{j}\right)=\frac{1}{1+e^{-d_{w}^{j}}}$	V2	$T\left(d_{w}^{j}\right)=\left|tanh(d_{w}^{j})\right|$	X2	$T\left(d_{w}^{j}\right)=\frac{1}{1+e^{d_{w}^{j}}}$	Z2	$T\left(d_{w}^{j}\right)=\sqrt{1-5^{d_{w}^{j}}}$
S3	$T\left(d_{w}^{j}\right)=\frac{1}{1+e^{\frac{-d_{w}^{j}}{2}}}$	V3	$T\left(d_{w}^{j}\right)=\left|\frac{d_{w}^{j}}{\sqrt{1+{\left(d_{w}^{j}\right)}^{2}}}\right|$	X3	$T\left(d_{w}^{j}\right)=\frac{1}{1+e^{\frac{d_{w}^{j}}{2}}}$	Z3	$T\left(d_{w}^{j}\right)=\sqrt{1-8^{d_{w}^{j}}}$
S4	$T\left(d_{w}^{j}\right)=\frac{1}{1+e^{\frac{-d_{w}^{j}}{3}}}$	V4	$T\left(d_{w}^{j}\right)=\left|\frac{2}{\pi }arctan\left(\frac{\pi }{2}d_{w}^{j}\right)\right|$	X4	$T\left(d_{w}^{j}\right)=\frac{1}{1+e^{\frac{d_{w}^{j}}{3}}}$	Z4	$T\left(d_{w}^{j}\right)=\sqrt{1-20^{d_{w}^{j}}}$

**Table 2:** Binarization functions.

Type	Binarization
Standard	$X_{new}^{j}=\left\{\begin{array}{cc} 1 & if\;rand\leq T(d_{w}^{j})\\ 0 & else. \end{array}\right.$
Complement	$X_{new}^{j}=\left\{\begin{array}{cc} X_{w}^{j} & if\;rand\leq T(d_{w}^{j})\\ 0 & else. \end{array}\right.$
Static Probability	$X_{new}^{j}=\left\{\begin{array}{cc} 0 & if\;T(d_{w}^{j})\leq \alpha \\ X_{w}^{j} & if\;\alpha <T\left(d_{w}^{j}\right)\leq \frac{1}{2}\left(1+\alpha \right)\\ 1 & if\;T\left(d_{w}^{j}\right)\geq \frac{1}{2}\left(1+\alpha \right) \end{array}\right.$
Elitist	$X_{new}^{j}=\left\{\begin{array}{cc} X_{Best}^{j} & if\;rand<T(d_{w}^{j})\\ 0 & else. \end{array}\right.$
Roulette Elitist	$X_{new}^{j}=\left\{\begin{array}{cc} P\left[X_{new}^{j}=\zeta_{j}\right]=\frac{f(\zeta )}{\sum_{\delta \in Q_{g}}f\left(\delta \right)} & \mathrm{if}\;\mathrm{rand}\;\leq T(d_{w}^{j})\\ P[X_{new}^{j}=0]=1 & else. \end{array}\right.$

**Table 3:** Reward types.

Reward Types	Mathematical Formula
With Penalty	$R_{t}=\left\{\begin{array}{cc} +1, & \mathrm{If}\;\mathrm{fitness}\;\mathrm{improves}\\ -1, & \mathrm{Otherwise} \end{array}\right.$

**Table 4:** BLC and MIR variants.

Variant	Transfer Function and Binarization Rule
BCL [49]	V4-Elitist
MIR [36]	V4-Complement

**Table 5:** Configuration details from SCP instances employed in this work.

Instance Set	m	n	Cost Range	Density (%)
4	200	1000	[1,100]	2
5	200	2000	[1,100]	2
6	200	1000	[1,100]	5
A	300	3000	[1,100]	2
B	300	3000	[1,100]	5
C	400	4000	[1,100]	2
D	400	4000	[1,100]	5

**Table 6:** Parameters’ settings.

Parameter	Value
Independent runs	31
Number of populations	40
Number of iterations	1000
parameter pa of CS	0.25
parameter α of CS	1
parameter β of CS	1.5
parameter Vel. max. of PSO	6
parameter Weight. max. of PSO	0.9
parameter Weight. min. of PSO	0.2
parameter c1 of PSO	2
parameter c2 of PSO	2
parameter *a* of SCA	2
parameter *a* of GWO	decreases linearly from 2 to 0
parameter *a* of WOA	decreases linearly from 2 to 0
parameter *b* of WOA	1
parameter α of machine learning techniques	0.1
parameter γ of machine learning techniques	0.4
parameter *N* of backward Q-learning	10
parameter ϵ of Multi-armed Bandit	0.1

## Data Availability

Data sharing is not applicable to this article.

## Appendix A. Tables of Experimental Results

**Table A1:** Comparison of the CS metaheuristics.

		BCL			MIR			BQSA_40a_pl0			BQSA_40a_pl4			BQSA_80a_pl0			BQSA_80a_pl4		
Inst.	Opt.	Best	Avg	RPD	Best	Avg	RPD	Best	Avg	RPD	Best	Avg	RPD	Best	Avg	RPD	Best	Avg	RPD
41	429	430.0	433.9	0.23	570.0	602.55	32.87	430.0	432.61	0.23	430.0	433.0	0.23	430.0	433.32	0.23	430.0	432.81	0.23
42	512	513.0	521.0	0.2	959.0	1025.65	87.3	513.0	523.74	0.2	516.0	525.61	0.78	514.0	525.26	0.39	517.0	525.71	0.98
43	516	516.0	519.65	0.0	1052.0	1108.58	103.88	516.0	524.9	0.0	516.0	524.84	0.0	516.0	524.58	0.0	516.0	524.87	0.0
44	494	494.0	501.61	0.0	814.0	882.35	64.78	495.0	504.74	0.2	494.0	505.61	0.0	495.0	503.45	0.2	495.0	504.48	0.2
45	512	514.0	517.29	0.39	919.0	1035.81	79.49	514.0	520.03	0.39	515.0	521.03	0.59	517.0	520.94	0.98	514.0	521.74	0.39
46	560	561.0	565.87	0.18	1050.0	1257.0	87.5	561.0	565.68	0.18	560.0	565.52	0.0	562.0	566.68	0.36	561.0	566.45	0.18
47	430	430.0	432.94	0.0	704.0	765.61	63.72	431.0	435.0	0.23	432.0	435.55	0.47	431.0	434.97	0.23	434.0	435.94	0.93
48	492	493.0	496.06	0.2	977.0	1061.42	98.58	493.0	497.87	0.2	493.0	498.23	0.2	493.0	496.84	0.2	493.0	497.19	0.2
49	641	645.0	655.97	0.62	1363.0	1456.97	112.64	647.0	660.35	0.94	653.0	665.32	1.87	647.0	662.19	0.94	652.0	665.1	1.72
410	514	514.0	516.19	0.0	902.0	980.0	75.49	514.0	518.42	0.0	515.0	519.77	0.19	514.0	519.55	0.0	516.0	519.29	0.39
51	253	253.0	258.71	0.0	496.0	534.74	96.05	253.0	258.55	0.0	256.0	261.68	1.19	254.0	259.03	0.4	255.0	260.26	0.79
52	302	305.0	314.06	0.99	749.0	818.42	148.01	312.0	319.77	3.31	310.0	320.84	2.65	314.0	319.97	3.97	309.0	320.97	2.32
53	226	228.0	228.45	0.88	453.0	480.87	100.44	229.0	229.77	1.33	229.0	230.06	1.33	228.0	229.77	0.88	229.0	230.03	1.33
54	242	242.0	243.13	0.0	457.0	503.42	88.84	243.0	246.52	0.41	244.0	247.03	0.83	242.0	246.61	0.0	244.0	248.0	0.83
55	211	212.0	212.77	0.47	331.0	360.35	56.87	211.0	213.23	0.0	211.0	213.29	0.0	211.0	213.61	0.0	211.0	213.45	0.0
56	213	213.0	218.29	0.0	434.0	456.77	103.76	213.0	217.39	0.0	213.0	218.32	0.0	213.0	217.42	0.0	213.0	217.52	0.0
57	293	293.0	298.71	0.0	572.0	616.55	95.22	293.0	301.55	0.0	295.0	301.35	0.68	295.0	300.32	0.68	294.0	300.74	0.34
58	288	288.0	291.23	0.0	590.0	647.94	104.86	288.0	291.55	0.0	288.0	291.45	0.0	288.0	290.61	0.0	288.0	291.68	0.0
59	279	279.0	281.77	0.0	584.0	640.52	109.32	280.0	281.77	0.36	280.0	282.61	0.36	280.0	281.74	0.36	280.0	283.06	0.36
510	265	265.0	268.06	0.0	521.0	569.06	96.6	265.0	271.0	0.0	265.0	270.77	0.0	265.0	269.65	0.0	265.0	270.74	0.0
61	138	138.0	143.61	0.0	604.0	679.13	337.68	138.0	142.45	0.0	138.0	142.87	0.0	140.0	141.97	1.45	138.0	143.06	0.0
62	146	147.0	150.32	0.68	882.0	1012.71	504.11	147.0	150.29	0.68	146.0	150.74	0.0	146.0	149.68	0.0	146.0	150.35	0.0
63	145	145.0	148.13	0.0	848.0	960.52	484.83	145.0	148.13	0.0	145.0	147.87	0.0	145.0	147.71	0.0	145.0	147.55	0.0
64	131	131.0	133.06	0.0	520.0	597.16	296.95	131.0	132.84	0.0	131.0	132.65	0.0	131.0	132.55	0.0	131.0	132.61	0.0
65	161	161.0	167.94	0.0	922.0	1014.0	472.67	161.0	168.39	0.0	161.0	168.71	0.0	161.0	165.71	0.0	161.0	168.61	0.0
a1	253	256.0	257.35	1.19	1198.0	1260.03	373.52	256.0	259.23	1.19	257.0	259.1	1.58	255.0	259.06	0.79	257.0	260.26	1.58
a2	252	254.0	259.03	0.79	1099.0	1164.74	336.11	255.0	260.0	1.19	256.0	261.39	1.59	254.0	260.48	0.79	255.0	260.35	1.19
a3	232	234.0	238.29	0.86	975.0	1095.61	320.26	236.0	240.32	1.72	236.0	240.81	1.72	234.0	240.39	0.86	235.0	240.58	1.29
a4	234	235.0	236.84	0.43	1002.0	1076.94	328.21	235.0	239.0	0.43	236.0	239.39	0.85	235.0	237.94	0.43	237.0	239.81	1.28
a5	236	236.0	237.68	0.0	1032.0	1095.71	337.29	238.0	240.1	0.85	237.0	240.19	0.42	237.0	240.77	0.42	238.0	240.77	0.85
b1	69	69.0	70.77	0.0	1291.0	1380.97	1771.01	69.0	69.81	0.0	69.0	70.03	0.0	69.0	69.87	0.0	69.0	70.0	0.0
b2	76	76.0	77.26	0.0	1294.0	1382.35	1602.63	76.0	76.45	0.0	76.0	76.42	0.0	76.0	76.35	0.0	76.0	76.61	0.0
b3	80	80.0	81.16	0.0	1691.0	1761.71	2013.75	80.0	81.06	0.0	80.0	81.23	0.0	81.0	81.13	1.25	80.0	81.0	0.0
b4	79	79.0	81.81	0.0	1492.0	1582.26	1788.61	79.0	80.58	0.0	79.0	80.61	0.0	79.0	80.35	0.0	79.0	81.03	0.0
b5	72	72.0	72.84	0.0	1314.0	1401.48	1725.0	72.0	72.81	0.0	72.0	72.65	0.0	72.0	72.58	0.0	72.0	72.74	0.0
c1	227	229.0	232.26	0.88	1420.0	1545.45	525.55	230.0	234.94	1.32	232.0	235.81	2.2	232.0	235.32	2.2	229.0	235.52	0.88
c2	219	221.0	224.26	0.91	1644.0	1744.87	650.68	221.0	226.58	0.91	224.0	229.16	2.28	221.0	225.71	0.91	222.0	227.23	1.37
c3	243	244.0	250.42	0.41	1921.0	2054.77	690.53	244.0	249.65	0.41	244.0	249.77	0.41	245.0	249.1	0.82	245.0	249.52	0.82
c4	219	220.0	226.87	0.46	1531.0	1689.42	599.09	220.0	225.84	0.46	221.0	225.9	0.91	220.0	225.55	0.46	221.0	225.32	0.91
c5	215	215.0	217.71	0.0	1546.0	1617.87	619.07	218.0	220.0	1.4	216.0	220.48	0.47	215.0	220.29	0.0	218.0	220.74	1.4
d1	60	60.0	61.81	0.0	1912.0	2045.97	3086.67	60.0	61.68	0.0	60.0	62.55	0.0	60.0	61.65	0.0	60.0	61.58	0.0
d2	66	66.0	67.58	0.0	2048.0	2296.9	3003.03	66.0	67.32	0.0	67.0	67.52	1.52	66.0	67.26	0.0	67.0	67.32	1.52
d3	72	72.0	75.13	0.0	2418.0	2532.19	3258.33	72.0	74.42	0.0	73.0	74.65	1.39	73.0	74.55	1.39	73.0	74.9	1.39
d4	62	62.0	63.9	0.0	1985.0	2058.32	3101.61	62.0	62.16	0.0	62.0	62.39	0.0	62.0	62.16	0.0	62.0	62.19	0.0
d5	61	61.0	61.94	0.0	1869.0	2030.77	2963.93	61.0	61.97	0.0	61.0	62.0	0.0	61.0	62.1	0.0	61.0	62.1	0.0
		254.47	258.08	0.24	1087.89	1175.25	733.27	254.96	259.12	0.41	255.42	259.71	0.59	255.09	259.04	0.48	255.4	259.6	0.57
		SA_40a_pl0			SA_40a_pl4			SA_80a_pl0			SA_80a_pl4			MAB_40a_pl0			MAB_40a_pl4		
Inst.	Opt.	Best	Avg	RPD	Best	Avg	RPD	Best	Avg	RPD	Best	Avg	RPD	Best	Avg	RPD	Best	Avg	RPD
41	429	430.0	433.16	0.23	431.0	433.13	0.47	430.0	433.77	0.23	431.0	434.03	0.47	430.0	434.16	0.23	430.0	433.06	0.23
42	512	513.0	525.32	0.2	514.0	527.81	0.39	514.0	526.52	0.39	519.0	527.26	1.37	513.0	524.19	0.2	514.0	528.77	0.39
43	516	519.0	524.19	0.58	520.0	526.06	0.78	520.0	523.77	0.78	520.0	526.52	0.78	516.0	525.13	0.0	516.0	525.42	0.0
44	494	495.0	502.13	0.2	494.0	505.65	0.0	495.0	504.23	0.2	497.0	506.13	0.61	494.0	506.23	0.0	495.0	503.61	0.2
45	512	514.0	522.13	0.39	512.0	522.26	0.0	514.0	520.71	0.39	519.0	525.52	1.37	514.0	522.71	0.39	518.0	520.87	1.17
46	560	560.0	566.45	0.0	560.0	570.55	0.0	561.0	567.0	0.18	562.0	566.65	0.36	560.0	567.94	0.0	561.0	567.13	0.18
47	430	431.0	435.45	0.23	432.0	435.68	0.47	430.0	435.23	0.0	432.0	435.9	0.47	431.0	434.97	0.23	431.0	435.03	0.23
48	492	493.0	498.03	0.2	493.0	498.9	0.2	493.0	497.97	0.2	493.0	498.65	0.2	493.0	497.68	0.2	493.0	498.61	0.2
49	641	648.0	664.32	1.09	653.0	662.94	1.87	650.0	666.1	1.4	652.0	667.81	1.72	644.0	668.23	0.47	653.0	662.58	1.87
410	514	514.0	519.68	0.0	514.0	519.55	0.0	514.0	519.29	0.0	516.0	520.94	0.39	514.0	520.26	0.0	515.0	520.0	0.19
51	253	254.0	260.71	0.4	254.0	261.61	0.4	255.0	260.35	0.79	257.0	261.87	1.58	253.0	259.0	0.0	253.0	260.03	0.0
52	302	310.0	320.06	2.65	316.0	323.03	4.64	312.0	320.42	3.31	315.0	320.71	4.3	311.0	322.48	2.98	313.0	320.87	3.64
53	226	228.0	230.13	0.88	228.0	230.19	0.88	228.0	229.77	0.88	229.0	230.1	1.33	229.0	230.45	1.33	229.0	229.74	1.33
54	242	242.0	247.06	0.0	242.0	246.77	0.0	244.0	247.29	0.83	244.0	247.42	0.83	242.0	247.26	0.0	242.0	246.71	0.0
55	211	211.0	213.13	0.0	211.0	213.58	0.0	211.0	213.26	0.0	212.0	213.94	0.47	211.0	214.45	0.0	211.0	213.06	0.0
56	213	213.0	218.42	0.0	213.0	217.48	0.0	213.0	217.29	0.0	213.0	218.77	0.0	213.0	218.39	0.0	213.0	219.58	0.0
57	293	294.0	300.9	0.34	295.0	302.87	0.68	295.0	300.16	0.68	296.0	301.87	1.02	294.0	302.19	0.34	298.0	300.74	1.71
58	288	289.0	291.9	0.35	288.0	292.0	0.0	288.0	291.23	0.0	288.0	291.48	0.0	288.0	290.81	0.0	288.0	291.13	0.0
59	279	280.0	282.39	0.36	279.0	282.39	0.0	280.0	282.1	0.36	280.0	283.19	0.36	280.0	282.39	0.36	279.0	282.32	0.0
510	265	266.0	271.52	0.38	265.0	271.06	0.0	266.0	269.55	0.38	265.0	271.61	0.0	265.0	270.9	0.0	265.0	271.61	0.0
61	138	140.0	142.61	1.45	140.0	142.81	1.45	141.0	142.68	2.17	140.0	142.61	1.45	140.0	142.74	1.45	141.0	142.94	2.17
62	146	146.0	149.97	0.0	147.0	151.87	0.68	146.0	149.97	0.0	146.0	150.06	0.0	146.0	150.13	0.0	147.0	151.16	0.68
63	145	145.0	147.97	0.0	145.0	148.35	0.0	145.0	148.29	0.0	145.0	147.81	0.0	145.0	148.0	0.0	145.0	148.29	0.0
64	131	131.0	132.52	0.0	131.0	133.74	0.0	131.0	132.94	0.0	131.0	132.68	0.0	131.0	132.77	0.0	131.0	133.35	0.0
65	161	161.0	167.97	0.0	161.0	170.9	0.0	161.0	166.84	0.0	161.0	167.06	0.0	161.0	166.23	0.0	161.0	170.87	0.0
a1	253	256.0	259.87	1.19	255.0	259.65	0.79	256.0	259.74	1.19	260.0	262.61	2.77	256.0	261.26	1.19	257.0	259.61	1.58
a2	252	255.0	260.52	1.19	254.0	260.32	0.79	254.0	260.68	0.79	257.0	261.61	1.98	255.0	262.16	1.19	255.0	261.03	1.19
a3	232	237.0	241.29	2.16	236.0	241.0	1.72	236.0	240.87	1.72	236.0	241.42	1.72	236.0	241.77	1.72	235.0	240.94	1.29
a4	234	235.0	238.81	0.43	235.0	240.1	0.43	235.0	238.81	0.43	238.0	241.03	1.71	235.0	240.03	0.43	236.0	239.1	0.85
a5	236	237.0	241.0	0.42	237.0	240.68	0.42	238.0	240.45	0.85	238.0	241.84	0.85	237.0	242.1	0.42	238.0	240.84	0.85
b1	69	69.0	70.0	0.0	69.0	70.61	0.0	69.0	69.94	0.0	69.0	69.45	0.0	69.0	69.84	0.0	69.0	70.48	0.0
b2	76	76.0	76.52	0.0	76.0	77.29	0.0	76.0	76.52	0.0	76.0	76.52	0.0	76.0	76.29	0.0	76.0	76.97	0.0
b3	80	81.0	81.19	1.25	81.0	81.58	1.25	80.0	81.06	0.0	80.0	81.03	0.0	80.0	81.16	0.0	80.0	81.23	0.0
b4	79	79.0	80.48	0.0	79.0	81.0	0.0	79.0	81.0	0.0	79.0	80.87	0.0	79.0	80.94	0.0	79.0	80.55	0.0
b5	72	72.0	72.71	0.0	72.0	72.94	0.0	72.0	72.68	0.0	72.0	72.94	0.0	72.0	72.74	0.0	72.0	72.77	0.0
c1	227	231.0	236.19	1.76	229.0	236.52	0.88	230.0	235.32	1.32	233.0	237.26	2.64	230.0	236.52	1.32	232.0	237.61	2.2
c2	219	221.0	227.23	0.91	223.0	229.26	1.83	221.0	226.26	0.91	224.0	228.77	2.28	221.0	227.77	0.91	223.0	228.58	1.83
c3	243	245.0	249.94	0.82	246.0	251.77	1.23	245.0	249.45	0.82	245.0	249.71	0.82	245.0	250.32	0.82	245.0	251.65	0.82
c4	219	221.0	225.13	0.91	220.0	226.77	0.46	221.0	225.06	0.91	222.0	226.81	1.37	221.0	227.61	0.91	220.0	226.26	0.46
c5	215	216.0	220.55	0.47	217.0	220.9	0.93	216.0	220.71	0.47	217.0	221.84	0.93	216.0	222.52	0.47	217.0	220.55	0.93
d1	60	60.0	62.13	0.0	61.0	63.06	1.67	60.0	61.52	0.0	60.0	61.71	0.0	60.0	62.0	0.0	60.0	62.45	0.0
d2	66	66.0	67.39	0.0	67.0	68.1	1.52	66.0	67.23	0.0	67.0	67.45	1.52	67.0	67.58	1.52	66.0	67.77	0.0
d3	72	72.0	74.35	0.0	73.0	75.45	1.39	73.0	74.39	1.39	72.0	74.39	0.0	72.0	74.32	0.0	74.0	75.45	2.78
d4	62	62.0	62.13	0.0	62.0	62.61	0.0	62.0	62.16	0.0	62.0	62.16	0.0	62.0	62.48	0.0	62.0	62.23	0.0
d5	61	61.0	62.19	0.0	61.0	63.19	0.0	61.0	62.03	0.0	61.0	61.97	0.0	61.0	62.29	0.0	61.0	62.84	0.0
		255.09	259.51	0.48	255.36	260.31	0.63	255.27	259.39	0.53	256.24	260.27	0.84	254.84	260.03	0.42	255.53	259.92	0.64
		MAB_80a_pl0			MAB_80a_pl4			QL_40a_pl0			QL_40a_pl4			QL_80a_pl0			QL_80a_pl4		
Inst.	Opt.	Best	Avg	RPD	Best	Avg	RPD	Best	Avg	RPD	Best	Avg	RPD	Best	Avg	RPD	Best	Avg	RPD
41	429	430.0	432.97	0.23	430.0	433.32	0.23	430.0	432.16	0.23	430.0	433.16	0.23	430.0	432.68	0.23	430.0	432.87	0.23
42	512	513.0	526.0	0.2	514.0	523.13	0.39	514.0	522.71	0.39	513.0	525.06	0.2	512.0	523.87	0.0	516.0	524.48	0.78
43	516	516.0	525.0	0.0	517.0	523.84	0.19	516.0	524.55	0.0	519.0	524.58	0.58	517.0	521.97	0.19	519.0	524.19	0.58
44	494	495.0	505.23	0.2	495.0	503.84	0.2	495.0	502.68	0.2	496.0	503.74	0.4	494.0	501.55	0.0	495.0	505.29	0.2
45	512	515.0	524.06	0.59	514.0	520.23	0.39	515.0	519.87	0.59	514.0	521.0	0.39	516.0	520.0	0.78	515.0	521.48	0.59
46	560	561.0	566.29	0.18	561.0	565.65	0.18	560.0	564.52	0.0	561.0	564.81	0.18	561.0	564.52	0.18	561.0	565.81	0.18
47	430	431.0	435.84	0.23	430.0	435.0	0.0	432.0	434.48	0.47	431.0	435.19	0.23	432.0	435.61	0.47	432.0	435.77	0.47
48	492	493.0	497.74	0.2	493.0	496.87	0.2	493.0	496.58	0.2	493.0	496.42	0.2	493.0	495.77	0.2	493.0	496.68	0.2
49	641	645.0	664.61	0.62	652.0	664.48	1.72	644.0	660.29	0.47	652.0	662.61	1.72	652.0	664.81	1.72	652.0	661.74	1.72
410	514	514.0	520.03	0.0	515.0	518.77	0.19	514.0	517.97	0.0	514.0	519.16	0.0	514.0	518.32	0.0	515.0	519.48	0.19
51	253	254.0	260.74	0.4	254.0	259.32	0.4	254.0	258.61	0.4	254.0	259.68	0.4	254.0	258.77	0.4	256.0	259.68	1.19
52	302	309.0	320.71	2.32	312.0	319.16	3.31	312.0	319.71	3.31	313.0	321.19	3.64	311.0	320.61	2.98	314.0	320.84	3.97
53	226	229.0	230.03	1.33	229.0	229.68	1.33	229.0	229.55	1.33	228.0	229.74	0.88	228.0	229.94	0.88	228.0	229.97	0.88
54	242	242.0	246.68	0.0	244.0	247.26	0.83	243.0	246.48	0.41	244.0	246.97	0.83	242.0	245.87	0.0	245.0	247.71	1.24
55	211	211.0	214.03	0.0	211.0	213.13	0.0	211.0	212.81	0.0	211.0	213.32	0.0	211.0	213.26	0.0	211.0	212.65	0.0
56	213	213.0	217.87	0.0	215.0	218.23	0.94	213.0	217.42	0.0	213.0	218.52	0.0	213.0	217.74	0.0	213.0	217.13	0.0
57	293	296.0	301.32	1.02	296.0	301.65	1.02	294.0	300.39	0.34	294.0	301.1	0.34	294.0	301.26	0.34	294.0	300.65	0.34
58	288	288.0	290.39	0.0	288.0	290.32	0.0	288.0	291.06	0.0	288.0	291.94	0.0	288.0	290.45	0.0	288.0	290.97	0.0
59	279	280.0	281.42	0.36	280.0	281.87	0.36	280.0	281.35	0.36	280.0	281.48	0.36	280.0	281.42	0.36	279.0	281.48	0.0
510	265	265.0	271.03	0.0	265.0	269.65	0.0	265.0	270.26	0.0	266.0	270.13	0.38	265.0	269.35	0.0	265.0	269.35	0.0
61	138	140.0	141.74	1.45	141.0	142.97	2.17	138.0	141.71	0.0	140.0	142.94	1.45	138.0	141.81	0.0	138.0	142.71	0.0
62	146	146.0	148.48	0.0	146.0	149.26	0.0	146.0	149.13	0.0	147.0	150.58	0.68	146.0	149.61	0.0	146.0	148.58	0.0
63	145	145.0	147.65	0.0	145.0	147.42	0.0	145.0	147.87	0.0	145.0	147.94	0.0	145.0	147.42	0.0	145.0	147.77	0.0
64	131	131.0	131.87	0.0	131.0	132.52	0.0	131.0	132.68	0.0	131.0	133.13	0.0	131.0	132.52	0.0	131.0	132.32	0.0
65	161	161.0	164.29	0.0	161.0	166.74	0.0	161.0	166.84	0.0	161.0	168.87	0.0	161.0	164.61	0.0	161.0	168.45	0.0
a1	253	257.0	260.97	1.58	255.0	260.0	0.79	255.0	259.55	0.79	256.0	259.39	1.19	257.0	259.58	1.58	257.0	259.81	1.58
a2	252	254.0	261.1	0.79	255.0	261.29	1.19	254.0	260.58	0.79	256.0	261.35	1.59	256.0	260.65	1.59	254.0	260.45	0.79
a3	232	236.0	240.55	1.72	235.0	241.32	1.29	234.0	240.1	0.86	235.0	240.55	1.29	236.0	240.06	1.72	236.0	240.97	1.72
a4	234	236.0	240.35	0.85	236.0	240.19	0.85	235.0	239.29	0.43	236.0	239.58	0.85	235.0	238.71	0.43	234.0	239.84	0.0
a5	236	238.0	240.52	0.85	238.0	240.68	0.85	237.0	239.94	0.42	237.0	240.9	0.42	238.0	239.87	0.85	238.0	241.03	0.85
b1	69	69.0	69.52	0.0	69.0	69.81	0.0	69.0	69.81	0.0	69.0	69.77	0.0	69.0	69.42	0.0	69.0	69.81	0.0
b2	76	76.0	76.32	0.0	76.0	76.32	0.0	76.0	76.48	0.0	76.0	76.61	0.0	76.0	76.16	0.0	76.0	76.29	0.0
b3	80	80.0	80.94	0.0	80.0	80.97	0.0	81.0	81.06	1.25	80.0	81.16	0.0	80.0	80.97	0.0	80.0	80.81	0.0
b4	79	79.0	80.48	0.0	79.0	80.55	0.0	79.0	80.26	0.0	79.0	81.0	0.0	79.0	80.03	0.0	79.0	81.23	0.0
b5	72	72.0	72.45	0.0	72.0	72.74	0.0	72.0	72.55	0.0	72.0	72.77	0.0	72.0	72.58	0.0	72.0	72.55	0.0
c1	227	230.0	235.74	1.32	233.0	235.74	2.64	230.0	234.68	1.32	232.0	236.84	2.2	230.0	234.65	1.32	232.0	235.68	2.2
c2	219	220.0	227.26	0.46	223.0	227.0	1.83	221.0	226.1	0.91	221.0	227.52	0.91	221.0	226.1	0.91	222.0	226.77	1.37
c3	243	245.0	249.35	0.82	245.0	250.55	0.82	245.0	248.84	0.82	245.0	250.06	0.82	244.0	248.81	0.41	244.0	249.32	0.41
c4	219	222.0	226.06	1.37	221.0	226.0	0.91	220.0	225.52	0.46	220.0	224.81	0.46	219.0	224.87	0.0	220.0	226.06	0.46
c5	215	217.0	221.26	0.93	217.0	220.94	0.93	217.0	219.94	0.93	217.0	220.71	0.93	217.0	220.42	0.93	218.0	220.9	1.4
d1	60	60.0	61.52	0.0	60.0	62.0	0.0	60.0	61.55	0.0	60.0	62.23	0.0	60.0	61.52	0.0	60.0	61.81	0.0
d2	66	66.0	67.19	0.0	66.0	67.16	0.0	67.0	67.39	1.52	67.0	67.55	1.52	66.0	67.26	0.0	66.0	67.13	0.0
d3	72	73.0	74.29	1.39	73.0	74.16	1.39	72.0	74.03	0.0	73.0	74.84	1.39	72.0	74.06	0.0	73.0	74.52	1.39
d4	62	62.0	62.16	0.0	62.0	62.13	0.0	62.0	62.16	0.0	62.0	62.26	0.0	62.0	62.1	0.0	62.0	62.26	0.0
d5	61	61.0	61.97	0.0	61.0	61.71	0.0	61.0	62.0	0.0	61.0	62.29	0.0	61.0	61.55	0.0	61.0	61.81	0.0
		255.02	259.47	0.48	255.44	259.23	0.61	254.89	258.74	0.43	255.38	259.45	0.59	255.07	258.74	0.41	255.44	259.27	0.55

**Table A2:** Comparison of the PSO metaheuristics.

		BCL			MIR_40a_pl0			BQSA_40a_pl0			BQSA_40a_pl4			BQSA_80a_pl0			BQSA_80a_pl4		
Inst.	Opt.	Best	Avg	RPD	Best	Avg	RPD	Best	Avg	RPD	Best	Avg	RPD	Best	Avg	RPD	Best	Avg	RPD
41	429	430.0	434.26	0.23	497.0	538.1	15.85	431.0	435.52	0.47	430.0	435.97	0.23	430.0	434.81	0.23	430.0	434.94	0.23
42	512	513.0	527.35	0.2	780.0	847.65	52.34	518.0	535.06	1.17	528.0	541.16	3.12	516.0	530.1	0.78	519.0	533.06	1.37
43	516	516.0	521.68	0.0	829.0	910.19	60.66	524.0	534.77	1.55	517.0	535.0	0.19	520.0	529.13	0.78	518.0	530.13	0.39
44	494	495.0	506.23	0.2	676.0	740.39	36.84	501.0	511.74	1.42	500.0	514.26	1.21	497.0	508.19	0.61	497.0	510.68	0.61
45	512	514.0	518.9	0.39	781.0	864.1	52.54	520.0	530.71	1.56	518.0	533.94	1.17	521.0	529.39	1.76	518.0	526.65	1.17
46	560	562.0	568.61	0.36	946.0	1036.1	68.93	564.0	572.71	0.71	563.0	575.48	0.54	564.0	568.65	0.71	564.0	569.77	0.71
47	430	432.0	435.52	0.47	596.0	656.94	38.6	433.0	440.23	0.7	433.0	441.84	0.7	430.0	438.13	0.0	433.0	439.52	0.7
48	492	493.0	497.87	0.2	799.0	873.55	62.4	493.0	503.29	0.2	493.0	503.19	0.2	496.0	500.19	0.81	493.0	501.16	0.2
49	641	652.0	666.68	1.72	1054.0	1180.06	64.43	660.0	679.03	2.96	652.0	678.06	1.72	651.0	674.74	1.56	652.0	674.06	1.72
410	514	514.0	520.1	0.0	774.0	825.48	50.58	514.0	525.94	0.0	516.0	523.81	0.39	516.0	521.68	0.39	516.0	522.61	0.39
51	253	254.0	259.0	0.4	395.0	438.52	56.13	257.0	265.65	1.58	257.0	266.03	1.58	254.0	263.58	0.4	257.0	262.29	1.58
52	302	308.0	316.65	1.99	586.0	641.94	94.04	313.0	324.35	3.64	317.0	327.9	4.97	310.0	323.16	2.65	313.0	323.06	3.64
53	226	228.0	229.19	0.88	361.0	396.77	59.73	229.0	231.16	1.33	229.0	232.32	1.33	229.0	230.65	1.33	229.0	230.65	1.33
54	242	243.0	247.1	0.41	378.0	412.42	56.2	245.0	250.45	1.24	246.0	249.9	1.65	245.0	248.74	1.24	245.0	249.58	1.24
55	211	211.0	214.06	0.0	284.0	307.13	34.6	212.0	216.9	0.47	213.0	216.81	0.95	212.0	214.68	0.47	211.0	214.26	0.0
56	213	213.0	218.87	0.0	323.0	375.06	51.64	217.0	225.13	1.88	215.0	224.48	0.94	215.0	220.1	0.94	214.0	220.48	0.47
57	293	294.0	299.71	0.34	458.0	510.77	56.31	293.0	306.23	0.0	298.0	306.65	1.71	295.0	302.77	0.68	295.0	304.06	0.68
58	288	288.0	292.74	0.0	489.0	525.77	69.79	290.0	296.13	0.69	291.0	297.26	1.04	289.0	293.23	0.35	289.0	293.48	0.35
59	279	280.0	284.71	0.36	473.0	527.48	69.53	280.0	286.55	0.36	280.0	287.03	0.36	280.0	283.9	0.36	280.0	283.87	0.36
510	265	265.0	270.03	0.0	414.0	473.48	56.23	266.0	273.81	0.38	265.0	274.0	0.0	266.0	272.1	0.38	266.0	272.87	0.38
61	138	138.0	143.81	0.0	419.0	492.68	203.62	142.0	145.39	2.9	142.0	145.42	2.9	140.0	143.71	1.45	140.0	144.1	1.45
62	146	146.0	149.58	0.0	649.0	743.42	344.52	147.0	153.61	0.68	149.0	153.9	2.05	147.0	151.84	0.68	146.0	151.65	0.0
63	145	145.0	148.26	0.0	588.0	684.97	305.52	145.0	151.0	0.0	147.0	151.84	1.38	145.0	148.48	0.0	147.0	149.29	1.38
64	131	131.0	133.0	0.0	389.0	432.06	196.95	131.0	134.87	0.0	131.0	134.61	0.0	132.0	133.61	0.76	131.0	133.48	0.0
65	161	161.0	168.0	0.0	667.0	733.55	314.29	165.0	174.55	2.48	164.0	173.77	1.86	162.0	170.0	0.62	162.0	171.52	0.62
a1	253	255.0	258.29	0.79	863.0	960.68	241.11	257.0	264.35	1.58	259.0	265.81	2.37	257.0	261.55	1.58	258.0	262.45	1.98
a2	252	254.0	260.52	0.79	779.0	901.16	209.13	260.0	268.84	3.17	262.0	269.26	3.97	258.0	263.87	2.38	256.0	263.39	1.59
a3	232	235.0	240.52	1.29	776.0	842.23	234.48	238.0	245.0	2.59	239.0	247.32	3.02	238.0	242.13	2.59	237.0	242.81	2.16
a4	234	235.0	240.19	0.43	777.0	830.29	232.05	237.0	247.65	1.28	241.0	248.52	2.99	237.0	241.97	1.28	238.0	243.16	1.71
a5	236	236.0	239.1	0.0	777.0	843.13	229.24	240.0	245.94	1.69	239.0	246.16	1.27	239.0	242.61	1.27	240.0	243.52	1.69
b1	69	69.0	70.58	0.0	881.0	1029.52	1176.81	69.0	71.94	0.0	69.0	72.81	0.0	69.0	70.1	0.0	69.0	70.55	0.0
b2	76	76.0	77.87	0.0	912.0	1031.19	1100.0	76.0	81.0	0.0	76.0	81.48	0.0	76.0	76.68	0.0	76.0	77.9	0.0
b3	80	80.0	81.19	0.0	1255.0	1341.45	1468.75	80.0	82.97	0.0	81.0	83.16	1.25	80.0	81.23	0.0	81.0	81.84	1.25
b4	79	79.0	81.23	0.0	1073.0	1210.65	1258.23	80.0	83.1	1.27	82.0	84.74	3.8	79.0	81.39	0.0	80.0	82.1	1.27
b5	72	72.0	72.74	0.0	914.0	1057.52	1169.44	72.0	74.61	0.0	72.0	75.13	0.0	72.0	72.9	0.0	72.0	73.32	0.0
c1	227	229.0	233.87	0.88	1099.0	1193.0	384.14	235.0	241.81	3.52	235.0	243.35	3.52	232.0	236.9	2.2	232.0	238.68	2.2
c2	219	220.0	225.52	0.46	1221.0	1363.06	457.53	226.0	234.58	3.2	226.0	237.06	3.2	225.0	230.45	2.74	226.0	231.0	3.2
c3	243	244.0	251.19	0.41	1466.0	1589.32	503.29	246.0	258.26	1.23	246.0	260.71	1.23	247.0	251.97	1.65	244.0	252.84	0.41
c4	219	220.0	228.9	0.46	1216.0	1313.94	455.25	224.0	235.06	2.28	226.0	236.26	3.2	224.0	229.19	2.28	223.0	229.26	1.83
c5	215	215.0	219.26	0.0	1169.0	1253.87	443.72	222.0	227.48	3.26	221.0	229.35	2.79	219.0	222.84	1.86	219.0	223.65	1.86
d1	60	60.0	61.68	0.0	1473.0	1574.9	2355.0	61.0	64.39	1.67	61.0	65.19	1.67	61.0	62.58	1.67	61.0	63.1	1.67
d2	66	66.0	67.35	0.0	1662.0	1821.39	2418.18	66.0	69.06	0.0	67.0	69.52	1.52	66.0	67.68	0.0	66.0	67.81	0.0
d3	72	73.0	74.77	1.39	1834.0	1998.06	2447.22	73.0	77.06	1.39	73.0	78.74	1.39	73.0	75.13	1.39	73.0	76.0	1.39
d4	62	62.0	63.39	0.0	1486.0	1614.26	2296.77	62.0	65.48	0.0	62.0	65.03	0.0	62.0	62.9	0.0	62.0	62.97	0.0
d5	61	61.0	61.81	0.0	1466.0	1606.23	2303.28	61.0	64.77	0.0	62.0	66.65	1.64	61.0	62.45	0.0	61.0	63.0	0.0
		254.82	259.6	0.33	837.87	923.21	530.13	257.22	264.63	1.26	257.62	265.58	1.58	256.38	261.6	0.95	256.42	262.15	1.0
		SA_40a_pl0			SA_40a_pl4			SA_80a_pl0			SA_80a_pl4			MAB_40a_pl0			MAB_40a_pl4		
Inst.	Opt.	Best	Avg	RPD	Best	Avg	RPD	Best	Avg	RPD	Best	Avg	RPD	Best	Avg	RPD	Best	Avg	RPD
41	429	430.0	437.16	0.23	430.0	436.58	0.23	431.0	435.81	0.47	431.0	435.74	0.47	431.0	436.23	0.47	430.0	435.68	0.23
42	512	522.0	539.03	1.95	522.0	541.19	1.95	518.0	532.84	1.17	521.0	536.87	1.76	513.0	536.52	0.2	517.0	537.1	0.98
43	516	520.0	533.1	0.78	522.0	539.58	1.16	520.0	530.77	0.78	522.0	531.48	1.16	521.0	534.65	0.97	524.0	534.65	1.55
44	494	500.0	516.13	1.21	504.0	517.06	2.02	502.0	510.0	1.62	501.0	513.32	1.42	499.0	511.39	1.01	500.0	514.71	1.21
45	512	520.0	533.48	1.56	521.0	531.94	1.76	518.0	526.29	1.17	522.0	530.77	1.95	521.0	531.71	1.76	520.0	534.03	1.56
46	560	566.0	574.03	1.07	564.0	579.26	0.71	561.0	569.32	0.18	562.0	572.06	0.36	564.0	572.94	0.71	564.0	575.29	0.71
47	430	433.0	440.0	0.7	433.0	442.55	0.7	431.0	438.9	0.23	432.0	439.19	0.47	430.0	442.9	0.0	436.0	442.68	1.4
48	492	493.0	505.9	0.2	497.0	505.29	1.02	493.0	500.0	0.2	494.0	503.03	0.41	493.0	501.97	0.2	496.0	503.45	0.81
49	641	660.0	683.16	2.96	658.0	680.16	2.65	662.0	675.65	3.28	662.0	679.1	3.28	660.0	681.0	2.96	657.0	679.55	2.5
410	514	519.0	526.68	0.97	517.0	527.52	0.58	516.0	523.13	0.39	517.0	523.77	0.58	514.0	523.48	0.0	514.0	526.1	0.0
51	253	256.0	264.32	1.19	260.0	266.26	2.77	257.0	264.23	1.58	257.0	265.0	1.58	255.0	265.42	0.79	259.0	266.16	2.37
52	302	316.0	326.58	4.64	320.0	330.55	5.96	313.0	324.58	3.64	309.0	326.84	2.32	316.0	325.1	4.64	322.0	329.29	6.62
53	226	229.0	232.81	1.33	229.0	232.77	1.33	229.0	230.77	1.33	230.0	232.94	1.77	229.0	232.26	1.33	229.0	232.94	1.33
54	242	245.0	250.9	1.24	244.0	251.39	0.83	244.0	249.03	0.83	246.0	250.42	1.65	244.0	250.71	0.83	246.0	250.55	1.65
55	211	212.0	216.45	0.47	212.0	217.39	0.47	212.0	215.52	0.47	212.0	215.68	0.47	211.0	216.52	0.0	212.0	216.84	0.47
56	213	214.0	224.42	0.47	214.0	225.1	0.47	216.0	221.06	1.41	214.0	222.58	0.47	216.0	225.29	1.41	216.0	224.1	1.41
57	293	299.0	306.45	2.05	299.0	307.55	2.05	296.0	303.97	1.02	300.0	304.61	2.39	298.0	307.45	1.71	303.0	307.74	3.41
58	288	291.0	299.29	1.04	291.0	298.84	1.04	289.0	293.68	0.35	291.0	295.68	1.04	290.0	298.35	0.69	288.0	298.19	0.0
59	279	283.0	289.58	1.43	280.0	287.94	0.36	280.0	284.29	0.36	282.0	285.06	1.08	281.0	287.55	0.72	280.0	288.77	0.36
510	265	268.0	274.87	1.13	266.0	276.26	0.38	266.0	272.06	0.38	266.0	273.45	0.38	266.0	274.23	0.38	268.0	275.87	1.13
61	138	141.0	144.71	2.17	142.0	146.58	2.9	141.0	143.06	2.17	140.0	144.06	1.45	141.0	144.94	2.17	143.0	146.81	3.62
62	146	146.0	154.06	0.0	147.0	156.03	0.68	148.0	151.81	1.37	148.0	152.81	1.37	146.0	154.58	0.0	147.0	156.84	0.68
63	145	145.0	151.06	0.0	147.0	150.77	1.38	146.0	148.39	0.69	145.0	149.29	0.0	148.0	150.65	2.07	148.0	152.45	2.07
64	131	131.0	135.61	0.0	131.0	135.23	0.0	131.0	132.84	0.0	131.0	133.84	0.0	131.0	134.13	0.0	132.0	135.52	0.76
65	161	161.0	175.23	0.0	164.0	176.74	1.86	162.0	169.45	0.62	161.0	171.23	0.0	161.0	173.94	0.0	165.0	176.26	2.48
a1	253	260.0	266.42	2.77	257.0	267.29	1.58	259.0	262.58	2.37	260.0	264.23	2.77	257.0	265.84	1.58	260.0	267.39	2.77
a2	252	258.0	267.74	2.38	259.0	271.39	2.78	258.0	263.74	2.38	258.0	265.77	2.38	258.0	268.74	2.38	259.0	270.29	2.78
a3	232	238.0	246.84	2.59	238.0	247.19	2.59	237.0	241.87	2.16	239.0	243.39	3.02	236.0	245.16	1.72	241.0	249.1	3.88
a4	234	238.0	248.71	1.71	240.0	251.13	2.56	236.0	242.61	0.85	237.0	245.0	1.28	237.0	249.48	1.28	239.0	250.52	2.14
a5	236	239.0	246.52	1.27	240.0	248.81	1.69	237.0	243.13	0.42	238.0	244.19	0.85	240.0	248.65	1.69	240.0	247.94	1.69
b1	69	69.0	73.1	0.0	70.0	74.9	1.45	69.0	69.9	0.0	69.0	70.81	0.0	69.0	72.65	0.0	71.0	75.06	2.9
b2	76	76.0	81.52	0.0	76.0	82.42	0.0	76.0	76.97	0.0	76.0	78.52	0.0	76.0	79.87	0.0	77.0	83.23	1.32
b3	80	80.0	84.19	0.0	80.0	84.77	0.0	80.0	81.48	0.0	81.0	82.35	1.25	80.0	82.74	0.0	81.0	85.65	1.25
b4	79	81.0	84.77	2.53	81.0	85.42	2.53	79.0	81.71	0.0	80.0	82.94	1.27	79.0	83.1	0.0	82.0	86.29	3.8
b5	72	72.0	74.68	0.0	72.0	75.97	0.0	72.0	72.84	0.0	72.0	73.29	0.0	72.0	73.87	0.0	73.0	77.23	1.39
c1	227	235.0	243.52	3.52	234.0	246.48	3.08	231.0	237.84	1.76	234.0	239.55	3.08	234.0	244.1	3.08	239.0	245.77	5.29
c2	219	227.0	236.48	3.65	230.0	239.1	5.02	222.0	229.94	1.37	224.0	232.77	2.28	227.0	239.32	3.65	233.0	243.16	6.39
c3	243	250.0	260.39	2.88	250.0	264.61	2.88	246.0	251.81	1.23	247.0	255.42	1.65	247.0	262.06	1.65	251.0	266.81	3.29
c4	219	226.0	235.84	3.2	231.0	239.55	5.48	222.0	228.65	1.37	223.0	229.81	1.83	222.0	235.84	1.37	228.0	238.48	4.11
c5	215	219.0	227.65	1.86	221.0	231.52	2.79	218.0	222.58	1.4	219.0	224.0	1.86	220.0	230.74	2.33	220.0	230.94	2.33
d1	60	61.0	65.0	1.67	61.0	68.0	1.67	60.0	62.13	0.0	61.0	63.16	1.67	60.0	63.19	0.0	62.0	68.58	3.33
d2	66	66.0	69.48	0.0	67.0	72.29	1.52	67.0	67.58	1.52	67.0	68.06	1.52	66.0	69.13	0.0	68.0	75.32	3.03
d3	72	74.0	78.65	2.78	74.0	79.52	2.78	73.0	74.9	1.39	73.0	75.71	1.39	73.0	77.0	1.39	74.0	81.32	2.78
d4	62	62.0	66.13	0.0	62.0	67.71	0.0	62.0	62.61	0.0	62.0	64.1	0.0	62.0	64.97	0.0	64.0	69.42	3.23
d5	61	61.0	64.84	0.0	62.0	68.45	1.64	61.0	62.58	0.0	61.0	63.06	0.0	61.0	65.26	0.0	63.0	69.84	3.28
		257.6	265.72	1.37	258.2	267.27	1.72	256.6	261.89	0.98	257.27	263.44	1.24	256.78	265.24	1.05	258.69	267.2	2.23
		MAB_80a_pl0			MAB_80a_pl4			QL_40a_pl0			QL_40a_pl4			QL_80a_pl0			QL_80a_pl4		
Inst.	Opt.	Best	Avg	RPD	Best	Avg	RPD	Best	Avg	RPD	Best	Avg	RPD	Best	Avg	RPD	Best	Avg	RPD
41	429	430.0	433.29	0.23	431.0	435.03	0.47	431.0	435.48	0.47	431.0	435.9	0.47	430.0	432.71	0.23	431.0	434.0	0.47
42	512	515.0	530.16	0.59	522.0	536.32	1.95	515.0	533.58	0.59	519.0	537.0	1.37	519.0	527.55	1.37	517.0	532.71	0.98
43	516	521.0	530.06	0.97	524.0	532.32	1.55	516.0	530.1	0.0	520.0	532.84	0.78	516.0	527.03	0.0	520.0	528.23	0.78
44	494	495.0	508.74	0.2	502.0	512.55	1.62	497.0	510.19	0.61	498.0	511.65	0.81	495.0	504.71	0.2	500.0	511.03	1.21
45	512	518.0	528.48	1.17	518.0	530.26	1.17	513.0	527.87	0.2	521.0	531.0	1.76	518.0	524.77	1.17	517.0	527.03	0.98
46	560	563.0	567.94	0.54	566.0	572.13	1.07	564.0	571.06	0.71	566.0	573.71	1.07	563.0	567.68	0.54	563.0	568.39	0.54
47	430	434.0	438.29	0.93	433.0	439.39	0.7	430.0	437.9	0.0	434.0	440.35	0.93	430.0	436.35	0.0	434.0	437.32	0.93
48	492	496.0	499.39	0.81	496.0	501.52	0.81	494.0	500.84	0.41	497.0	506.26	1.02	494.0	498.74	0.41	493.0	500.87	0.2
49	641	648.0	671.58	1.09	666.0	679.52	3.9	651.0	674.61	1.56	655.0	679.65	2.18	660.0	671.1	2.96	656.0	672.32	2.34
410	514	515.0	521.19	0.19	518.0	523.9	0.78	515.0	523.77	0.19	518.0	524.42	0.78	515.0	520.26	0.19	516.0	521.35	0.39
51	253	257.0	261.84	1.58	258.0	264.32	1.98	254.0	263.42	0.4	258.0	265.0	1.98	254.0	261.58	0.4	256.0	262.74	1.19
52	302	311.0	322.87	2.98	312.0	324.65	3.31	313.0	323.55	3.64	314.0	324.97	3.97	314.0	322.45	3.97	313.0	322.97	3.64
53	226	228.0	230.55	0.88	229.0	231.29	1.33	229.0	231.03	1.33	229.0	231.19	1.33	228.0	230.35	0.88	229.0	230.45	1.33
54	242	245.0	249.03	1.24	245.0	250.16	1.24	243.0	248.68	0.41	244.0	249.55	0.83	244.0	247.9	0.83	245.0	249.26	1.24
55	211	212.0	215.52	0.47	211.0	216.0	0.0	212.0	215.94	0.47	212.0	216.06	0.47	211.0	214.29	0.0	212.0	215.1	0.47
56	213	213.0	220.03	0.0	217.0	222.71	1.88	216.0	221.9	1.41	213.0	222.39	0.0	214.0	219.35	0.47	214.0	220.55	0.47
57	293	297.0	302.74	1.37	296.0	305.03	1.02	294.0	305.1	0.34	298.0	306.29	1.71	296.0	302.1	1.02	297.0	303.29	1.37
58	288	288.0	293.32	0.0	289.0	294.97	0.35	289.0	296.71	0.35	290.0	297.84	0.69	289.0	291.97	0.35	289.0	293.1	0.35
59	279	280.0	284.19	0.36	281.0	285.65	0.72	281.0	286.45	0.72	280.0	286.13	0.36	280.0	283.97	0.36	280.0	283.29	0.36
510	265	265.0	273.1	0.0	268.0	273.68	1.13	269.0	274.32	1.51	266.0	273.48	0.38	267.0	271.16	0.75	266.0	272.13	0.38
61	138	140.0	142.9	1.45	138.0	144.77	0.0	140.0	143.9	1.45	141.0	144.71	2.17	140.0	143.13	1.45	141.0	143.32	2.17
62	146	146.0	151.03	0.0	147.0	152.55	0.68	146.0	153.0	0.0	148.0	154.45	1.37	147.0	149.87	0.68	146.0	150.77	0.0
63	145	145.0	148.45	0.0	148.0	149.52	2.07	146.0	150.23	0.69	145.0	150.06	0.0	145.0	147.94	0.0	145.0	148.55	0.0
64	131	131.0	133.1	0.0	132.0	133.65	0.76	131.0	134.35	0.0	131.0	135.03	0.0	131.0	132.65	0.0	131.0	133.16	0.0
65	161	161.0	168.65	0.0	162.0	170.74	0.62	164.0	171.68	1.86	164.0	174.74	1.86	162.0	167.35	0.62	161.0	168.58	0.0
a1	253	258.0	262.16	1.98	261.0	264.39	3.16	258.0	263.65	1.98	258.0	265.13	1.98	258.0	261.26	1.98	258.0	262.23	1.98
a2	252	258.0	263.9	2.38	258.0	265.74	2.38	260.0	267.48	3.17	257.0	266.94	1.98	255.0	262.0	1.19	255.0	264.23	1.19
a3	232	237.0	242.71	2.16	238.0	243.61	2.59	238.0	244.58	2.59	238.0	244.61	2.59	237.0	241.55	2.16	238.0	243.03	2.59
a4	234	237.0	243.23	1.28	239.0	244.9	2.14	239.0	246.94	2.14	236.0	246.32	0.85	236.0	241.39	0.85	239.0	243.52	2.14
a5	236	237.0	242.71	0.42	240.0	244.65	1.69	238.0	244.45	0.85	241.0	245.71	2.12	240.0	242.39	1.69	238.0	243.13	0.85
b1	69	69.0	70.29	0.0	69.0	70.97	0.0	69.0	71.32	0.0	69.0	72.16	0.0	69.0	69.74	0.0	69.0	70.58	0.0
b2	76	76.0	77.32	0.0	76.0	78.9	0.0	76.0	79.42	0.0	76.0	81.06	0.0	76.0	76.48	0.0	76.0	77.52	0.0
b3	80	80.0	81.39	0.0	80.0	81.9	0.0	81.0	83.16	1.25	80.0	82.65	0.0	80.0	81.26	0.0	81.0	81.55	1.25
b4	79	79.0	81.87	0.0	80.0	82.97	1.27	79.0	83.0	0.0	79.0	84.58	0.0	79.0	80.84	0.0	79.0	81.61	0.0
b5	72	72.0	72.9	0.0	72.0	73.61	0.0	72.0	73.77	0.0	72.0	74.48	0.0	72.0	72.65	0.0	72.0	73.19	0.0
c1	227	231.0	238.23	1.76	232.0	240.1	2.2	233.0	241.1	2.64	235.0	244.9	3.52	231.0	236.13	1.76	233.0	238.39	2.64
c2	219	225.0	230.97	2.74	227.0	233.87	3.65	223.0	233.06	1.83	226.0	236.39	3.2	224.0	229.35	2.28	224.0	230.97	2.28
c3	243	245.0	253.68	0.82	249.0	257.03	2.47	250.0	257.97	2.88	249.0	260.39	2.47	245.0	252.45	0.82	247.0	254.52	1.65
c4	219	222.0	230.26	1.37	225.0	233.23	2.74	227.0	236.13	3.65	227.0	234.42	3.65	222.0	227.97	1.37	225.0	230.52	2.74
c5	215	220.0	223.9	2.33	220.0	224.06	2.33	219.0	226.35	1.86	219.0	229.35	1.86	218.0	221.65	1.4	219.0	223.58	1.86
d1	60	60.0	62.32	0.0	60.0	63.32	0.0	60.0	63.65	0.0	62.0	64.32	3.33	61.0	62.39	1.67	60.0	62.42	0.0
d2	66	66.0	67.48	0.0	67.0	68.48	1.52	67.0	68.84	1.52	67.0	69.94	1.52	67.0	67.48	1.52	67.0	67.97	1.52
d3	72	73.0	74.77	1.39	74.0	76.48	2.78	72.0	76.97	0.0	75.0	77.81	4.17	73.0	74.77	1.39	73.0	76.06	1.39
d4	62	62.0	62.84	0.0	62.0	64.1	0.0	62.0	64.23	0.0	63.0	66.19	1.61	62.0	62.45	0.0	62.0	63.06	0.0
d5	61	61.0	62.61	0.0	61.0	64.0	0.0	61.0	63.97	0.0	61.0	66.84	0.0	61.0	62.0	0.0	61.0	62.68	0.0
		256.04	261.6	0.79	257.76	263.53	1.38	256.38	263.46	1.02	257.38	264.86	1.4	256.18	260.51	0.87	256.62	261.81	1.02

**Table A3:** Comparison of the GWO metaheuristics.

		BCL			MIR			BQSA_40a_pl0			BQSA_40a_pl4			BQSA_80a_pl0			BQSA_80a_pl4		
Inst.	Opt.	Best	Avg	RPD	Best	Avg	RPD	Best	Avg	RPD	Best	Avg	RPD	Best	Avg	RPD	Best	Avg	RPD
41	429	431.0	437.32	0.47	431.0	436.81	0.47	430.0	433.68	0.23	430.0	433.94	0.23	431.0	433.58	0.47	430.0	433.77	0.23
42	512	526.0	540.58	2.73	540.0	551.26	5.47	516.0	531.68	0.78	517.0	534.87	0.98	520.0	530.52	1.56	520.0	530.94	1.56
43	516	519.0	535.84	0.58	535.0	550.26	3.68	519.0	529.16	0.58	517.0	532.84	0.19	520.0	528.52	0.78	516.0	529.68	0.0
44	494	503.0	520.35	1.82	513.0	524.45	3.85	496.0	507.61	0.4	501.0	512.48	1.42	497.0	507.77	0.61	499.0	508.55	1.01
45	512	523.0	533.84	2.15	532.0	551.39	3.91	518.0	527.55	1.17	518.0	531.19	1.17	517.0	525.16	0.98	519.0	529.52	1.37
46	560	570.0	583.58	1.79	576.0	586.03	2.86	562.0	569.87	0.36	565.0	570.65	0.89	563.0	568.94	0.54	565.0	569.13	0.89
47	430	434.0	441.77	0.93	438.0	447.58	1.86	433.0	436.77	0.7	435.0	438.74	1.16	433.0	437.26	0.7	435.0	437.68	1.16
48	492	499.0	509.84	1.42	506.0	515.13	2.85	493.0	500.61	0.2	496.0	501.45	0.81	494.0	499.32	0.41	496.0	501.19	0.81
49	641	656.0	680.61	2.34	679.0	693.06	5.93	657.0	673.03	2.5	658.0	676.16	2.65	662.0	673.58	3.28	660.0	671.77	2.96
410	514	520.0	532.68	1.17	526.0	532.74	2.33	516.0	522.06	0.39	517.0	523.39	0.58	515.0	520.61	0.19	515.0	521.61	0.19
51	253	259.0	268.23	2.37	262.0	268.26	3.56	256.0	263.03	1.19	254.0	263.0	0.4	254.0	260.94	0.4	255.0	262.9	0.79
52	302	320.0	330.19	5.96	326.0	335.39	7.95	316.0	323.13	4.64	310.0	325.35	2.65	313.0	323.48	3.64	315.0	324.42	4.3
53	226	229.0	235.55	1.33	232.0	236.32	2.65	229.0	230.87	1.33	229.0	231.42	1.33	228.0	230.39	0.88	229.0	230.65	1.33
54	242	244.0	251.55	0.83	250.0	254.68	3.31	244.0	249.0	0.83	246.0	249.87	1.65	244.0	248.97	0.83	244.0	249.58	0.83
55	211	212.0	216.74	0.47	216.0	219.32	2.37	212.0	214.81	0.47	212.0	215.16	0.47	211.0	214.74	0.0	212.0	215.13	0.47
56	213	216.0	226.77	1.41	222.0	230.03	4.23	214.0	218.97	0.47	214.0	221.35	0.47	213.0	218.9	0.0	215.0	220.1	0.94
57	293	299.0	310.84	2.05	306.0	314.29	4.44	295.0	302.81	0.68	297.0	304.9	1.37	297.0	302.81	1.37	297.0	304.06	1.37
58	288	290.0	298.77	0.69	297.0	302.97	3.12	291.0	294.06	1.04	289.0	294.19	0.35	289.0	292.71	0.35	288.0	292.87	0.0
59	279	281.0	288.39	0.72	285.0	293.71	2.15	281.0	284.06	0.72	280.0	284.13	0.36	281.0	283.65	0.72	281.0	283.97	0.72
510	265	269.0	276.81	1.51	277.0	282.16	4.53	266.0	271.77	0.38	267.0	273.23	0.75	266.0	271.19	0.38	267.0	272.74	0.75
61	138	140.0	146.94	1.45	144.0	149.1	4.35	141.0	142.97	2.17	141.0	143.71	2.17	141.0	142.97	2.17	140.0	143.13	1.45
62	146	147.0	154.32	0.68	155.0	161.23	6.16	146.0	150.55	0.0	148.0	152.19	1.37	146.0	150.71	0.0	146.0	151.68	0.0
63	145	145.0	152.03	0.0	148.0	154.32	2.07	145.0	148.35	0.0	145.0	148.94	0.0	145.0	148.45	0.0	147.0	148.52	1.38
64	131	131.0	136.23	0.0	134.0	136.26	2.29	131.0	132.55	0.0	131.0	133.23	0.0	131.0	132.42	0.0	131.0	132.97	0.0
65	161	167.0	175.13	3.73	175.0	184.61	8.7	161.0	167.65	0.0	164.0	170.45	1.86	162.0	168.9	0.62	162.0	168.87	0.62
a1	253	262.0	267.0	3.56	265.0	279.84	4.74	258.0	263.03	1.98	259.0	264.65	2.37	260.0	262.87	2.77	259.0	262.61	2.37
a2	252	262.0	271.52	3.97	275.0	283.13	9.13	259.0	264.77	2.78	260.0	265.65	3.17	257.0	263.74	1.98	259.0	264.1	2.78
a3	232	238.0	248.16	2.59	246.0	257.84	6.03	236.0	242.48	1.72	236.0	243.19	1.72	235.0	241.68	1.29	239.0	242.71	3.02
a4	234	238.0	250.94	1.71	250.0	263.19	6.84	239.0	243.23	2.14	237.0	243.74	1.28	239.0	242.32	2.14	239.0	243.42	2.14
a5	236	244.0	250.0	3.39	253.0	261.84	7.2	239.0	243.52	1.27	238.0	244.13	0.85	240.0	243.0	1.69	240.0	243.58	1.69
b1	69	69.0	72.26	0.0	75.0	89.94	8.7	69.0	70.42	0.0	69.0	70.23	0.0	69.0	69.87	0.0	69.0	70.29	0.0
b2	76	76.0	80.71	0.0	86.0	97.87	13.16	76.0	77.39	0.0	76.0	77.71	0.0	76.0	77.39	0.0	76.0	76.97	0.0
b3	80	81.0	83.97	1.25	89.0	107.29	11.25	80.0	81.39	0.0	81.0	81.77	1.25	80.0	81.32	0.0	81.0	81.52	1.25
b4	79	81.0	84.16	2.53	93.0	105.9	17.72	80.0	81.87	1.27	80.0	82.26	1.27	79.0	81.39	0.0	79.0	81.81	0.0
b5	72	73.0	74.77	1.39	81.0	93.32	12.5	72.0	72.97	0.0	72.0	73.26	0.0	72.0	72.68	0.0	72.0	73.03	0.0
c1	227	239.0	249.26	5.29	258.0	270.35	13.66	235.0	239.13	3.52	235.0	240.84	3.52	234.0	238.52	3.08	234.0	239.35	3.08
c2	219	228.0	239.52	4.11	252.0	267.19	15.07	224.0	231.55	2.28	224.0	232.06	2.28	225.0	229.81	2.74	224.0	231.23	2.28
c3	243	252.0	260.94	3.7	279.0	294.81	14.81	248.0	254.1	2.06	248.0	253.48	2.06	246.0	252.52	1.23	248.0	253.03	2.06
c4	219	228.0	237.32	4.11	250.0	263.61	14.16	222.0	229.42	1.37	226.0	229.84	3.2	225.0	229.16	2.74	225.0	229.87	2.74
c5	215	222.0	230.68	3.26	243.0	259.06	13.02	220.0	223.58	2.33	219.0	224.29	1.86	220.0	223.13	2.33	220.0	223.77	2.33
d1	60	61.0	64.65	1.67	83.0	104.32	38.33	61.0	62.39	1.67	61.0	62.68	1.67	60.0	61.94	0.0	61.0	62.65	1.67
d2	66	66.0	68.9	0.0	87.0	124.68	31.82	66.0	67.71	0.0	67.0	68.0	1.52	66.0	67.58	0.0	67.0	67.74	1.52
d3	72	75.0	77.81	4.17	106.0	130.84	47.22	72.0	75.87	0.0	74.0	76.26	2.78	73.0	75.0	1.39	73.0	75.29	1.39
d4	62	62.0	65.35	0.0	90.0	111.87	45.16	62.0	62.81	0.0	62.0	62.97	0.0	62.0	62.48	0.0	62.0	62.55	0.0
d5	61	61.0	64.68	0.0	85.0	109.42	39.34	61.0	62.48	0.0	61.0	63.58	0.0	61.0	62.58	0.0	61.0	62.58	0.0
		258.84	267.28	1.9	270.02	281.95	10.33	256.6	261.7	1.01	257.02	262.83	1.25	256.71	261.23	0.98	257.16	261.86	1.23
		SA_40a_pl0			SA_40a_pl4			SA_80a_pl0			SA_80a_pl4			MAB_40a_pl0			MAB_40a_pl4		
Inst.	Opt.	Best	Avg	RPD	Best	Avg	RPD	Best	Avg	RPD	Best	Avg	RPD	Best	Avg	RPD	Best	Avg	RPD
41	429	430.0	434.39	0.23	430.0	433.84	0.23	430.0	433.81	0.23	430.0	433.68	0.23	430.0	433.58	0.23	430.0	435.68	0.23
42	512	518.0	532.97	1.17	522.0	535.77	1.95	518.0	532.48	1.17	521.0	531.42	1.76	518.0	529.74	1.17	526.0	537.81	2.73
43	516	521.0	530.29	0.97	525.0	536.16	1.74	522.0	529.71	1.16	521.0	529.26	0.97	517.0	528.26	0.19	524.0	536.61	1.55
44	494	500.0	509.35	1.21	497.0	512.65	0.61	498.0	507.77	0.81	497.0	506.06	0.61	495.0	505.84	0.2	506.0	516.0	2.43
45	512	514.0	529.45	0.39	524.0	534.65	2.34	520.0	528.81	1.56	520.0	526.23	1.56	518.0	525.48	1.17	527.0	535.0	2.93
46	560	564.0	569.77	0.71	565.0	573.1	0.89	562.0	569.03	0.36	562.0	568.29	0.36	562.0	569.35	0.36	565.0	572.32	0.89
47	430	434.0	438.87	0.93	434.0	440.1	0.93	434.0	437.55	0.93	434.0	436.97	0.93	432.0	436.65	0.47	435.0	440.32	1.16
48	492	494.0	500.48	0.41	496.0	504.23	0.81	494.0	500.32	0.41	493.0	499.06	0.2	493.0	499.74	0.2	497.0	503.16	1.02
49	641	659.0	675.55	2.81	662.0	678.55	3.28	660.0	674.13	2.96	657.0	674.03	2.5	655.0	671.1	2.18	662.0	677.68	3.28
410	514	516.0	521.84	0.39	517.0	525.61	0.58	516.0	521.65	0.39	516.0	521.35	0.39	516.0	521.52	0.39	519.0	524.13	0.97
51	253	257.0	263.61	1.58	256.0	263.06	1.19	256.0	261.94	1.19	257.0	261.16	1.58	257.0	263.19	1.58	259.0	263.87	2.37
52	302	319.0	325.29	5.63	318.0	326.61	5.3	315.0	324.35	4.3	316.0	323.39	4.64	315.0	322.94	4.3	316.0	325.9	4.64
53	226	229.0	230.65	1.33	230.0	232.19	1.77	228.0	230.03	0.88	229.0	230.35	1.33	229.0	230.87	1.33	229.0	231.42	1.33
54	242	245.0	249.87	1.24	246.0	250.52	1.65	244.0	249.13	0.83	245.0	248.74	1.24	243.0	248.84	0.41	245.0	250.03	1.24
55	211	212.0	214.68	0.47	212.0	216.23	0.47	212.0	215.06	0.47	211.0	214.39	0.0	212.0	214.81	0.47	213.0	215.87	0.95
56	213	213.0	220.13	0.0	218.0	223.71	2.35	213.0	219.35	0.0	213.0	218.84	0.0	213.0	220.74	0.0	217.0	222.16	1.88
57	293	298.0	304.0	1.71	299.0	306.58	2.05	296.0	303.29	1.02	296.0	302.58	1.02	295.0	303.23	0.68	297.0	306.13	1.37
58	288	289.0	293.32	0.35	290.0	295.03	0.69	289.0	292.32	0.35	288.0	292.9	0.0	288.0	292.19	0.0	289.0	294.97	0.35
59	279	281.0	284.71	0.72	281.0	285.58	0.72	280.0	283.29	0.36	281.0	283.81	0.72	280.0	283.39	0.36	282.0	285.71	1.08
510	265	265.0	271.81	0.0	267.0	274.35	0.75	265.0	272.13	0.0	265.0	271.23	0.0	265.0	270.74	0.0	268.0	275.03	1.13
61	138	139.0	143.0	0.72	141.0	144.48	2.17	141.0	142.84	2.17	140.0	143.13	1.45	139.0	143.42	0.72	142.0	144.84	2.9
62	146	147.0	152.06	0.68	146.0	152.74	0.0	148.0	151.71	1.37	146.0	150.97	0.0	146.0	150.55	0.0	149.0	153.03	2.05
63	145	145.0	148.74	0.0	145.0	148.71	0.0	146.0	148.42	0.69	145.0	148.48	0.0	146.0	148.29	0.69	147.0	148.74	1.38
64	131	131.0	133.29	0.0	131.0	133.16	0.0	131.0	132.45	0.0	131.0	132.9	0.0	131.0	132.68	0.0	132.0	133.87	0.76
65	161	161.0	168.61	0.0	162.0	171.13	0.62	162.0	168.32	0.62	161.0	167.71	0.0	161.0	166.61	0.0	164.0	172.97	1.86
a1	253	259.0	263.68	2.37	259.0	263.81	2.37	260.0	263.0	2.77	260.0	262.42	2.77	259.0	263.19	2.37	260.0	265.32	2.77
a2	252	258.0	265.55	2.38	261.0	267.19	3.57	256.0	262.23	1.59	258.0	263.1	2.38	257.0	265.35	1.98	261.0	267.06	3.57
a3	232	237.0	242.9	2.16	239.0	244.97	3.02	238.0	241.55	2.59	237.0	242.0	2.16	239.0	242.35	3.02	238.0	244.52	2.59
a4	234	238.0	243.42	1.71	241.0	245.71	2.99	237.0	242.48	1.28	237.0	242.42	1.28	237.0	244.94	1.28	239.0	247.42	2.14
a5	236	241.0	244.26	2.12	241.0	246.35	2.12	239.0	243.13	1.27	240.0	242.65	1.69	240.0	243.74	1.69	241.0	245.71	2.12
b1	69	69.0	70.26	0.0	69.0	70.77	0.0	69.0	69.68	0.0	69.0	70.13	0.0	69.0	70.16	0.0	69.0	71.13	0.0
b2	76	76.0	77.19	0.0	77.0	78.94	1.32	76.0	76.65	0.0	76.0	77.06	0.0	76.0	76.97	0.0	77.0	78.94	1.32
b3	80	80.0	81.45	0.0	81.0	82.16	1.25	80.0	81.35	0.0	80.0	81.06	0.0	80.0	81.16	0.0	81.0	82.35	1.25
b4	79	79.0	81.45	0.0	80.0	82.87	1.27	79.0	81.45	0.0	79.0	81.32	0.0	79.0	81.52	0.0	81.0	83.32	2.53
b5	72	72.0	72.9	0.0	72.0	73.68	0.0	72.0	72.94	0.0	72.0	72.77	0.0	72.0	72.55	0.0	72.0	73.74	0.0
c1	227	232.0	239.45	2.2	237.0	243.29	4.41	235.0	237.97	3.52	234.0	238.29	3.08	232.0	239.29	2.2	237.0	243.61	4.41
c2	219	226.0	231.84	3.2	226.0	234.26	3.2	223.0	230.65	1.83	227.0	230.45	3.65	224.0	230.71	2.28	225.0	234.65	2.74
c3	243	247.0	254.29	1.65	247.0	254.81	1.65	246.0	250.68	1.23	247.0	251.13	1.65	246.0	253.58	1.23	248.0	257.29	2.06
c4	219	224.0	229.77	2.28	225.0	231.23	2.74	225.0	228.71	2.74	222.0	228.61	1.37	225.0	230.19	2.74	223.0	232.0	1.83
c5	215	219.0	224.0	1.86	221.0	226.87	2.79	220.0	222.87	2.33	219.0	222.65	1.86	219.0	224.29	1.86	224.0	228.1	4.19
d1	60	60.0	62.23	0.0	60.0	62.87	0.0	60.0	61.9	0.0	61.0	61.87	1.67	61.0	62.16	1.67	61.0	63.74	1.67
d2	66	67.0	67.9	1.52	67.0	68.35	1.52	67.0	67.55	1.52	67.0	67.61	1.52	66.0	67.52	0.0	67.0	68.52	1.52
d3	72	73.0	75.84	1.39	74.0	76.32	2.78	73.0	75.26	1.39	73.0	75.13	1.39	73.0	75.1	1.39	74.0	76.77	2.78
d4	62	62.0	63.06	0.0	62.0	63.39	0.0	62.0	62.35	0.0	62.0	62.74	0.0	62.0	62.9	0.0	62.0	63.65	0.0
d5	61	61.0	62.68	0.0	61.0	64.03	0.0	61.0	62.1	0.0	61.0	62.65	0.0	61.0	62.35	0.0	62.0	64.19	1.64
		256.91	262.24	1.08	258.09	264.01	1.56	256.84	261.43	1.07	256.8	261.18	1.07	256.29	261.42	0.91	258.71	264.34	1.86
		MAB_80a_pl0			MAB_80a_pl4			QL_40a_pl0			QL_40a_pl4			QL_80a_pl0			QL_80a_pl4		
Inst.	Opt.	Best	Avg	RPD	Best	Avg	RPD	Best	Avg	RPD	Best	Avg	RPD	Best	Avg	RPD	Best	Avg	RPD
41	429	430.0	432.81	0.23	430.0	433.06	0.23	430.0	433.06	0.23	430.0	434.45	0.23	430.0	433.19	0.23	430.0	432.94	0.23
42	512	518.0	528.94	1.17	521.0	528.94	1.76	522.0	530.26	1.95	517.0	532.16	0.98	518.0	528.13	1.17	519.0	530.9	1.37
43	516	522.0	528.06	1.16	520.0	526.55	0.78	519.0	527.29	0.58	522.0	531.77	1.16	521.0	527.16	0.97	519.0	528.97	0.58
44	494	496.0	507.74	0.4	496.0	505.74	0.4	500.0	506.84	1.21	499.0	508.32	1.01	499.0	506.97	1.01	496.0	507.45	0.4
45	512	518.0	524.84	1.17	520.0	526.0	1.56	521.0	527.0	1.76	521.0	528.16	1.76	518.0	524.39	1.17	522.0	528.16	1.95
46	560	561.0	567.77	0.18	563.0	568.48	0.54	563.0	568.19	0.54	564.0	570.32	0.71	563.0	566.94	0.54	563.0	568.55	0.54
47	430	432.0	436.81	0.47	433.0	436.03	0.7	433.0	437.55	0.7	433.0	437.84	0.7	432.0	436.45	0.47	433.0	437.13	0.7
48	492	494.0	500.06	0.41	494.0	498.52	0.41	493.0	498.61	0.2	493.0	500.48	0.2	494.0	498.45	0.41	494.0	500.58	0.41
49	641	657.0	668.9	2.5	660.0	671.1	2.96	654.0	672.45	2.03	663.0	675.32	3.43	656.0	667.55	2.34	659.0	674.45	2.81
410	514	515.0	520.71	0.19	516.0	520.23	0.39	515.0	520.13	0.19	516.0	523.42	0.39	517.0	520.16	0.58	516.0	521.68	0.39
51	253	254.0	262.39	0.4	255.0	260.81	0.79	255.0	260.68	0.79	257.0	263.06	1.58	256.0	260.13	1.19	255.0	261.74	0.79
52	302	313.0	321.81	3.64	311.0	322.48	2.98	311.0	322.9	2.98	316.0	325.03	4.64	314.0	322.48	3.97	316.0	322.74	4.64
53	226	228.0	230.29	0.88	229.0	229.97	1.33	229.0	230.52	1.33	229.0	231.16	1.33	229.0	230.35	1.33	229.0	230.74	1.33
54	242	244.0	248.03	0.83	245.0	248.32	1.24	244.0	247.71	0.83	243.0	250.32	0.41	246.0	248.52	1.65	247.0	249.68	2.07
55	211	212.0	214.35	0.47	212.0	214.1	0.47	212.0	214.81	0.47	212.0	215.32	0.47	213.0	214.61	0.95	212.0	214.71	0.47
56	213	213.0	219.68	0.0	216.0	219.42	1.41	213.0	218.23	0.0	214.0	220.97	0.47	214.0	218.58	0.47	215.0	220.52	0.94
57	293	294.0	302.19	0.34	295.0	302.39	0.68	297.0	302.29	1.37	297.0	304.03	1.37	297.0	301.94	1.37	295.0	302.23	0.68
58	288	288.0	290.97	0.0	289.0	291.35	0.35	290.0	292.58	0.69	288.0	294.32	0.0	289.0	291.81	0.35	288.0	292.03	0.0
59	279	280.0	282.71	0.36	281.0	283.42	0.72	280.0	283.42	0.36	282.0	285.13	1.08	280.0	282.65	0.36	281.0	283.29	0.72
510	265	266.0	271.74	0.38	265.0	270.19	0.0	265.0	271.03	0.0	265.0	273.71	0.0	267.0	271.77	0.75	266.0	271.81	0.38
61	138	141.0	142.55	2.17	140.0	142.35	1.45	140.0	142.61	1.45	140.0	143.65	1.45	140.0	142.16	1.45	140.0	142.84	1.45
62	146	146.0	149.68	0.0	146.0	150.16	0.0	146.0	150.97	0.0	146.0	151.65	0.0	148.0	151.0	1.37	147.0	151.1	0.68
63	145	145.0	148.03	0.0	145.0	147.9	0.0	145.0	148.32	0.0	146.0	148.52	0.69	145.0	147.84	0.0	146.0	148.42	0.69
64	131	131.0	132.42	0.0	131.0	132.84	0.0	131.0	132.81	0.0	131.0	133.35	0.0	131.0	131.97	0.0	131.0	132.81	0.0
65	161	161.0	167.16	0.0	161.0	167.55	0.0	161.0	165.1	0.0	164.0	170.16	1.86	163.0	166.9	1.24	162.0	166.61	0.62
a1	253	258.0	261.87	1.98	258.0	262.35	1.98	259.0	262.45	2.37	259.0	264.52	2.37	259.0	262.23	2.37	259.0	262.87	2.37
a2	252	257.0	262.0	1.98	257.0	263.42	1.98	256.0	263.55	1.59	259.0	265.58	2.78	259.0	264.29	2.78	258.0	263.94	2.38
a3	232	238.0	241.32	2.59	238.0	242.06	2.59	235.0	242.94	1.29	241.0	244.68	3.88	236.0	241.94	1.72	237.0	242.97	2.16
a4	234	237.0	243.19	1.28	236.0	242.61	0.85	237.0	242.39	1.28	237.0	244.35	1.28	238.0	242.16	1.71	237.0	242.94	1.28
a5	236	239.0	242.97	1.27	238.0	243.71	0.85	238.0	243.23	0.85	238.0	244.1	0.85	240.0	243.29	1.69	238.0	243.52	0.85
b1	69	69.0	69.74	0.0	69.0	70.0	0.0	69.0	69.97	0.0	69.0	70.58	0.0	69.0	69.68	0.0	69.0	69.81	0.0
b2	76	76.0	76.65	0.0	76.0	76.68	0.0	76.0	77.13	0.0	76.0	77.61	0.0	76.0	76.58	0.0	76.0	76.71	0.0
b3	80	80.0	81.19	0.0	80.0	81.32	0.0	81.0	81.52	1.25	80.0	81.65	0.0	81.0	81.13	1.25	80.0	81.32	0.0
b4	79	79.0	80.65	0.0	80.0	81.26	1.27	80.0	81.58	1.27	79.0	82.03	0.0	79.0	81.29	0.0	79.0	80.97	0.0
b5	72	72.0	72.68	0.0	72.0	72.84	0.0	72.0	72.97	0.0	72.0	73.39	0.0	72.0	72.58	0.0	72.0	72.77	0.0
c1	227	233.0	237.19	2.64	235.0	238.19	3.52	234.0	238.48	3.08	236.0	241.06	3.96	235.0	238.06	3.52	235.0	239.0	3.52
c2	219	223.0	228.77	1.83	227.0	230.74	3.65	225.0	231.19	2.74	222.0	232.58	1.37	224.0	230.42	2.28	224.0	230.39	2.28
c3	243	246.0	251.19	1.23	247.0	252.26	1.65	247.0	252.06	1.65	248.0	254.06	2.06	246.0	250.19	1.23	247.0	252.13	1.65
c4	219	223.0	228.23	1.83	224.0	229.55	2.28	224.0	229.13	2.28	225.0	230.52	2.74	224.0	227.32	2.28	224.0	229.13	2.28
c5	215	218.0	222.87	1.4	217.0	222.58	0.93	219.0	223.29	1.86	219.0	225.77	1.86	220.0	222.48	2.33	221.0	223.97	2.79
d1	60	60.0	61.32	0.0	60.0	62.23	0.0	61.0	61.97	1.67	60.0	62.71	0.0	60.0	62.16	0.0	60.0	62.48	0.0
d2	66	66.0	67.58	0.0	66.0	67.71	0.0	66.0	67.48	0.0	66.0	68.13	0.0	66.0	67.71	0.0	67.0	67.87	1.52
d3	72	72.0	74.26	0.0	73.0	75.06	1.39	73.0	75.45	1.39	74.0	75.87	2.78	73.0	74.32	1.39	73.0	75.29	1.39
d4	62	62.0	62.26	0.0	62.0	62.68	0.0	62.0	62.81	0.0	62.0	62.94	0.0	62.0	62.35	0.0	62.0	62.71	0.0
d5	61	61.0	62.1	0.0	61.0	62.52	0.0	61.0	62.35	0.0	62.0	63.32	1.64	61.0	62.0	0.0	61.0	62.45	0.0
		256.18	260.64	0.79	256.67	260.84	0.98	256.53	261.05	0.98	257.16	262.62	1.19	256.89	260.54	1.11	256.89	261.45	1.1

**Table A4:** Comparison of the SCA metaheuristics.

		BCL			MIR			BQSA_40a_pl0			BQSA_40a_pl4			BQSA_80a_pl0			BQSA_80a_pl4		
Inst.	Opt.	Best	Avg	RPD	Best	Avg	RPD	Best	Avg	RPD	Best	Avg	RPD	Best	Avg	RPD	Best	Avg	RPD
41	429	505.0	606.16	17.72	679.0	716.9	58.28	430.0	432.35	0.23	430.0	432.94	0.23	430.0	432.52	0.23	430.0	432.9	0.23
42	512	600.0	785.13	17.19	1060.0	1211.42	107.03	516.0	521.94	0.78	513.0	522.39	0.2	514.0	521.84	0.39	515.0	520.9	0.59
43	516	589.0	794.94	14.15	1185.0	1295.61	129.65	517.0	522.19	0.19	516.0	522.58	0.0	516.0	522.35	0.0	516.0	520.68	0.0
44	494	564.0	728.87	14.17	962.0	1051.61	94.74	495.0	499.19	0.2	494.0	498.74	0.0	494.0	498.97	0.0	494.0	497.84	0.0
45	512	615.0	815.74	20.12	1126.0	1222.71	119.92	517.0	520.32	0.98	514.0	520.42	0.39	514.0	520.32	0.39	515.0	519.97	0.59
46	560	638.0	867.52	13.93	1331.0	1485.23	137.68	561.0	564.26	0.18	560.0	565.13	0.0	560.0	564.97	0.0	561.0	564.84	0.18
47	430	491.0	645.19	14.19	877.0	946.1	103.95	430.0	434.26	0.0	432.0	434.39	0.47	431.0	434.42	0.23	430.0	434.1	0.0
48	492	577.0	798.84	17.28	1119.0	1257.0	127.44	493.0	495.13	0.2	493.0	496.0	0.2	493.0	495.61	0.2	493.0	495.45	0.2
49	641	766.0	995.9	19.5	1535.0	1695.74	139.47	653.0	661.61	1.87	650.0	662.61	1.4	650.0	661.48	1.4	646.0	658.45	0.78
410	514	576.0	778.9	12.06	1104.0	1210.84	114.79	514.0	518.19	0.0	514.0	516.97	0.0	514.0	517.45	0.0	514.0	517.32	0.0
51	253	302.0	383.0	19.37	568.0	625.13	124.51	254.0	258.0	0.4	253.0	257.58	0.0	254.0	258.58	0.4	253.0	257.52	0.0
52	302	356.0	475.77	17.88	748.0	919.45	147.68	308.0	316.71	1.99	313.0	318.03	3.64	309.0	318.58	2.32	307.0	317.52	1.66
53	226	265.0	346.87	17.26	498.0	561.35	120.35	228.0	229.65	0.88	228.0	229.55	0.88	228.0	229.55	0.88	228.0	229.39	0.88
54	242	281.0	356.9	16.12	534.0	590.9	120.66	243.0	246.77	0.41	242.0	246.13	0.0	243.0	246.55	0.41	242.0	245.81	0.0
55	211	224.0	318.19	6.16	411.0	438.87	94.79	211.0	212.52	0.0	211.0	212.61	0.0	211.0	212.16	0.0	211.0	212.52	0.0
56	213	242.0	347.13	13.62	472.0	539.23	121.6	213.0	215.94	0.0	213.0	215.03	0.0	213.0	215.35	0.0	213.0	215.06	0.0
57	293	345.0	464.58	17.75	643.0	717.23	119.45	294.0	299.77	0.34	294.0	299.58	0.34	294.0	300.55	0.34	295.0	299.9	0.68
58	288	350.0	447.45	21.53	650.0	739.77	125.69	288.0	290.16	0.0	288.0	289.52	0.0	288.0	290.0	0.0	288.0	289.32	0.0
59	279	313.0	424.77	12.19	682.0	751.87	144.44	280.0	281.32	0.36	280.0	281.23	0.36	280.0	281.23	0.36	280.0	281.23	0.36
510	265	305.0	419.77	15.09	621.0	673.71	134.34	265.0	268.87	0.0	265.0	268.35	0.0	265.0	268.06	0.0	265.0	268.26	0.0
61	138	171.0	267.29	23.91	669.0	834.23	384.78	139.0	141.68	0.72	138.0	141.71	0.0	138.0	141.52	0.0	138.0	141.48	0.0
62	146	188.0	327.42	28.77	1086.0	1199.0	643.84	146.0	148.52	0.0	146.0	149.03	0.0	146.0	148.13	0.0	146.0	148.19	0.0
63	145	158.0	308.23	8.97	1057.0	1147.9	628.97	145.0	146.87	0.0	145.0	146.77	0.0	145.0	146.9	0.0	145.0	146.32	0.0
64	131	159.0	299.71	21.37	623.0	729.03	375.57	131.0	131.42	0.0	131.0	131.84	0.0	131.0	131.58	0.0	131.0	131.39	0.0
65	161	190.0	307.39	18.01	1000.0	1177.03	521.12	161.0	164.19	0.0	161.0	164.81	0.0	161.0	163.68	0.0	161.0	164.97	0.0
a1	253	272.0	368.97	7.51	1127.0	1343.65	345.45	258.0	259.97	1.98	258.0	260.1	1.98	257.0	259.84	1.58	258.0	259.81	1.98
a2	252	290.0	415.94	15.08	1129.0	1238.03	348.02	255.0	259.16	1.19	254.0	258.48	0.79	254.0	258.84	0.79	255.0	258.65	1.19
a3	232	261.0	341.61	12.5	1067.0	1189.39	359.91	236.0	239.39	1.72	234.0	239.55	0.86	234.0	239.65	0.86	236.0	239.52	1.72
a4	234	274.0	391.55	17.09	1095.0	1158.55	367.95	235.0	238.81	0.43	235.0	238.74	0.43	236.0	238.97	0.85	236.0	238.9	0.85
a5	236	276.0	368.9	16.95	1082.0	1178.23	358.47	238.0	240.23	0.85	238.0	240.84	0.85	239.0	240.68	1.27	239.0	240.16	1.27
b1	69	76.0	132.94	10.14	1323.0	1436.32	1817.39	69.0	69.06	0.0	69.0	69.16	0.0	69.0	69.23	0.0	69.0	69.1	0.0
b2	76	88.0	143.58	15.79	1364.0	1445.39	1694.74	76.0	76.03	0.0	76.0	76.03	0.0	76.0	76.1	0.0	76.0	76.0	0.0
b3	80	87.0	136.9	8.75	1670.0	1843.23	1987.5	80.0	80.97	0.0	80.0	80.87	0.0	80.0	80.77	0.0	80.0	80.81	0.0
b4	79	88.0	147.35	11.39	88.0	1604.94	11.39	79.0	79.9	0.0	79.0	80.0	0.0	79.0	79.65	0.0	79.0	79.55	0.0
b5	72	72.0	126.97	0.0	1401.0	1480.29	1845.83	72.0	72.29	0.0	72.0	72.65	0.0	72.0	72.23	0.0	72.0	72.16	0.0
c1	227	285.0	346.42	25.55	1508.0	1608.61	564.32	230.0	234.35	1.32	232.0	234.68	2.2	232.0	234.65	2.2	231.0	234.23	1.76
c2	219	259.0	318.06	18.26	1637.0	1783.68	647.49	223.0	226.9	1.83	221.0	225.42	0.91	222.0	226.19	1.37	222.0	225.35	1.37
c3	243	279.0	354.42	14.81	268.0	2063.45	10.29	244.0	247.06	0.41	244.0	247.06	0.41	244.0	247.13	0.41	244.0	246.94	0.41
c4	219	243.0	314.1	10.96	1657.0	1773.26	656.62	221.0	224.58	0.91	221.0	224.29	0.91	222.0	225.13	1.37	221.0	223.77	0.91
c5	215	242.0	306.48	12.56	1537.0	1684.84	614.88	218.0	220.48	1.4	218.0	219.97	1.4	218.0	220.97	1.4	218.0	220.19	1.4
d1	60	69.0	122.19	15.0	1979.0	2093.84	3198.33	60.0	60.9	0.0	60.0	61.19	0.0	60.0	61.06	0.0	60.0	60.9	0.0
d2	66	72.0	125.03	9.09	72.0	2284.97	9.09	66.0	67.03	0.0	66.0	67.03	0.0	66.0	66.94	0.0	66.0	67.0	0.0
d3	72	81.0	145.74	12.5	2369.0	2583.81	3190.28	73.0	73.65	1.39	73.0	73.94	1.39	73.0	73.97	1.39	73.0	73.9	1.39
d4	62	68.0	114.52	9.68	1929.0	2126.94	3011.29	62.0	62.03	0.0	62.0	62.0	0.0	62.0	62.0	0.0	62.0	62.0	0.0
d5	61	77.0	122.55	26.23	1945.0	2114.74	3088.52	61.0	61.48	0.0	61.0	61.52	0.0	61.0	61.39	0.0	61.0	61.23	0.0
		293.98	403.46	15.29	1055.27	1283.87	645.97	255.29	258.14	0.51	255.04	258.17	0.45	255.07	258.17	0.47	255.0	257.81	0.45
		SA_40a_pl0			SA_40a_pl4			SA_80a_pl0			SA_80a_pl4			MAB_40a_pl0			MAB_40a_pl4		
Inst.	Opt.	Best	Avg	RPD	Best	Avg	RPD	Best	Avg	RPD	Best	Avg	RPD	Best	Avg	RPD	Best	Avg	RPD
41	429	430.0	432.65	0.23	430.0	433.26	0.23	430.0	432.77	0.23	430.0	433.16	0.23	430.0	432.77	0.23	430.0	434.16	0.23
42	512	515.0	522.65	0.59	514.0	523.32	0.39	515.0	521.0	0.59	519.0	526.29	1.37	513.0	522.52	0.2	513.0	530.13	0.2
43	516	518.0	522.77	0.39	516.0	522.65	0.0	516.0	521.65	0.0	517.0	524.03	0.19	517.0	522.87	0.19	517.0	527.45	0.19
44	494	494.0	498.94	0.0	494.0	497.97	0.0	494.0	499.0	0.0	496.0	501.61	0.4	495.0	498.35	0.2	497.0	507.55	0.61
45	512	518.0	520.58	1.17	514.0	523.19	0.39	514.0	520.16	0.39	516.0	521.48	0.78	516.0	521.71	0.78	518.0	529.58	1.17
46	560	563.0	565.1	0.54	561.0	565.35	0.18	560.0	564.26	0.0	561.0	565.94	0.18	561.0	564.52	0.18	561.0	568.39	0.18
47	430	430.0	434.68	0.0	431.0	434.39	0.23	431.0	434.29	0.23	432.0	435.77	0.47	431.0	434.61	0.23	434.0	438.97	0.93
48	492	493.0	495.71	0.2	493.0	496.1	0.2	493.0	495.84	0.2	494.0	497.52	0.41	493.0	495.84	0.2	493.0	501.39	0.2
49	641	649.0	661.1	1.25	645.0	661.29	0.62	654.0	661.23	2.03	653.0	664.74	1.87	650.0	661.32	1.4	655.0	671.23	2.18
410	514	514.0	518.16	0.0	514.0	518.23	0.0	514.0	516.65	0.0	514.0	518.84	0.0	514.0	516.94	0.0	514.0	520.52	0.0
51	253	253.0	257.42	0.0	253.0	257.9	0.0	254.0	257.52	0.4	256.0	258.9	1.19	256.0	258.32	1.19	254.0	260.61	0.4
52	302	307.0	317.65	1.66	310.0	317.81	2.65	310.0	317.29	2.65	314.0	321.16	3.97	312.0	316.9	3.31	310.0	323.42	2.65
53	226	228.0	229.61	0.88	228.0	229.29	0.88	228.0	229.45	0.88	228.0	229.77	0.88	228.0	229.55	0.88	228.0	231.03	0.88
54	242	244.0	246.68	0.83	244.0	246.45	0.83	243.0	246.32	0.41	243.0	247.1	0.41	244.0	247.16	0.83	245.0	249.58	1.24
55	211	211.0	212.58	0.0	211.0	213.06	0.0	211.0	212.26	0.0	211.0	213.61	0.0	211.0	212.55	0.0	211.0	213.97	0.0
56	213	213.0	215.71	0.0	213.0	216.16	0.0	213.0	215.06	0.0	213.0	217.48	0.0	213.0	215.42	0.0	213.0	220.52	0.0
57	293	294.0	300.26	0.34	294.0	301.45	0.34	294.0	299.61	0.34	296.0	301.29	1.02	294.0	299.97	0.34	296.0	303.94	1.02
58	288	288.0	289.94	0.0	288.0	289.81	0.0	288.0	289.58	0.0	288.0	290.84	0.0	288.0	290.58	0.0	288.0	292.26	0.0
59	279	279.0	281.16	0.0	279.0	282.16	0.0	280.0	281.0	0.36	279.0	281.61	0.0	280.0	281.26	0.36	281.0	283.61	0.72
510	265	265.0	268.42	0.0	265.0	267.94	0.0	265.0	267.9	0.0	266.0	269.39	0.38	265.0	268.61	0.0	266.0	272.9	0.38
61	138	138.0	141.52	0.0	138.0	141.97	0.0	138.0	141.65	0.0	138.0	142.03	0.0	139.0	141.97	0.72	138.0	143.74	0.0
62	146	146.0	148.68	0.0	146.0	148.29	0.0	146.0	148.03	0.0	146.0	149.87	0.0	146.0	149.1	0.0	147.0	152.23	0.68
63	145	145.0	146.58	0.0	145.0	146.87	0.0	145.0	146.94	0.0	145.0	147.42	0.0	145.0	146.77	0.0	145.0	147.71	0.0
64	131	131.0	131.39	0.0	131.0	132.16	0.0	131.0	131.52	0.0	131.0	132.39	0.0	131.0	132.23	0.0	131.0	133.1	0.0
65	161	161.0	163.65	0.0	161.0	163.84	0.0	161.0	164.06	0.0	161.0	165.55	0.0	161.0	163.16	0.0	161.0	171.45	0.0
a1	253	256.0	260.19	1.19	256.0	259.81	1.19	257.0	259.74	1.58	257.0	260.55	1.58	258.0	260.58	1.98	258.0	263.52	1.98
a2	252	254.0	259.23	0.79	253.0	258.65	0.4	255.0	259.74	1.19	255.0	261.29	1.19	254.0	259.48	0.79	256.0	265.61	1.59
a3	232	236.0	239.35	1.72	236.0	239.74	1.72	237.0	240.0	2.16	234.0	240.39	0.86	233.0	240.29	0.43	235.0	243.03	1.29
a4	234	237.0	239.16	1.28	235.0	239.06	0.43	235.0	239.06	0.43	237.0	240.19	1.28	236.0	238.55	0.85	238.0	244.39	1.71
a5	236	238.0	240.81	0.85	238.0	240.74	0.85	238.0	240.19	0.85	239.0	241.0	1.27	238.0	241.32	0.85	239.0	243.48	1.27
b1	69	69.0	69.29	0.0	69.0	69.23	0.0	69.0	69.13	0.0	69.0	69.35	0.0	69.0	69.26	0.0	69.0	70.77	0.0
b2	76	76.0	76.13	0.0	76.0	76.13	0.0	76.0	76.06	0.0	76.0	76.1	0.0	76.0	76.0	0.0	76.0	77.71	0.0
b3	80	80.0	80.81	0.0	80.0	80.84	0.0	80.0	80.81	0.0	80.0	80.9	0.0	80.0	80.9	0.0	81.0	81.65	1.25
b4	79	79.0	80.23	0.0	79.0	80.26	0.0	79.0	80.03	0.0	79.0	80.42	0.0	79.0	79.94	0.0	79.0	82.13	0.0
b5	72	72.0	72.32	0.0	72.0	72.52	0.0	72.0	72.35	0.0	72.0	72.58	0.0	72.0	72.23	0.0	72.0	73.39	0.0
c1	227	231.0	234.87	1.76	230.0	234.77	1.32	231.0	234.35	1.76	232.0	236.0	2.2	231.0	235.74	1.76	232.0	239.52	2.2
c2	219	221.0	225.84	0.91	221.0	225.45	0.91	222.0	226.61	1.37	222.0	227.48	1.37	222.0	226.77	1.37	224.0	232.23	2.28
c3	243	245.0	247.42	0.82	244.0	248.1	0.41	245.0	246.87	0.82	244.0	248.55	0.41	244.0	248.23	0.41	245.0	252.19	0.82
c4	219	221.0	225.16	0.91	221.0	225.32	0.91	221.0	224.29	0.91	221.0	225.97	0.91	221.0	225.45	0.91	224.0	230.48	2.28
c5	215	219.0	220.68	1.86	217.0	221.65	0.93	217.0	220.03	0.93	219.0	221.26	1.86	219.0	221.45	1.86	220.0	225.77	2.33
d1	60	60.0	61.03	0.0	60.0	61.06	0.0	60.0	60.71	0.0	60.0	61.45	0.0	60.0	61.13	0.0	60.0	62.74	0.0
d2	66	66.0	67.13	0.0	66.0	67.03	0.0	66.0	67.03	0.0	67.0	67.06	1.52	66.0	66.97	0.0	67.0	67.74	1.52
d3	72	72.0	73.52	0.0	72.0	74.23	0.0	72.0	73.35	0.0	73.0	74.32	1.39	72.0	73.71	0.0	73.0	75.94	1.39
d4	62	62.0	62.03	0.0	62.0	62.19	0.0	62.0	62.0	0.0	62.0	62.1	0.0	62.0	62.0	0.0	62.0	62.45	0.0
d5	61	61.0	61.23	0.0	61.0	61.94	0.0	61.0	61.45	0.0	61.0	61.84	0.0	61.0	61.26	0.0	61.0	62.9	0.0
		255.16	258.22	0.45	254.8	258.44	0.36	255.18	257.97	0.46	255.69	259.26	0.66	255.24	258.36	0.5	255.93	261.94	0.79
		MAB_80a_pl0			MAB_80a_pl4			QL_40a_pl0			QL_40a_pl4			QL_80a_pl0			QL_80a_pl4		
Inst.	Opt.	Best	Avg	RPD	Best	Avg	RPD	Best	Avg	RPD	Best	Avg	RPD	Best	Avg	RPD	Best	Avg	RPD
41	429	430.0	432.52	0.23	429.0	432.55	0.0	430.0	432.45	0.23	430.0	432.65	0.23	430.0	432.13	0.23	430.0	432.26	0.23
42	512	516.0	524.13	0.78	515.0	520.32	0.59	515.0	520.97	0.59	513.0	520.52	0.2	515.0	519.71	0.59	514.0	519.58	0.39
43	516	517.0	522.45	0.19	518.0	521.84	0.39	518.0	521.68	0.39	516.0	521.32	0.0	516.0	520.39	0.0	519.0	521.58	0.58
44	494	495.0	500.32	0.2	495.0	496.84	0.2	494.0	497.23	0.0	494.0	496.61	0.0	494.0	497.39	0.0	495.0	498.55	0.2
45	512	516.0	521.19	0.78	515.0	519.23	0.59	514.0	519.03	0.39	514.0	519.32	0.39	514.0	519.45	0.39	516.0	518.84	0.78
46	560	561.0	564.97	0.18	561.0	563.81	0.18	561.0	563.94	0.18	561.0	563.71	0.18	561.0	563.9	0.18	561.0	563.81	0.18
47	430	430.0	434.74	0.0	431.0	434.16	0.23	431.0	434.29	0.23	431.0	434.13	0.23	430.0	433.58	0.0	431.0	433.94	0.23
48	492	493.0	495.68	0.2	493.0	495.65	0.2	493.0	495.74	0.2	493.0	495.29	0.2	493.0	495.32	0.2	493.0	494.87	0.2
49	641	653.0	662.06	1.87	648.0	659.42	1.09	652.0	660.39	1.72	644.0	657.13	0.47	643.0	658.84	0.31	647.0	658.39	0.94
410	514	514.0	516.81	0.0	514.0	516.39	0.0	514.0	516.26	0.0	514.0	516.52	0.0	514.0	517.23	0.0	514.0	516.1	0.0
51	253	254.0	258.1	0.4	254.0	257.74	0.4	254.0	256.58	0.4	254.0	256.68	0.4	254.0	257.03	0.4	253.0	256.06	0.0
52	302	308.0	318.87	1.99	309.0	316.0	2.32	310.0	317.26	2.65	310.0	318.1	2.65	310.0	317.1	2.65	311.0	316.32	2.98
53	226	229.0	229.45	1.33	228.0	229.52	0.88	229.0	229.55	1.33	228.0	229.42	0.88	228.0	229.55	0.88	228.0	229.35	0.88
54	242	242.0	246.87	0.0	243.0	245.71	0.41	242.0	245.71	0.0	242.0	245.39	0.0	244.0	246.19	0.83	242.0	245.35	0.0
55	211	211.0	212.23	0.0	211.0	212.0	0.0	211.0	211.94	0.0	211.0	212.19	0.0	211.0	212.19	0.0	211.0	212.1	0.0
56	213	213.0	215.03	0.0	213.0	214.74	0.0	213.0	214.29	0.0	213.0	214.45	0.0	213.0	214.97	0.0	213.0	215.16	0.0
57	293	294.0	299.97	0.34	294.0	298.87	0.34	295.0	299.35	0.68	295.0	298.68	0.68	295.0	299.58	0.68	294.0	298.45	0.34
58	288	288.0	289.71	0.0	288.0	289.55	0.0	288.0	289.65	0.0	288.0	289.55	0.0	288.0	289.39	0.0	288.0	289.58	0.0
59	279	280.0	281.13	0.36	280.0	281.03	0.36	280.0	281.23	0.36	280.0	280.9	0.36	280.0	281.13	0.36	280.0	280.94	0.36
510	265	265.0	267.94	0.0	265.0	268.03	0.0	265.0	267.39	0.0	265.0	267.48	0.0	265.0	267.35	0.0	265.0	266.74	0.0
61	138	138.0	141.61	0.0	138.0	141.58	0.0	138.0	141.65	0.0	138.0	141.42	0.0	138.0	141.19	0.0	138.0	141.16	0.0
62	146	146.0	148.77	0.0	146.0	147.97	0.0	146.0	147.9	0.0	146.0	148.29	0.0	146.0	147.9	0.0	146.0	148.0	0.0
63	145	145.0	147.16	0.0	145.0	146.42	0.0	145.0	146.39	0.0	145.0	146.06	0.0	145.0	146.42	0.0	145.0	146.06	0.0
64	131	131.0	131.61	0.0	131.0	131.1	0.0	131.0	131.32	0.0	131.0	131.0	0.0	131.0	131.03	0.0	131.0	131.0	0.0
65	161	161.0	162.45	0.0	161.0	163.65	0.0	161.0	162.94	0.0	161.0	162.32	0.0	161.0	162.23	0.0	161.0	162.94	0.0
a1	253	257.0	260.68	1.58	258.0	259.61	1.98	257.0	259.68	1.58	257.0	260.03	1.58	257.0	259.55	1.58	257.0	259.35	1.58
a2	252	255.0	260.0	1.19	253.0	259.03	0.4	254.0	257.97	0.79	254.0	259.13	0.79	253.0	259.06	0.4	254.0	259.06	0.79
a3	232	236.0	239.87	1.72	235.0	239.29	1.29	237.0	239.06	2.16	236.0	238.48	1.72	235.0	238.68	1.29	235.0	239.1	1.29
a4	234	235.0	239.1	0.43	236.0	238.35	0.85	235.0	238.19	0.43	235.0	238.0	0.43	236.0	238.65	0.85	236.0	238.06	0.85
a5	236	239.0	240.48	1.27	238.0	239.94	0.85	238.0	240.1	0.85	238.0	239.94	0.85	238.0	239.97	0.85	238.0	239.77	0.85
b1	69	69.0	69.1	0.0	69.0	69.1	0.0	69.0	69.03	0.0	69.0	69.13	0.0	69.0	69.03	0.0	69.0	69.0	0.0
b2	76	76.0	76.0	0.0	76.0	76.0	0.0	76.0	76.06	0.0	76.0	76.0	0.0	76.0	76.0	0.0	76.0	76.0	0.0
b3	80	80.0	80.87	0.0	80.0	80.9	0.0	80.0	80.84	0.0	80.0	80.77	0.0	80.0	80.77	0.0	80.0	80.74	0.0
b4	79	79.0	79.94	0.0	79.0	79.61	0.0	79.0	79.58	0.0	79.0	79.84	0.0	79.0	79.65	0.0	79.0	79.42	0.0
b5	72	72.0	72.23	0.0	72.0	72.29	0.0	72.0	72.16	0.0	72.0	72.1	0.0	72.0	72.26	0.0	72.0	72.13	0.0
c1	227	230.0	234.55	1.32	230.0	234.29	1.32	228.0	234.42	0.44	230.0	234.1	1.32	231.0	234.71	1.76	230.0	233.61	1.32
c2	219	222.0	227.0	1.37	221.0	225.71	0.91	221.0	225.74	0.91	220.0	224.71	0.46	224.0	226.29	2.28	220.0	226.06	0.46
c3	243	244.0	247.94	0.41	245.0	246.94	0.82	244.0	246.97	0.41	244.0	246.39	0.41	244.0	246.61	0.41	244.0	247.0	0.41
c4	219	221.0	224.52	0.91	221.0	223.68	0.91	221.0	224.68	0.91	220.0	224.0	0.46	221.0	224.0	0.91	221.0	223.61	0.91
c5	215	217.0	221.16	0.93	218.0	220.03	1.4	218.0	220.06	1.4	216.0	219.74	0.47	217.0	220.42	0.93	218.0	220.03	1.4
d1	60	60.0	60.61	0.0	60.0	60.68	0.0	60.0	60.9	0.0	60.0	60.74	0.0	60.0	60.9	0.0	60.0	60.81	0.0
d2	66	66.0	67.1	0.0	66.0	66.77	0.0	67.0	67.0	1.52	66.0	66.87	0.0	66.0	66.77	0.0	66.0	66.9	0.0
d3	72	72.0	73.81	0.0	72.0	73.58	0.0	72.0	73.65	0.0	72.0	73.55	0.0	72.0	73.39	0.0	72.0	73.39	0.0
d4	62	62.0	62.0	0.0	62.0	62.0	0.0	62.0	62.0	0.0	62.0	62.0	0.0	62.0	62.0	0.0	62.0	62.0	0.0
d5	61	61.0	61.45	0.0	61.0	61.23	0.0	61.0	61.32	0.0	61.0	61.16	0.0	61.0	61.16	0.0	61.0	61.16	0.0
		255.18	258.32	0.44	255.04	257.63	0.42	255.13	257.66	0.46	254.76	257.46	0.34	254.93	257.58	0.42	255.02	257.43	0.4

**Table A5:** Comparison of the WOA metaheuristics.

		BCL			MIR			BQSA_40a_pl0			BQSA_40a_pl4			BQSA_80a_pl0			BQSA_80a_pl4		
Inst.	Opt.	Best	Avg	RPD	Best	Avg	RPD	Best	Avg	RPD	Best	Avg	RPD	Best	Avg	RPD	Best	Avg	RPD
41	429	489.0	607.55	13.99	638.0	715.87	48.72	430.0	433.26	0.23	430.0	432.81	0.23	430.0	433.42	0.23	430.0	432.94	0.23
42	512	679.0	852.94	32.62	1079.0	1181.94	110.74	515.0	522.23	0.59	513.0	525.23	0.2	513.0	521.35	0.2	512.0	521.48	0.0
43	516	739.0	884.48	43.22	1222.0	1293.61	136.82	516.0	522.26	0.0	517.0	523.29	0.19	516.0	522.97	0.0	517.0	522.42	0.19
44	494	596.0	779.06	20.65	954.0	1052.03	93.12	494.0	499.48	0.0	494.0	502.87	0.0	495.0	499.58	0.2	495.0	500.77	0.2
45	512	755.0	874.68	47.46	1067.0	1204.13	108.4	516.0	520.29	0.78	514.0	521.84	0.39	514.0	520.32	0.39	515.0	520.84	0.59
46	560	815.0	981.26	45.54	1389.0	1483.13	148.04	563.0	566.39	0.54	561.0	566.06	0.18	561.0	565.13	0.18	562.0	565.39	0.36
47	430	570.0	682.42	32.56	854.0	931.84	98.6	431.0	434.94	0.23	431.0	434.84	0.23	431.0	434.35	0.23	433.0	434.84	0.7
48	492	713.0	875.42	44.92	1148.0	1236.16	133.33	493.0	496.58	0.2	493.0	497.29	0.2	493.0	496.29	0.2	493.0	496.0	0.2
49	641	922.0	1141.55	43.84	1532.0	1706.1	139.0	651.0	663.32	1.56	649.0	662.87	1.25	651.0	660.58	1.56	649.0	663.87	1.25
410	514	696.0	847.0	35.41	1046.0	1180.97	103.5	514.0	518.1	0.0	515.0	518.55	0.19	514.0	517.32	0.0	514.0	517.68	0.0
51	253	347.0	411.1	37.15	543.0	614.74	114.62	254.0	258.55	0.4	254.0	258.1	0.4	253.0	257.58	0.0	254.0	259.23	0.4
52	302	468.0	577.35	54.97	762.0	913.61	152.32	312.0	319.35	3.31	309.0	318.48	2.32	307.0	318.84	1.66	309.0	317.68	2.32
53	226	313.0	377.84	38.5	506.0	558.06	123.89	229.0	229.81	1.33	229.0	230.1	1.33	228.0	229.65	0.88	229.0	229.39	1.33
54	242	318.0	394.97	31.4	540.0	580.19	123.14	245.0	247.03	1.24	243.0	247.16	0.41	243.0	246.52	0.41	243.0	246.9	0.41
55	211	294.0	339.65	39.34	397.0	427.84	88.15	211.0	213.06	0.0	211.0	213.26	0.0	212.0	212.9	0.47	211.0	212.55	0.0
56	213	334.0	389.71	56.81	507.0	544.58	138.03	213.0	216.03	0.0	213.0	216.58	0.0	213.0	216.19	0.0	213.0	216.0	0.0
57	293	387.0	504.06	32.08	642.0	710.52	119.11	294.0	300.06	0.34	295.0	301.26	0.68	295.0	300.32	0.68	295.0	300.45	0.68
58	288	399.0	497.35	38.54	663.0	745.32	130.21	288.0	290.58	0.0	288.0	290.23	0.0	288.0	290.26	0.0	288.0	290.32	0.0
59	279	404.0	497.03	44.8	673.0	740.35	141.22	280.0	282.1	0.36	280.0	281.55	0.36	279.0	281.42	0.0	279.0	281.39	0.0
510	265	390.0	470.13	47.17	594.0	669.58	124.15	265.0	269.42	0.0	265.0	268.71	0.0	265.0	268.26	0.0	265.0	268.74	0.0
61	138	283.0	403.26	105.07	736.0	836.52	433.33	138.0	142.29	0.0	141.0	142.77	2.17	140.0	142.35	1.45	139.0	142.16	0.72
62	146	320.0	561.77	119.18	1100.0	1211.81	653.42	146.0	148.9	0.0	146.0	149.35	0.0	146.0	149.32	0.0	146.0	149.13	0.0
63	145	337.0	537.19	132.41	912.0	1125.97	528.97	145.0	147.0	0.0	145.0	146.94	0.0	145.0	147.03	0.0	145.0	147.13	0.0
64	131	246.0	366.19	87.79	652.0	722.42	397.71	131.0	132.0	0.0	131.0	131.97	0.0	131.0	131.74	0.0	131.0	131.42	0.0
65	161	357.0	531.74	121.74	1020.0	1155.81	533.54	161.0	164.35	0.0	161.0	165.32	0.0	161.0	165.19	0.0	161.0	164.84	0.0
a1	253	455.0	662.71	79.84	1243.0	1352.03	391.3	257.0	260.65	1.58	257.0	260.26	1.58	257.0	259.87	1.58	256.0	259.81	1.19
a2	252	452.0	651.16	79.37	1150.0	1241.0	356.35	255.0	260.55	1.19	255.0	259.84	1.19	255.0	259.84	1.19	254.0	259.55	0.79
a3	232	436.0	601.58	87.93	1066.0	1185.84	359.48	236.0	240.23	1.72	236.0	240.48	1.72	236.0	240.0	1.72	237.0	239.61	2.16
a4	234	467.0	595.97	99.57	1080.0	1161.45	361.54	236.0	239.32	0.85	236.0	239.71	0.85	235.0	239.06	0.43	237.0	238.68	1.28
a5	236	447.0	618.65	89.41	1139.0	1191.23	382.63	238.0	240.61	0.85	238.0	240.81	0.85	238.0	240.71	0.85	239.0	240.61	1.27
b1	69	309.0	561.94	347.83	1344.0	1441.68	1847.83	69.0	69.19	0.0	69.0	69.13	0.0	69.0	69.35	0.0	69.0	69.26	0.0
b2	76	337.0	547.39	343.42	1265.0	1426.32	1564.47	76.0	76.39	0.0	76.0	76.26	0.0	76.0	76.06	0.0	76.0	76.13	0.0
b3	80	378.0	689.84	372.5	1737.0	1848.74	2071.25	80.0	81.03	0.0	80.0	81.1	0.0	80.0	80.9	0.0	80.0	80.87	0.0
b4	79	334.0	631.39	322.78	1514.0	1644.03	1816.46	79.0	80.42	0.0	79.0	80.23	0.0	79.0	79.94	0.0	79.0	80.26	0.0
b5	72	299.0	541.81	315.28	1372.0	1467.52	1805.56	72.0	72.42	0.0	72.0	72.52	0.0	72.0	72.39	0.0	72.0	72.42	0.0
c1	227	523.0	717.65	130.4	1488.0	1610.13	555.51	231.0	234.94	1.76	231.0	234.9	1.76	232.0	235.42	2.2	231.0	234.61	1.76
c2	219	474.0	737.55	116.44	1654.0	1779.23	655.25	223.0	226.68	1.83	222.0	226.26	1.37	221.0	226.71	0.91	221.0	226.19	0.91
c3	243	629.0	919.1	158.85	1970.0	2126.94	710.7	245.0	247.87	0.82	244.0	248.68	0.41	244.0	247.16	0.41	245.0	247.45	0.82
c4	219	570.0	769.13	160.27	1582.0	1734.13	622.37	221.0	225.87	0.91	221.0	225.26	0.91	222.0	224.97	1.37	221.0	224.94	0.91
c5	215	496.0	754.65	130.7	1541.0	1686.84	616.74	218.0	221.35	1.4	218.0	221.32	1.4	218.0	220.97	1.4	217.0	220.94	0.93
d1	60	419.0	801.39	598.33	1950.0	2080.39	3150.0	60.0	61.26	0.0	60.0	61.39	0.0	60.0	61.52	0.0	60.0	61.29	0.0
d2	66	480.0	806.1	627.27	2261.0	2333.68	3325.76	66.0	67.06	0.0	67.0	67.19	1.52	67.0	67.13	1.52	67.0	67.06	1.52
d3	72	506.0	880.23	602.78	2445.0	2581.48	3295.83	72.0	74.03	0.0	73.0	74.74	1.39	73.0	74.0	1.39	73.0	74.06	1.39
d4	62	312.0	755.48	403.23	2025.0	2107.97	3166.13	62.0	62.03	0.0	62.0	62.06	0.0	62.0	62.0	0.0	62.0	62.0	0.0
d5	61	344.0	792.87	463.93	1930.0	2093.29	3063.93	61.0	61.65	0.0	61.0	61.61	0.0	61.0	61.39	0.0	61.0	61.48	0.0
		463.07	653.83	152.83	1176.27	1280.82	778.69	255.38	258.69	0.53	255.22	258.92	0.57	255.13	258.41	0.53	255.22	258.46	0.54
		SA_40a_pl0			SA_40a_pl4			SA_80a_pl0			SA_80a_pl4			MAB_40a_pl0			MAB_40a_pl4		
Inst.	Opt.	Best	Avg	RPD	Best	Avg	RPD	Best	Avg	RPD	Best	Avg	RPD	Best	Avg	RPD	Best	Avg	RPD
41	429	430.0	432.13	0.23	430.0	433.16	0.23	430.0	432.13	0.23	430.0	433.58	0.23	430.0	432.71	0.23	430.0	435.84	0.23
42	512	513.0	524.9	0.2	512.0	526.26	0.0	515.0	522.94	0.59	514.0	528.58	0.39	512.0	520.0	0.0	517.0	534.84	0.98
43	516	517.0	522.35	0.19	517.0	526.16	0.19	516.0	522.9	0.0	520.0	525.84	0.78	516.0	521.13	0.0	522.0	535.26	1.16
44	494	494.0	500.65	0.0	494.0	503.87	0.0	495.0	499.39	0.2	495.0	504.39	0.2	495.0	500.74	0.2	495.0	507.71	0.2
45	512	515.0	522.29	0.59	514.0	524.23	0.39	516.0	520.81	0.78	519.0	523.94	1.37	514.0	522.0	0.39	521.0	532.0	1.76
46	560	561.0	565.68	0.18	561.0	568.97	0.18	561.0	564.9	0.18	563.0	567.74	0.54	562.0	566.35	0.36	560.0	574.52	0.0
47	430	430.0	434.87	0.0	431.0	435.35	0.23	431.0	434.9	0.23	431.0	435.71	0.23	431.0	434.29	0.23	434.0	440.29	0.93
48	492	493.0	496.45	0.2	493.0	497.29	0.2	493.0	496.26	0.2	494.0	497.65	0.41	493.0	496.1	0.2	494.0	502.74	0.41
49	641	651.0	663.9	1.56	647.0	665.74	0.94	648.0	661.03	1.09	657.0	668.16	2.5	647.0	662.97	0.94	661.0	679.32	3.12
410	514	514.0	518.84	0.0	514.0	518.13	0.0	515.0	518.35	0.19	515.0	519.0	0.19	514.0	517.35	0.0	515.0	524.32	0.19
51	253	254.0	258.77	0.4	253.0	259.29	0.0	253.0	256.68	0.0	254.0	260.61	0.4	254.0	257.39	0.4	254.0	263.81	0.4
52	302	311.0	319.06	2.98	307.0	322.39	1.66	307.0	319.0	1.66	312.0	321.77	3.31	306.0	317.68	1.32	317.0	327.42	4.97
53	226	228.0	229.52	0.88	228.0	230.39	0.88	229.0	229.68	1.33	229.0	229.81	1.33	228.0	229.87	0.88	228.0	231.58	0.88
54	242	244.0	247.26	0.83	243.0	246.58	0.41	243.0	246.68	0.41	245.0	248.0	1.24	243.0	246.97	0.41	245.0	249.77	1.24
55	211	211.0	212.71	0.0	211.0	214.1	0.0	211.0	212.9	0.0	211.0	213.94	0.0	211.0	212.71	0.0	212.0	216.06	0.47
56	213	213.0	215.94	0.0	213.0	217.87	0.0	213.0	215.94	0.0	214.0	218.9	0.47	213.0	216.1	0.0	213.0	223.1	0.0
57	293	294.0	301.03	0.34	294.0	299.97	0.34	295.0	300.06	0.68	296.0	301.58	1.02	295.0	300.26	0.68	299.0	307.35	2.05
58	288	288.0	290.19	0.0	288.0	292.42	0.0	288.0	290.1	0.0	288.0	291.32	0.0	288.0	290.55	0.0	290.0	293.39	0.69
59	279	279.0	281.35	0.0	279.0	282.81	0.0	280.0	281.1	0.36	281.0	282.84	0.72	280.0	282.06	0.36	280.0	286.52	0.36
510	265	265.0	269.1	0.0	265.0	269.77	0.0	265.0	268.87	0.0	265.0	270.13	0.0	265.0	269.23	0.0	265.0	273.74	0.0
61	138	138.0	142.26	0.0	139.0	142.29	0.72	139.0	142.06	0.72	140.0	142.68	1.45	138.0	141.97	0.0	139.0	144.58	0.72
62	146	146.0	149.68	0.0	146.0	150.32	0.0	146.0	148.61	0.0	146.0	149.87	0.0	146.0	148.19	0.0	146.0	152.74	0.0
63	145	145.0	147.48	0.0	145.0	147.94	0.0	145.0	147.39	0.0	145.0	147.97	0.0	145.0	147.1	0.0	145.0	149.03	0.0
64	131	131.0	131.84	0.0	131.0	131.84	0.0	131.0	131.71	0.0	131.0	133.0	0.0	131.0	132.32	0.0	132.0	134.29	0.76
65	161	161.0	166.97	0.0	161.0	168.9	0.0	161.0	164.71	0.0	162.0	167.13	0.62	161.0	166.29	0.0	161.0	175.23	0.0
a1	253	256.0	260.55	1.19	257.0	260.61	1.58	258.0	260.35	1.98	259.0	261.65	2.37	258.0	260.71	1.98	258.0	263.65	1.98
a2	252	255.0	260.13	1.19	253.0	260.13	0.4	254.0	259.19	0.79	256.0	262.03	1.59	255.0	261.58	1.19	257.0	267.0	1.98
a3	232	235.0	240.45	1.29	236.0	239.71	1.72	235.0	240.52	1.29	238.0	242.0	2.59	236.0	240.29	1.72	238.0	244.26	2.59
a4	234	235.0	239.42	0.43	235.0	241.1	0.43	237.0	238.97	1.28	237.0	241.39	1.28	236.0	239.39	0.85	237.0	245.77	1.28
a5	236	237.0	241.0	0.42	238.0	241.39	0.85	237.0	240.35	0.42	239.0	241.71	1.27	239.0	241.29	1.27	240.0	244.42	1.69
b1	69	69.0	69.39	0.0	69.0	70.0	0.0	69.0	69.13	0.0	69.0	69.48	0.0	69.0	69.48	0.0	69.0	70.71	0.0
b2	76	76.0	76.16	0.0	76.0	77.16	0.0	76.0	76.06	0.0	76.0	76.52	0.0	76.0	76.39	0.0	76.0	77.74	0.0
b3	80	80.0	80.94	0.0	80.0	81.19	0.0	80.0	80.87	0.0	80.0	81.0	0.0	80.0	81.03	0.0	80.0	81.84	0.0
b4	79	79.0	80.42	0.0	79.0	80.29	0.0	79.0	79.9	0.0	79.0	80.71	0.0	79.0	80.0	0.0	79.0	81.97	0.0
b5	72	72.0	72.58	0.0	72.0	72.65	0.0	72.0	72.52	0.0	72.0	72.58	0.0	72.0	72.39	0.0	72.0	73.35	0.0
c1	227	229.0	234.77	0.88	230.0	235.42	1.32	230.0	234.65	1.32	233.0	235.81	2.64	230.0	235.16	1.32	233.0	243.19	2.64
c2	219	221.0	227.26	0.91	223.0	228.55	1.83	222.0	226.77	1.37	221.0	228.94	0.91	222.0	226.81	1.37	223.0	233.81	1.83
c3	243	244.0	248.48	0.41	244.0	249.23	0.41	245.0	247.26	0.82	246.0	250.1	1.23	244.0	249.32	0.41	245.0	254.81	0.82
c4	219	221.0	225.52	0.91	220.0	226.58	0.46	220.0	225.48	0.46	223.0	226.61	1.83	220.0	225.74	0.46	222.0	229.77	1.37
c5	215	218.0	220.9	1.4	216.0	221.55	0.47	218.0	221.06	1.4	218.0	222.48	1.4	217.0	221.61	0.93	220.0	226.45	2.33
d1	60	60.0	61.68	0.0	60.0	61.61	0.0	60.0	61.45	0.0	60.0	61.71	0.0	60.0	61.58	0.0	60.0	63.32	0.0
d2	66	67.0	67.23	1.52	67.0	67.48	1.52	66.0	67.1	0.0	67.0	67.48	1.52	66.0	67.16	0.0	67.0	68.16	1.52
d3	72	72.0	73.81	0.0	72.0	73.87	0.0	73.0	73.9	1.39	72.0	74.35	0.0	72.0	74.74	0.0	73.0	76.52	1.39
d4	62	62.0	62.13	0.0	62.0	62.19	0.0	62.0	62.16	0.0	62.0	62.06	0.0	62.0	62.16	0.0	62.0	62.68	0.0
d5	61	61.0	61.42	0.0	61.0	61.84	0.0	61.0	61.45	0.0	61.0	61.97	0.0	61.0	61.58	0.0	61.0	64.03	0.0
		255.0	258.88	0.43	254.8	259.75	0.39	255.11	258.41	0.47	256.11	260.1	0.8	254.93	258.68	0.4	256.6	263.75	0.95
		MAB_80a_pl0			MAB_80a_pl4			QL_40a_pl0			QL_40a_pl4			QL_80a_pl0			QL_80a_pl4		
Inst.	Opt.	Best	Avg	RPD	Best	Avg	RPD	Best	Avg	RPD	Best	Avg	RPD	Best	Avg	RPD	Best	Avg	RPD
41	429	430.0	432.97	0.23	430.0	432.94	0.23	430.0	432.58	0.23	430.0	432.26	0.23	430.0	432.42	0.23	430.0	432.71	0.23
42	512	514.0	523.39	0.39	515.0	523.84	0.59	514.0	521.77	0.39	514.0	521.52	0.39	513.0	520.42	0.2	515.0	521.61	0.59
43	516	516.0	521.87	0.0	518.0	522.97	0.39	516.0	520.55	0.0	516.0	521.16	0.0	516.0	521.03	0.0	516.0	522.52	0.0
44	494	494.0	499.35	0.0	495.0	500.94	0.2	495.0	499.0	0.2	495.0	500.16	0.2	494.0	497.58	0.0	495.0	498.19	0.2
45	512	515.0	520.58	0.59	519.0	521.84	1.37	514.0	519.45	0.39	515.0	520.42	0.59	516.0	520.1	0.78	514.0	519.23	0.39
46	560	562.0	565.68	0.36	563.0	565.71	0.54	561.0	564.55	0.18	560.0	564.84	0.0	561.0	564.06	0.18	560.0	563.87	0.0
47	430	431.0	434.23	0.23	433.0	435.19	0.7	431.0	434.29	0.23	431.0	434.23	0.23	431.0	434.26	0.23	430.0	434.03	0.0
48	492	493.0	495.71	0.2	493.0	496.39	0.2	493.0	496.55	0.2	493.0	495.87	0.2	493.0	495.97	0.2	493.0	495.03	0.2
49	641	651.0	661.97	1.56	654.0	664.23	2.03	648.0	659.0	1.09	645.0	659.65	0.62	645.0	657.45	0.62	654.0	661.13	2.03
410	514	514.0	517.19	0.0	514.0	517.94	0.0	515.0	517.03	0.19	514.0	518.42	0.0	514.0	516.0	0.0	514.0	517.1	0.0
51	253	254.0	259.1	0.4	255.0	257.68	0.79	255.0	257.48	0.79	254.0	257.48	0.4	254.0	257.19	0.4	254.0	257.32	0.4
52	302	308.0	318.06	1.99	311.0	319.13	2.98	310.0	317.48	2.65	306.0	316.71	1.32	310.0	316.52	2.65	308.0	317.26	1.99
53	226	228.0	229.55	0.88	228.0	229.45	0.88	228.0	229.45	0.88	228.0	229.42	0.88	228.0	229.48	0.88	228.0	229.61	0.88
54	242	242.0	246.29	0.0	243.0	246.71	0.41	243.0	246.65	0.41	243.0	246.29	0.41	242.0	246.03	0.0	243.0	245.39	0.41
55	211	211.0	212.71	0.0	211.0	212.87	0.0	211.0	212.32	0.0	211.0	212.61	0.0	211.0	212.23	0.0	211.0	212.19	0.0
56	213	213.0	215.26	0.0	213.0	215.74	0.0	213.0	216.03	0.0	213.0	215.1	0.0	213.0	214.94	0.0	213.0	215.32	0.0
57	293	294.0	299.68	0.34	295.0	300.42	0.68	294.0	299.74	0.34	296.0	301.39	1.02	295.0	299.87	0.68	294.0	300.29	0.34
58	288	288.0	290.61	0.0	288.0	289.84	0.0	288.0	289.9	0.0	288.0	290.39	0.0	288.0	290.1	0.0	288.0	289.84	0.0
59	279	280.0	281.68	0.36	280.0	281.48	0.36	280.0	281.16	0.36	280.0	281.42	0.36	280.0	280.87	0.36	280.0	280.84	0.36
510	265	265.0	268.35	0.0	265.0	268.23	0.0	265.0	268.19	0.0	265.0	268.26	0.0	265.0	267.39	0.0	265.0	267.65	0.0
61	138	138.0	142.03	0.0	138.0	142.16	0.0	138.0	141.9	0.0	138.0	141.68	0.0	138.0	141.84	0.0	141.0	141.81	2.17
62	146	147.0	149.1	0.68	146.0	149.1	0.0	146.0	148.58	0.0	146.0	148.68	0.0	146.0	147.87	0.0	146.0	147.77	0.0
63	145	145.0	146.97	0.0	145.0	147.26	0.0	145.0	147.13	0.0	145.0	146.77	0.0	145.0	146.61	0.0	145.0	146.45	0.0
64	131	131.0	131.9	0.0	131.0	131.9	0.0	131.0	131.39	0.0	131.0	131.32	0.0	131.0	131.42	0.0	131.0	131.29	0.0
65	161	161.0	164.65	0.0	161.0	164.58	0.0	161.0	164.55	0.0	161.0	165.45	0.0	161.0	162.65	0.0	161.0	164.19	0.0
a1	253	257.0	260.35	1.58	256.0	260.52	1.19	257.0	259.74	1.58	257.0	260.19	1.58	257.0	260.26	1.58	257.0	259.55	1.58
a2	252	253.0	259.68	0.4	255.0	260.03	1.19	256.0	260.52	1.59	252.0	259.26	0.0	253.0	259.48	0.4	254.0	258.39	0.79
a3	232	236.0	239.61	1.72	237.0	240.39	2.16	236.0	240.16	1.72	237.0	240.55	2.16	235.0	239.26	1.29	236.0	239.77	1.72
a4	234	235.0	238.55	0.43	237.0	239.94	1.28	236.0	239.19	0.85	237.0	239.39	1.28	236.0	238.61	0.85	235.0	238.26	0.43
a5	236	237.0	240.81	0.42	238.0	241.13	0.85	237.0	240.26	0.42	238.0	240.42	0.85	238.0	240.52	0.85	237.0	239.77	0.42
b1	69	69.0	69.29	0.0	69.0	69.58	0.0	69.0	69.29	0.0	69.0	69.35	0.0	69.0	69.1	0.0	69.0	69.13	0.0
b2	76	76.0	76.1	0.0	76.0	76.0	0.0	76.0	76.1	0.0	76.0	76.0	0.0	76.0	76.1	0.0	76.0	76.0	0.0
b3	80	80.0	80.97	0.0	80.0	80.84	0.0	80.0	80.71	0.0	80.0	80.9	0.0	80.0	80.9	0.0	80.0	80.84	0.0
b4	79	79.0	80.0	0.0	79.0	79.87	0.0	79.0	80.16	0.0	79.0	79.9	0.0	79.0	80.1	0.0	79.0	80.06	0.0
b5	72	72.0	72.45	0.0	72.0	72.52	0.0	72.0	72.42	0.0	72.0	72.52	0.0	72.0	72.39	0.0	72.0	72.45	0.0
c1	227	232.0	235.29	2.2	233.0	235.65	2.64	231.0	235.29	1.76	230.0	234.61	1.32	231.0	234.9	1.76	230.0	234.23	1.32
c2	219	222.0	227.06	1.37	223.0	228.06	1.83	223.0	226.94	1.83	222.0	226.77	1.37	222.0	226.71	1.37	223.0	226.29	1.83
c3	243	245.0	248.52	0.82	245.0	247.84	0.82	245.0	247.58	0.82	245.0	247.19	0.82	244.0	246.74	0.41	244.0	247.32	0.41
c4	219	221.0	224.35	0.91	223.0	225.48	1.83	220.0	224.9	0.46	221.0	224.42	0.91	220.0	223.74	0.46	221.0	224.58	0.91
c5	215	217.0	221.03	0.93	218.0	221.48	1.4	219.0	220.74	1.86	218.0	221.1	1.4	218.0	220.52	1.4	217.0	220.68	0.93
d1	60	60.0	61.35	0.0	60.0	61.13	0.0	60.0	61.32	0.0	60.0	61.26	0.0	60.0	61.03	0.0	60.0	61.26	0.0
d2	66	66.0	67.16	0.0	66.0	66.97	0.0	66.0	67.13	0.0	66.0	66.97	0.0	66.0	66.9	0.0	66.0	66.97	0.0
d3	72	72.0	74.19	0.0	73.0	74.0	1.39	72.0	73.77	0.0	73.0	74.0	1.39	73.0	74.1	1.39	72.0	73.55	0.0
d4	62	62.0	62.06	0.0	62.0	62.0	0.0	62.0	62.0	0.0	62.0	62.1	0.0	62.0	62.0	0.0	62.0	62.0	0.0
d5	61	61.0	61.77	0.0	61.0	61.61	0.0	61.0	61.39	0.0	61.0	61.74	0.0	61.0	61.55	0.0	61.0	61.19	0.0
		255.04	258.43	0.42	255.71	258.75	0.64	255.16	258.14	0.48	254.96	258.23	0.44	254.93	257.76	0.43	255.11	257.89	0.46

## Appendix B. Tables of Statical Test

**Table A6:** Average *p*-value of CS compared to other algorithms.

	BQSA_40a_pl0	BQSA_40a_pl4	BQSA_80a_pl0	BQSA_80a_pl4	SA_40a_pl0	SA_40a_pl4	SA_80a_pl0	SA_80a_pl4	MAB_40a_pl0	MAB_40a_pl4	MAB_80a_pl0	MAB_80a_pl4	QL_40a_pl0	QL_40a_pl4	QL_80a_pl0	QL_80a_pl4	BCL	MIR
BQSA_40a_pl0	-	≥0.05	≥0.05	≥0.05	≥0.05	≥0.05	≥0.05	≥0.05	≥0.05	≥0.05	≥0.05	≥0.05	≥0.05	≥0.05	≥0.05	≥0.05	≥0.05	**0.00**
BQSA_40a_pl4	≥0.05	-	≥0.05	≥0.05	≥0.05	≥0.05	≥0.05	≥0.05	≥0.05	≥0.05	≥0.05	≥0.05	≥0.05	≥0.05	≥0.05	≥0.05	≥0.05	**0.00**
BQSA_80a_pl0	≥0.05	≥0.05	-	≥0.05	≥0.05	≥0.05	≥0.05	≥0.05	≥0.05	≥0.05	≥0.05	≥0.05	≥0.05	≥0.05	≥0.05	≥0.05	≥0.05	**0.00**
BQSA_80a_pl4	≥0.05	≥0.05	≥0.05	-	≥0.05	≥0.05	≥0.05	≥0.05	≥0.05	≥0.05	≥0.05	≥0.05	≥0.05	≥0.05	≥0.05	≥0.05	≥0.05	**0.00**
SA_40a_pl0	≥0.05	≥0.05	≥0.05	≥0.05	-	≥0.05	≥0.05	≥0.05	≥0.05	≥0.05	≥0.05	≥0.05	≥0.05	≥0.05	≥0.05	≥0.05	≥0.05	**0.00**
SA_40a_pl4	≥0.05	≥0.05	≥0.05	≥0.05	≥0.05	-	≥0.05	≥0.05	≥0.05	≥0.05	≥0.05	≥0.05	≥0.05	≥0.05	≥0.05	≥0.05	≥0.05	**0.00**
SA_80a_pl0	≥0.05	≥0.05	≥0.05	≥0.05	≥0.05	≥0.05	-	≥0.05	≥0.05	≥0.05	≥0.05	≥0.05	≥0.05	≥0.05	≥0.05	≥0.05	≥0.05	**0.00**
SA_80a_pl4	≥0.05	≥0.05	≥0.05	≥0.05	≥0.05	≥0.05	≥0.05	-	≥0.05	≥0.05	≥0.05	≥0.05	≥0.05	≥0.05	≥0.05	≥0.05	≥0.05	**0.00**
MAB_40a_pl0	≥0.05	≥0.05	≥0.05	≥0.05	≥0.05	≥0.05	≥0.05	≥0.05	-	≥0.05	≥0.05	≥0.05	≥0.05	≥0.05	≥0.05	≥0.05	≥0.05	**0.00**
MAB_40a_pl4	≥0.05	≥0.05	≥0.05	≥0.05	≥0.05	≥0.05	≥0.05	≥0.05	≥0.05	-	≥0.05	≥0.05	≥0.05	≥0.05	≥0.05	≥0.05	≥0.05	**0.00**
MAB_80a_pl0	≥0.05	≥0.05	≥0.05	≥0.05	≥0.05	≥0.05	≥0.05	≥0.05	≥0.05	≥0.05	-	≥0.05	≥0.05	≥0.05	≥0.05	≥0.05	≥0.05	**0.00**
MAB_80a_pl4	≥0.05	≥0.05	≥0.05	≥0.05	≥0.05	≥0.05	≥0.05	≥0.05	≥0.05	≥0.05	≥0.05	-	≥0.05	≥0.05	≥0.05	≥0.05	≥0.05	**0.00**
QL_40a_pl0	≥0.05	≥0.05	≥0.05	≥0.05	≥0.05	≥0.05	≥0.05	≥0.05	≥0.05	≥0.05	≥0.05	≥0.05	-	≥0.05	≥0.05	≥0.05	≥0.05	**0.00**
QL_40a_pl4	≥0.05	≥0.05	≥0.05	≥0.05	≥0.05	≥0.05	≥0.05	≥0.05	≥0.05	≥0.05	≥0.05	≥0.05	≥0.05	-	≥0.05	≥0.05	≥0.05	**0.00**
QL_80a_pl0	≥0.05	≥0.05	≥0.05	≥0.05	≥0.05	≥0.05	≥0.05	≥0.05	≥0.05	≥0.05	≥0.05	≥0.05	≥0.05	≥0.05	-	≥0.05	≥0.05	**0.00**
QL_80a_pl4	≥0.05	≥0.05	≥0.05	≥0.05	≥0.05	≥0.05	≥0.05	≥0.05	≥0.05	≥0.05	≥0.05	≥0.05	≥0.05	≥0.05	≥0.05	-	≥0.05	**0.00**
BCL	≥0.05	≥0.05	≥0.05	≥0.05	≥0.05	≥0.05	≥0.05	≥0.05	≥0.05	≥0.05	≥0.05	≥0.05	≥0.05	≥0.05	≥0.05	≥0.05	-	**0.00**
MIR	≥0.05	≥0.05	≥0.05	≥0.05	≥0.05	≥0.05	≥0.05	≥0.05	≥0.05	≥0.05	≥0.05	≥0.05	≥0.05	≥0.05	≥0.05	≥0.05	≥0.05	-

**Table A7:** Average *p*-value of PSO compared to other algorithms.

	BQSA_40a_pl0	BQSA_40a_pl4	BQSA_80a_pl0	BQSA_80a_pl4	SA_40a_pl0	SA_40a_pl4	SA_80a_pl0	SA_80a_pl4	MAB_40a_pl0	MAB_40a_pl4	MAB_80a_pl0	MAB_80a_pl4	QL_40a_pl0	QL_40a_pl4	QL_80a_pl0	QL_80a_pl4	BCL	MIR
BQSA_40a_pl0	-	≥0.05	≥0.05	≥0.05	≥0.05	≥0.05	≥0.05	≥0.05	≥0.05	≥0.05	≥0.05	≥0.05	≥0.05	≥0.05	≥0.05	≥0.05	≥0.05	**0.00**
BQSA_40a_pl4	≥0.05	-	≥0.05	≥0.05	≥0.05	≥0.05	≥0.05	≥0.05	≥0.05	≥0.05	≥0.05	≥0.05	≥0.05	≥0.05	≥0.05	≥0.05	≥0.05	**0.00**
BQSA_80a_pl0	**0.04**	**0.01**	-	≥0.05	**0.02**	**0.00**	≥0.05	≥0.05	**0.02**	**0.00**	≥0.05	≥0.05	≥0.05	**0.02**	≥0.05	≥0.05	≥0.05	**0.00**
BQSA_80a_pl4	≥0.05	**0.04**	≥0.05	-	**0.04**	**0.00**	≥0.05	≥0.05	**0.04**	**0.00**	≥0.05	≥0.05	≥0.05	**0.05**	≥0.05	≥0.05	≥0.05	**0.00**
SA_40a_pl0	≥0.05	≥0.05	≥0.05	≥0.05	-	≥0.05	≥0.05	≥0.05	≥0.05	≥0.05	≥0.05	≥0.05	≥0.05	≥0.05	≥0.05	≥0.05	≥0.05	**0.00**
SA_40a_pl4	≥0.05	≥0.05	≥0.05	≥0.05	≥0.05	-	≥0.05	≥0.05	≥0.05	≥0.05	≥0.05	≥0.05	≥0.05	≥0.05	≥0.05	≥0.05	≥0.05	**0.00**
SA_80a_pl0	≥0.05	**0.03**	≥0.05	≥0.05	**0.04**	**0.00**	-	≥0.05	**0.03**	**0.02**	≥0.05	≥0.05	≥0.05	**0.05**	≥0.05	≥0.05	≥0.05	**0.00**
SA_80a_pl4	≥0.05	≥0.05	≥0.05	≥0.05	≥0.05	≥0.05	≥0.05	-	≥0.05	≥0.05	≥0.05	≥0.05	≥0.05	≥0.05	≥0.05	≥0.05	≥0.05	**0.00**
MAB_40a_pl0	≥0.05	≥0.05	≥0.05	≥0.05	≥0.05	≥0.05	≥0.05	≥0.05	-	≥0.05	≥0.05	≥0.05	≥0.05	≥0.05	≥0.05	≥0.05	≥0.05	**0.00**
MAB_40a_pl4	≥0.05	≥0.05	≥0.05	≥0.05	≥0.05	≥0.05	≥0.05	≥0.05	≥0.05	-	≥0.05	≥0.05	≥0.05	≥0.05	≥0.05	≥0.05	≥0.05	**0.00**
MAB_80a_pl0	**0.04**	**0.01**	≥0.05	≥0.05	**0.02**	**0.00**	≥0.05	≥0.05	**0.02**	**0.00**	-	**0.04**	≥0.05	**0.03**	≥0.05	≥0.05	≥0.05	**0.00**
MAB_80a_pl4	≥0.05	≥0.05	≥0.05	≥0.05	≥0.05	**0.03**	≥0.05	≥0.05	≥0.05	**0.04**	≥0.05	-	≥0.05	≥0.05	≥0.05	≥0.05	≥0.05	**0.00**
QL_40a_pl0	≥0.05	≥0.05	≥0.05	≥0.05	≥0.05	**0.02**	≥0.05	≥0.05	≥0.05	**0.02**	≥0.05	≥0.05	-	≥0.05	≥0.05	≥0.05	≥0.05	**0.00**
QL_40a_pl4	≥0.05	≥0.05	≥0.05	≥0.05	≥0.05	≥0.05	≥0.05	≥0.05	≥0.05	≥0.05	≥0.05	≥0.05	≥0.05	-	≥0.05	≥0.05	≥0.05	**0.00**
QL_80a_pl0	**0.00**	**0.00**	≥0.05	≥0.05	**0.00**	**0.00**	≥0.05	**0.01**	**0.00**	**0.00**	≥0.05	**0.00**	**0.02**	**0.00**	-	≥0.05	≥0.05	**0.00**
QL_80a_pl4	**0.04**	**0.01**	≥0.05	≥0.05	**0.01**	**0.00**	≥0.05	≥0.05	**0.03**	**0.00**	≥0.05	≥0.05	≥0.05	**0.02**	≥0.05	-	≥0.05	**0.00**
BCL	**0.00**	**0.00**	≥0.05	≥0.05	**0.00**	**0.00**	≥0.05	**0.04**	**0.00**	**0.00**	≥0.05	**0.00**	**0.02**	**0.00**	≥0.05	≥0.05	-	**0.00**
MIR	≥0.05	≥0.05	≥0.05	≥0.05	≥0.05	≥0.05	≥0.05	≥0.05	≥0.05	≥0.05	≥0.05	≥0.05	≥0.05	≥0.05	≥0.05	≥0.05	≥0.05	-

**Table A8:** Average *p*-value of GWO compared to other algorithms.

	BQSA_40a_pl0	BQSA_40a_pl4	BQSA_80a_pl0	BQSA_80a_pl4	SA_40a_pl0	SA_40a_pl4	SA_80a_pl0	SA_80a_pl4	MAB_40a_pl0	MAB_40a_pl4	MAB_80a_pl0	MAB_80a_pl4	QL_40a_pl0	QL_40a_pl4	QL_80a_pl0	QL_80a_pl4	BCL	MIR
BQSA_40a_pl0	-	≥0.05	≥0.05	≥0.05	≥0.05	≥0.05	≥0.05	≥0.05	≥0.05	**0.01**	≥0.05	≥0.05	≥0.05	≥0.05	≥0.05	≥0.05	**0.00**	**0.00**
BQSA_40a_pl4	≥0.05	-	≥0.05	≥0.05	≥0.05	≥0.05	≥0.05	≥0.05	≥0.05	≥0.05	≥0.05	≥0.05	≥0.05	≥0.05	≥0.05	≥0.05	**0.04**	**0.00**
BQSA_80a_pl0	≥0.05	≥0.05	-	≥0.05	≥0.05	**0.03**	≥0.05	≥0.05	≥0.05	**0.00**	≥0.05	≥0.05	≥0.05	≥0.05	≥0.05	≥0.05	**0.00**	**0.00**
BQSA_80a_pl4	≥0.05	≥0.05	≥0.05	-	≥0.05	≥0.05	≥0.05	≥0.05	≥0.05	**0.03**	≥0.05	≥0.05	≥0.05	≥0.05	≥0.05	≥0.05	**0.01**	**0.00**
SA_40a_pl0	≥0.05	≥0.05	≥0.05	≥0.05	-	≥0.05	≥0.05	≥0.05	≥0.05	**0.03**	≥0.05	≥0.05	≥0.05	≥0.05	≥0.05	≥0.05	**0.01**	**0.00**
SA_40a_pl4	≥0.05	≥0.05	≥0.05	≥0.05	≥0.05	-	≥0.05	≥0.05	≥0.05	≥0.05	≥0.05	≥0.05	≥0.05	≥0.05	≥0.05	≥0.05	≥0.05	**0.00**
SA_80a_pl0	≥0.05	≥0.05	≥0.05	≥0.05	≥0.05	**0.04**	-	≥0.05	≥0.05	**0.00**	≥0.05	≥0.05	≥0.05	≥0.05	≥0.05	≥0.05	**0.00**	**0.00**
SA_80a_pl4	≥0.05	≥0.05	≥0.05	≥0.05	≥0.05	**0.04**	≥0.05	-	≥0.05	**0.00**	≥0.05	≥0.05	≥0.05	**0.05**	≥0.05	≥0.05	**0.00**	**0.00**
MAB_40a_pl0	≥0.05	≥0.05	≥0.05	≥0.05	≥0.05	≥0.05	≥0.05	≥0.05	-	**0.02**	≥0.05	≥0.05	≥0.05	≥0.05	≥0.05	≥0.05	**0.00**	**0.00**
MAB_40a_pl4	≥0.05	≥0.05	≥0.05	≥0.05	≥0.05	≥0.05	≥0.05	≥0.05	≥0.05	-	≥0.05	≥0.05	≥0.05	≥0.05	≥0.05	≥0.05	≥0.05	**0.00**
MAB_80a_pl0	≥0.05	**0.02**	≥0.05	≥0.05	≥0.05	**0.02**	≥0.05	≥0.05	≥0.05	**0.00**	-	≥0.05	≥0.05	**0.03**	≥0.05	≥0.05	**0.00**	**0.00**
MAB_80a_pl4	≥0.05	≥0.05	≥0.05	≥0.05	≥0.05	**0.03**	≥0.05	≥0.05	≥0.05	**0.00**	≥0.05	-	≥0.05	≥0.05	≥0.05	≥0.05	**0.00**	**0.00**
QL_40a_pl0	≥0.05	≥0.05	≥0.05	≥0.05	≥0.05	**0.04**	≥0.05	≥0.05	≥0.05	**0.01**	≥0.05	≥0.05	-	≥0.05	≥0.05	≥0.05	**0.00**	**0.00**
QL_40a_pl4	≥0.05	≥0.05	≥0.05	≥0.05	≥0.05	≥0.05	≥0.05	≥0.05	≥0.05	≥0.05	≥0.05	≥0.05	≥0.05	-	≥0.05	≥0.05	**0.02**	**0.00**
QL_80a_pl0	≥0.05	**0.04**	≥0.05	≥0.05	≥0.05	**0.03**	≥0.05	≥0.05	≥0.05	**0.00**	≥0.05	≥0.05	≥0.05	**0.04**	-	≥0.05	**0.00**	**0.00**
QL_80a_pl4	≥0.05	≥0.05	≥0.05	≥0.05	≥0.05	**0.05**	≥0.05	≥0.05	≥0.05	**0.01**	≥0.05	≥0.05	≥0.05	≥0.05	≥0.05	-	**0.00**	**0.00**
BCL	≥0.05	≥0.05	≥0.05	≥0.05	≥0.05	≥0.05	≥0.05	≥0.05	≥0.05	≥0.05	≥0.05	≥0.05	≥0.05	≥0.05	≥0.05	≥0.05	-	**0.03**
MIR	≥0.05	≥0.05	≥0.05	≥0.05	≥0.05	≥0.05	≥0.05	≥0.05	≥0.05	≥0.05	≥0.05	≥0.05	≥0.05	≥0.05	≥0.05	≥0.05	≥0.05	-

**Table A9:** Average *p*-value of SCA compared to other algorithms.

	BQSA_40a_pl0	BQSA_40a_pl4	BQSA_80a_pl0	BQSA_80a_pl4	SA_40a_pl0	SA_40a_pl4	SA_80a_pl0	SA_80a_pl4	MAB_40a_pl0	MAB_40a_pl4	MAB_80a_pl0	MAB_80a_pl4	QL_40a_pl0	QL_40a_pl4	QL_80a_pl0	QL_80a_pl4	BCL	MIR
BQSA_40a_pl0	-	≥0.05	≥0.05	≥0.05	≥0.05	≥0.05	≥0.05	≥0.05	≥0.05	**0.00**	≥0.05	≥0.05	≥0.05	≥0.05	≥0.05	≥0.05	**0.00**	**0.00**
BQSA_40a_pl4	≥0.05	-	≥0.05	≥0.05	≥0.05	≥0.05	≥0.05	≥0.05	≥0.05	**0.00**	≥0.05	≥0.05	≥0.05	≥0.05	≥0.05	≥0.05	**0.00**	**0.00**
BQSA_80a_pl0	≥0.05	≥0.05	-	≥0.05	≥0.05	≥0.05	≥0.05	≥0.05	≥0.05	**0.00**	≥0.05	≥0.05	≥0.05	≥0.05	≥0.05	≥0.05	**0.00**	**0.00**
BQSA_80a_pl4	≥0.05	≥0.05	≥0.05	-	≥0.05	≥0.05	≥0.05	≥0.05	≥0.05	**0.00**	≥0.05	≥0.05	≥0.05	≥0.05	≥0.05	≥0.05	**0.00**	**0.00**
SA_40a_pl0	≥0.05	≥0.05	≥0.05	≥0.05	-	≥0.05	≥0.05	≥0.05	≥0.05	**0.00**	≥0.05	≥0.05	≥0.05	≥0.05	≥0.05	≥0.05	**0.00**	**0.00**
SA_40a_pl4	≥0.05	≥0.05	≥0.05	≥0.05	≥0.05	-	≥0.05	≥0.05	≥0.05	**0.00**	≥0.05	≥0.05	≥0.05	≥0.05	≥0.05	≥0.05	**0.00**	**0.00**
SA_80a_pl0	≥0.05	≥0.05	≥0.05	≥0.05	≥0.05	≥0.05	-	≥0.05	≥0.05	**0.00**	≥0.05	≥0.05	≥0.05	≥0.05	≥0.05	≥0.05	**0.00**	**0.00**
SA_80a_pl4	≥0.05	≥0.05	≥0.05	≥0.05	≥0.05	≥0.05	≥0.05	-	≥0.05	**0.02**	≥0.05	≥0.05	≥0.05	≥0.05	≥0.05	≥0.05	**0.00**	**0.00**
MAB_40a_pl0	≥0.05	≥0.05	≥0.05	≥0.05	≥0.05	≥0.05	≥0.05	≥0.05	-	**0.00**	≥0.05	≥0.05	≥0.05	≥0.05	≥0.05	≥0.05	**0.00**	**0.00**
MAB_40a_pl4	≥0.05	≥0.05	≥0.05	≥0.05	≥0.05	≥0.05	≥0.05	≥0.05	≥0.05	-	≥0.05	≥0.05	≥0.05	≥0.05	≥0.05	≥0.05	**0.00**	**0.00**
MAB_80a_pl0	≥0.05	≥0.05	≥0.05	≥0.05	≥0.05	≥0.05	≥0.05	≥0.05	≥0.05	**0.00**	-	≥0.05	≥0.05	≥0.05	≥0.05	≥0.05	**0.00**	**0.00**
MAB_80a_pl4	≥0.05	≥0.05	≥0.05	≥0.05	≥0.05	≥0.05	≥0.05	≥0.05	≥0.05	**0.00**	≥0.05	-	≥0.05	≥0.05	≥0.05	≥0.05	**0.00**	**0.00**
QL_40a_pl0	≥0.05	≥0.05	≥0.05	≥0.05	≥0.05	≥0.05	≥0.05	≥0.05	≥0.05	**0.00**	≥0.05	≥0.05	-	≥0.05	≥0.05	≥0.05	**0.00**	**0.00**
QL_40a_pl4	≥0.05	≥0.05	≥0.05	≥0.05	≥0.05	≥0.05	≥0.05	≥0.05	≥0.05	**0.00**	≥0.05	≥0.05	≥0.05	-	≥0.05	≥0.05	**0.00**	**0.00**
QL_80a_pl0	≥0.05	≥0.05	≥0.05	≥0.05	≥0.05	≥0.05	≥0.05	≥0.05	≥0.05	**0.00**	≥0.05	≥0.05	≥0.05	≥0.05	-	≥0.05	**0.00**	**0.00**
QL_80a_pl4	≥0.05	≥0.05	≥0.05	≥0.05	≥0.05	≥0.05	≥0.05	≥0.05	≥0.05	**0.00**	≥0.05	≥0.05	≥0.05	≥0.05	≥0.05	-	**0.00**	**0.00**
BCL	≥0.05	≥0.05	≥0.05	≥0.05	≥0.05	≥0.05	≥0.05	≥0.05	≥0.05	≥0.05	≥0.05	≥0.05	≥0.05	≥0.05	≥0.05	≥0.05	-	**0.00**
MIR	≥0.05	≥0.05	≥0.05	≥0.05	≥0.05	≥0.05	≥0.05	≥0.05	≥0.05	≥0.05	≥0.05	≥0.05	≥0.05	≥0.05	≥0.05	≥0.05	≥0.05	-

**Table A10:** Average *p*-value of WOA compared to other algorithms.

	BQSA_40a_pl0	BQSA_40a_pl4	BQSA_80a_pl0	BQSA_80a_pl4	SA_40a_pl0	SA_40a_pl4	SA_80a_pl0	SA_80a_pl4	MAB_40a_pl0	MAB_40a_pl4	MAB_80a_pl0	MAB_80a_pl4	QL_40a_pl0	QL_40a_pl4	QL_80a_pl0	QL_80a_pl4	BCL	MIR
BQSA_40a_pl0	-	≥0.05	≥0.05	≥0.05	≥0.05	≥0.05	≥0.05	≥0.05	≥0.05	**0.00**	≥0.05	≥0.05	≥0.05	≥0.05	≥0.05	≥0.05	**0.00**	**0.00**
BQSA_40a_pl4	≥0.05	-	≥0.05	≥0.05	≥0.05	≥0.05	≥0.05	≥0.05	≥0.05	**0.00**	≥0.05	≥0.05	≥0.05	≥0.05	≥0.05	≥0.05	**0.00**	**0.00**
BQSA_80a_pl0	≥0.05	≥0.05	-	≥0.05	≥0.05	≥0.05	≥0.05	≥0.05	≥0.05	**0.00**	≥0.05	≥0.05	≥0.05	≥0.05	≥0.05	≥0.05	**0.00**	**0.00**
BQSA_80a_pl4	≥0.05	≥0.05	≥0.05	-	≥0.05	≥0.05	≥0.05	≥0.05	≥0.05	**0.00**	≥0.05	≥0.05	≥0.05	≥0.05	≥0.05	≥0.05	**0.00**	**0.00**
SA_40a_pl0	≥0.05	≥0.05	≥0.05	≥0.05	-	≥0.05	≥0.05	≥0.05	≥0.05	**0.00**	≥0.05	≥0.05	≥0.05	≥0.05	≥0.05	≥0.05	**0.00**	**0.00**
SA_40a_pl4	≥0.05	≥0.05	≥0.05	≥0.05	≥0.05	-	≥0.05	≥0.05	≥0.05	**0.00**	≥0.05	≥0.05	≥0.05	≥0.05	≥0.05	≥0.05	**0.00**	**0.00**
SA_80a_pl0	≥0.05	≥0.05	≥0.05	≥0.05	≥0.05	≥0.05	-	≥0.05	≥0.05	**0.00**	≥0.05	≥0.05	≥0.05	≥0.05	≥0.05	≥0.05	**0.00**	**0.00**
SA_80a_pl4	≥0.05	≥0.05	≥0.05	≥0.05	≥0.05	≥0.05	≥0.05	-	≥0.05	**0.00**	≥0.05	≥0.05	≥0.05	≥0.05	≥0.05	≥0.05	**0.00**	**0.00**
MAB_40a_pl0	≥0.05	≥0.05	≥0.05	≥0.05	≥0.05	≥0.05	≥0.05	≥0.05	-	**0.00**	≥0.05	≥0.05	≥0.05	≥0.05	≥0.05	≥0.05	**0.00**	**0.00**
MAB_40a_pl4	≥0.05	≥0.05	≥0.05	≥0.05	≥0.05	≥0.05	≥0.05	≥0.05	≥0.05	-	≥0.05	≥0.05	≥0.05	≥0.05	≥0.05	≥0.05	**0.00**	**0.00**
MAB_80a_pl0	≥0.05	≥0.05	≥0.05	≥0.05	≥0.05	≥0.05	≥0.05	≥0.05	≥0.05	**0.00**	-	≥0.05	≥0.05	≥0.05	≥0.05	≥0.05	**0.00**	**0.00**
MAB_80a_pl4	≥0.05	≥0.05	≥0.05	≥0.05	≥0.05	≥0.05	≥0.05	≥0.05	≥0.05	**0.00**	≥0.05	-	≥0.05	≥0.05	≥0.05	≥0.05	**0.00**	**0.00**
QL_40a_pl0	≥0.05	≥0.05	≥0.05	≥0.05	≥0.05	≥0.05	≥0.05	≥0.05	≥0.05	**0.00**	≥0.05	≥0.05	-	≥0.05	≥0.05	≥0.05	**0.00**	**0.00**
QL_40a_pl4	≥0.05	≥0.05	≥0.05	≥0.05	≥0.05	≥0.05	≥0.05	≥0.05	≥0.05	**0.00**	≥0.05	≥0.05	≥0.05	-	≥0.05	≥0.05	**0.00**	**0.00**
QL_80a_pl0	≥0.05	≥0.05	≥0.05	≥0.05	≥0.05	≥0.05	≥0.05	≥0.05	≥0.05	**0.00**	≥0.05	≥0.05	≥0.05	≥0.05	-	≥0.05	**0.00**	**0.00**
QL_80a_pl4	≥0.05	≥0.05	≥0.05	≥0.05	≥0.05	≥0.05	≥0.05	≥0.05	≥0.05	**0.00**	≥0.05	≥0.05	≥0.05	≥0.05	≥0.05	-	**0.00**	**0.00**
BCL	≥0.05	≥0.05	≥0.05	≥0.05	≥0.05	≥0.05	≥0.05	≥0.05	≥0.05	≥0.05	≥0.05	≥0.05	≥0.05	≥0.05	≥0.05	≥0.05	-	**0.00**
MIR	≥0.05	≥0.05	≥0.05	≥0.05	≥0.05	≥0.05	≥0.05	≥0.05	≥0.05	≥0.05	≥0.05	≥0.05	≥0.05	≥0.05	≥0.05	≥0.05	≥0.05	-
